# Nobiletin ameliorates diabetic nephropathy by modulating TP53-associated PANoptosis: an experimental study

**DOI:** 10.3389/fnut.2026.1816623

**Published:** 2026-09-04

**Authors:** Biwei Zhang, Yi Liu, Leilei Ma, Yingjie Zhao, Yiming Dong, Zhengqi Zhou, Chunyu Tian, Dongjun Wang, Dingjie Xu, Xiaojin La, Hong Chang, Rujie Han, Jiayu Li, Chenxi Wu, Ji’an Li

**Affiliations:** 1 School of Public Health, North China University of Science and Technology, Tangshan, China; 2 College of Traditional Chinese Medicine, North China University of Science and Technology, Tangshan, China; 3 Key Laboratory of Integrated Traditional Chinese and Western Medicine for Diabetes and Its Complications, Tangshan, China

**Keywords:** diabetic nephropathy, nobiletin, PANoptosis, programmed cell death, TP53

## Abstract

**Background:**

Nobiletin (NOB), a naturally occurring polymethoxyflavone enriched in Citri Reticulatae Pericarpium—the dried peel of mature citrus fruit—has attracted increasing attention because of its multi-target pharmacological activities. This study explored the protective effects of NOB on diabetic nephropathy (DN), with a focus on TP53-associated PANoptosis and TFEB-related lysosomal and energy metabolic changes.

**Methods:**

Forty male db/db mice were randomly assigned to the model, metformin, low-dose NOB (30 mg/kg/day), and high-dose NOB (60 mg/kg/day) groups, with 10 mice per group. Ten age-matched male db/m mice served as non-diabetic controls. Treatments were administered by gavage for 8 weeks. Metabolic parameters, lipid profiles, inflammatory cytokines, renal function, histopathology, untargeted metabolomics, energy metabolites, network pharmacology, surface plasmon resonance (SPR), immunofluorescence, qPCR, and Western blot analyses were performed. Supplementary *in vitro* validation was performed in high-glucose-treated HK2 renal tubular epithelial cells with TP53 knockdown or overexpression.

**Results:**

NOB treatment reduced FBG, OGTT-AUC, INS, HOMA-IR, GSP, TC, TG, and LDL-C while increasing HDL-C. High-dose NOB improved renal function, reduced IL-1β and IL-6, and alleviated histological damage. Untargeted metabolomics suggested that NOB partially restored pathway-level changes related to oxidative phosphorylation, lysosomal function, fatty acid metabolism, and energy metabolism. Network pharmacology identified TP53 as a central candidate target, and SPR analysis suggested binding between NOB and TP53 protein. NOB treatment reduced TP53 signal, promoted TFEB nuclear localization, and suppressed pyroptosis-, apoptosis-, and necroptosis-related markers in diabetic kidneys. Representative HK2 Western blot experiments showed expression patterns consistent with attenuation of high-glucose-induced PANoptosis-related protein activation after TP53 knockdown and partial counteraction of the NOB-associated regulatory pattern after TP53 overexpression.

**Conclusion:**

NOB ameliorated DN-related metabolic disturbance, inflammation, renal dysfunction, histological injury, and fibrosis. These protective effects were associated with reduced TP53 activation, restoration of TFEB-related lysosomal and energy metabolic status, and suppression of PANoptosis-related pathways. Supplementary HK2 cell experiments provided supportive, representative evidence for the involvement of TP53 in high-glucose-induced PANoptosis-related protein activation and in the regulatory effect of NOB. These findings suggest that NOB may be a promising natural compound for DN intervention.

## Graphical Abstract

**Figure:**
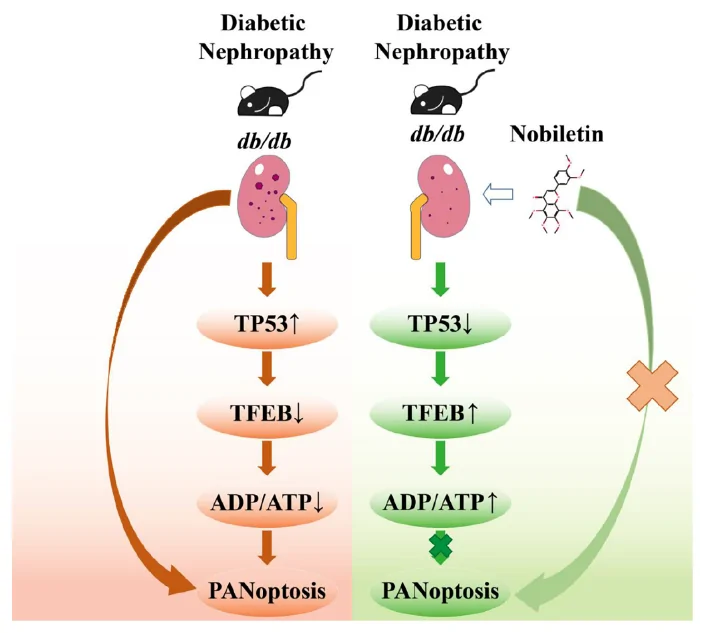
In diabetic nephropathy (db/db mice), elevated TP53 suppresses TFEB signaling, reduces the ADP/ATP ratio, and promotes PANoptosis-associated renal injury. Nobiletin treatment reverses these alterations by downregulating TP53, enhancing TFEB activity, restoring cellular energy homeostasis, and inhibiting PANoptosis, thereby attenuating diabetic nephropathy progression.

## 1 Introduction

Diabetic nephropathy (DN), also known as diabetic kidney disease (DKD), represents one of the most frequent and debilitating microvascular complications of diabetes mellitus (1, 2).

Globally, approximately 700 million cases of end-stage kidney disease are linked to DKD, and its rising prevalence imposes a substantial burden on public health systems. The pathogenesis of DN involves metabolic imbalance, oxidative stress, chronic inflammation, and various forms of programmed cell death (3, 4). Current therapies—including strict glycemic and blood pressure control along with renin–angiotensin–aldosterone system inhibition—delay disease progression but fail to fully prevent renal deterioration, underscoring the need to identify novel pathogenic pathways and therapeutic targets (5).

Beyond classical apoptosis, recent studies have established pyroptosis, necroptosis, and ferroptosis as significant contributors to renal injury in DN (6, 7). These cell death modalities are not mutually exclusive and can occur simultaneously under specific pathological conditions, a phenomenon referred to as PANoptosis (8, 9). PANoptosis is governed by the PANoptosome complex, which integrates apoptotic, pyroptotic, and necroptotic signaling to trigger extensive inflammation and tissue damage (10, 11). Although PANoptosis has been implicated in infectious, ischemia-reperfusion, and cancer models, its activation pattern and upstream regulation in DN remain largely unknown (12). Identifying key molecules associated with PANoptosis and clarifying their regulatory mechanisms are therefore important for advancing DN prevention and management (13).

**FIGURE 1:**
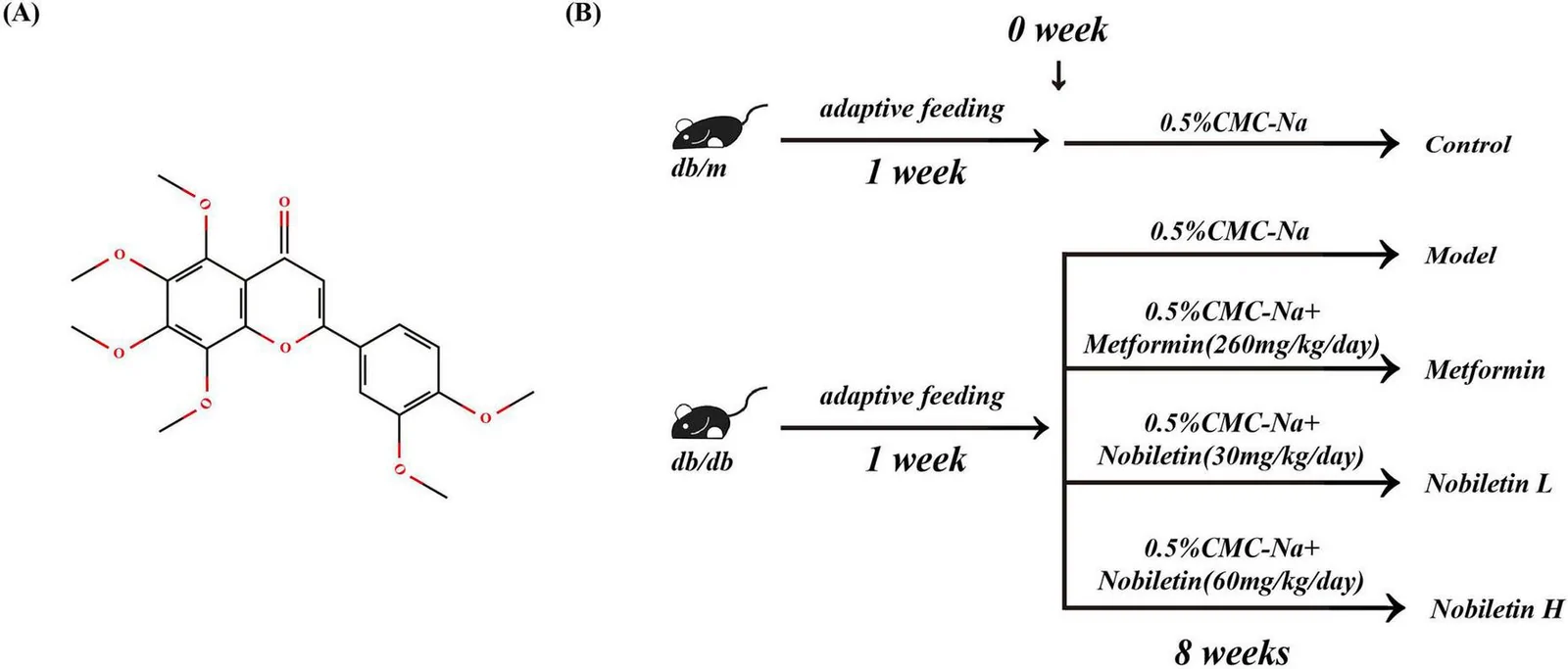
**(A)** Chemical structure of NOB, a polymethoxyflavone isolated from Citri Reticulatae Pericarpium. **(B)** Schematic diagram illustrating the grouping of mice and drug administration protocol.

Nobiletin (NOB, Figure 1A), a naturally occurring polymethoxyflavone found abundantly in Citri Reticulatae Pericarpium—the dried peel of mature citrus fruit—has garnered considerable interest due to its multi-target pharmacological activities (14). NOB exhibits anti-inflammatory, antioxidant, glucose-lipid modulatory, and mitochondrial protective effects (15, 16), mediated in part through AMP-activated protein kinase/sirtuin 1, nuclear factor erythroid 2-related factor 2, and nuclear factor kappa-light-chain-enhancer of activated B cells pathways. It has also demonstrated protective potential in metabolic and renal diseases (17). However, whether NOB confers renoprotection in DN by modulating TP53-associated PANoptosis and metabolic stress remains unclear.

TP53 was considered a biologically plausible candidate because it functions as a central cellular stress-response regulator linking metabolic disturbance, oxidative stress, mitochondrial dysfunction, chronic inflammation, and programmed cell death. In addition to its established role in apoptosis, recent evidence indicates that TP53 participates in non-apoptotic cell-death pathways and may contribute to the coordination of chronic inflammation and PANoptosis-associated responses (18, 19). These properties make TP53 a potential integrative node connecting diabetic metabolic stress with pyroptotic, apoptotic, and necroptotic signaling.

In this study, we used db/db mice as a DN model to evaluate the effects of NOB on renal function, histopathology, and inflammatory responses (20, 21). By integrating metabolomics and network pharmacology, we focused on the TP53-associated PANoptosis axis and assessed changes in key molecules associated with apoptosis, pyroptosis, and necroptosis. Supplementary HK2 cell experiments with TP53 knockdown and overexpression were further performed to support the involvement of TP53 in high-glucose-induced PANoptosis-related protein activation and the regulatory effect of NOB. Our findings aim to clarify a potential mechanism through which NOB ameliorates DN and to provide experimental evidence supporting TP53-associated PANoptosis and metabolic stress as possible therapeutic targets in this condition.

## 2 Materials and methods

### 2.1 Animals

A total of forty specific pathogen-free (SPF) male C57BLKS/J-Lepr db/db mice, aged 8 weeks and weighing 45 ± 5 g, and ten age-matched male heterozygous db/m mice, weighing 25 ± 5 g, were obtained from Changzhou Cavens Laboratory Animal Co., Ltd., (Changzhou, China; license No. SCXK [Su] 2021-0013). All mice were housed under SPF conditions at 25 ± 2 °C and 55 ± 10% relative humidity with a 12 h light/dark cycle and free access to standard chow and water. The animal study was approved by the Animal Ethics Committee of North China University of Science and Technology (Supplementary File 1, Approval No. 2024SY097) and was conducted in accordance with institutional guidelines for animal care and use.

### 2.2 Animal model and drug administration

After a 1-week acclimatization period, fasting blood glucose (FBG) was measured on two non-consecutive days. db/db mice with FBG levels ≥ 11.1 mmol/L in both measurements were considered diabetic and included in the intervention experiment. After model confirmation, eligible db/db mice were numbered and assigned to the DN model, metformin, NOB low-dose, and NOB high-dose groups using a random-number table, with 10 mice per group. The allocation sequence was generated by an independent investigator and placed in sequentially numbered, opaque, sealed envelopes. The investigator responsible for treatment administration opened the corresponding envelope only after each eligible animal had been enrolled, thereby preventing foreknowledge of the upcoming assignment. Sample labels were subsequently coded during endpoint assessment where applicable. The ten db/m mice served as the non-diabetic control group. Thus, the study included five groups. Biological replicates used for each endpoint assay are specified in see section “2.14 Statistical analysis” and in the corresponding figure legends.

Metformin was administered at 260 mg/kg/day as a positive control. NOB (purity 99.31% by HPLC; Certificate of Analysis provided as Supplementary File 2) was suspended in 0.05% carboxymethylcellulose sodium (CMC-Na) and administered by oral gavage at 30 or 60 mg/kg/day for 8 consecutive weeks. The control and DN model groups received an equal volume of 0.05% CMC-Na vehicle (Figure 1B). Body weight and FBG were monitored during the intervention. The 30 and 60 mg/kg/day doses were selected within the oral dose range used in published rodent studies of NOB in inflammatory, neuropathic, cardiorenal, and neurodegenerative injury models (22–25), together with preliminary tolerability considerations. The two-dose design was used to assess dose-related renoprotective responses during the 8-week intervention. No animals were excluded because of death, failed model establishment, insufficient samples, or failed measurements. At the animal-study level, 10 mice were allocated to each group; assay-specific biological replicate numbers are stated in see section “2.14 Statistical analysis” and the corresponding figure legends.

Anesthesia and euthanasia. At the end of the 8-week intervention, mice were anesthetized with isoflurane delivered via a precision vaporizer (induction 3–5%, maintenance 1.5–2.0% in oxygen; O2 flow 0.8–1.0 L/min) using an induction chamber followed by a nose cone. Adequate depth of anesthesia was confirmed by loss of the righting reflex and absence of the pedal withdrawal reflex. Mice were then euthanized by overdose of sodium pentobarbital (80 mg/kg, intraperitoneal injection), followed by cervical dislocation as a secondary method to ensure death. Death was verified by cessation of respiration and heartbeat and absence of corneal and pedal reflexes, after which blood and kidney tissues were collected immediately.

### 2.3 Urine collection, blood Sampling, and tissue processing

One week before sacrifice, each mouse was housed individually in a metabolic cage for 24-h urine collection with free access to food and water. The urine was filtered through a 0.22 μm sterile filter and stored for later analysis.

After the final drug administration, mice were fasted overnight for 12 h with water provided *ad libitum*. Blood was collected immediately after euthanasia and processed using sterile syringes and tubes. Samples were centrifuged at 3,000 × *g* for 10 min at 4 °C to obtain serum. The serum was aliquoted and stored at −80 °C pending biochemical analysis.

Both kidneys were excised and weighed immediately after blood collection. The left kidney was fixed in 4% paraformaldehyde for H&E, PAS, Masson’s trichrome, fibronectin immunohistochemistry, and renal immunofluorescence analyses. The right kidney was snap-frozen in liquid nitrogen and stored at -80 °C for untargeted metabolomics, ATP/ADP measurement, qPCR, Western blotting, and other molecular analyses. Thus, both kidneys from each animal were used, but they were allocated to different assay platforms.

### 2.4 Serum biochemical and inflammatory marker assays

Following an overnight fast, FBG and 2 h postprandial blood glucose levels were measured. An oral glucose tolerance test (OGTT) was conducted at week 8 after completion of the 8-week intervention.

Serum insulin (INS) and C-peptide (C-P) concentrations were quantified with enzyme-linked immunosorbent assay (ELISA) kits, following the manufacturer’s protocol. Homeostasis model assessment of insulin resistance (HOMA-IR) was calculated using the formula:


HOMA-IR=[FBG⁢(mmol/L)×FINS⁢(μ⁢U/mL)]/22.5

Glycosylated serum protein (GSP) was also detected using an ELISA kit. Serum inflammatory cytokines, including interleukin-1β (IL-1β) and interleukin-6 (IL-6), were quantified via ELISA.

Lipid profile parameters, including total cholesterol (TC), triglycerides (TG), low-density lipoprotein cholesterol (LDL-C), and high-density lipoprotein cholesterol (HDL-C), as well as renal function indicators, including serum creatinine (SCR), uric acid (UA), and blood urea nitrogen (BUN), were measured using commercial biochemical assay kits. Urinary protein (UPRO) levels were determined using a colorimetric assay kit.

### 2.5 Histopathological examination of renal tissue

Renal tissues were fixed in 4% paraformaldehyde, dehydrated, embedded in paraffin, and sectioned at 4 μm thickness. Hematoxylin-eosin (H&E) staining was performed to examine the general morphology of the kidney tissue. Periodic acid-Schiff (PAS) staining was used to assess glycogen deposition, and Masson’s trichrome staining was conducted to further evaluate the degree of renal fibrosis. Before quantitative histological and immunostaining assessment, slides and digital images were assigned coded identifiers; the investigator performing image quantification was not informed of treatment-group identity during analysis.

### 2.6 Immunohistochemical staining for fibronectin in renal tissue

Paraffin-embedded kidney tissue sections (4 μm thick) were deparaffinized, rehydrated, and subjected to heat-induced antigen retrieval. Endogenous peroxidase activity was blocked, followed by blocking with normal serum. The sections were incubated with primary antibody against fibronectin at 4°C overnight. After washing, HRP-conjugated secondary antibody was added and incubated at room temperature for 1h. Visualization was performed with 3,3’-diaminobenzidine (DAB), and sections were counterstained with hematoxylin, dehydrated, and mounted with neutral resin. Stained sections were observed under an inverted light microscope. The staining intensity of fibronectin was quantified using ImageJ image analysis software.

### 2.7 Untargeted metabolomics

Samples were thawed at 4°C, vortexed for 1 min, and an aliquot was transferred to a 2 mL tube. 300 μL of internal standard solution (4 ppm 2-chlor-L-phenylalanine in 80% methanol) was added, vortexed for 1 min, and centrifuged at 12,000rpm for 10 min at 4 °C. The supernatant was filtered (0.22 μm) and subjected to LC-MS analysis.

LC was performed on a Vanquish UHPLC system (Thermo Fisher Scientific) with a Waters ACQUITY UPLC^®^ HSS T3 column (2.1 × 100 mm, 1.8 μm). Column temperature: 40°C; flow rate: 0.3 mL/min; injection volume: 5 μL. Positive mode mobile phases: 0.1% formic acid in water (A2) and in acetonitrile (B2) with the gradient: 0–1 min, 10% B2; 1–5 min, 10–98% B2; 5–6.5 min, 98% B2; 6.5–6.6 min, 98–10% B2; 6.6–8 min, 10% B2. Negative mode used acetonitrile (B3) and 5 mM ammonium formate (A3) with the same gradient.

MS analysis was conducted on an Orbitrap Exploris 120 (Thermo Fisher Scientific) in full-scan/data-dependent MS2 mode (m/z 100–1000, resolution 60,000; MS2 resolution 15,000; spray voltage + 3.50kV and -2.50kV; capillary temperature 325°C; NCE 30%).

Data were processed with the ropls R package (v1.22.0) using PLS-DA and OPLS-DA. All biological samples were prepared and acquired within one LC-MS analytical batch; therefore, no inter-batch correction algorithm was applied. Internal standards and pooled quality-control samples inserted throughout acquisition were used to monitor analytical performance. *P*-values were adjusted for multiple testing to control the false discovery rate (FDR). Differential metabolites were defined as VIP > 1.0, FDR-adjusted *P* < 0.05, and FC > 1.0 for increased metabolites or FC < 1.0 for decreased metabolites. Pathway enrichment was performed via a hypergeometric test using the KEGG database and visualized with KEGG Mapper.

### 2.8 Network pharmacology

To identify potential targets of nobiletin in DN, TCMSP (v1.0; accessed October 19, 2024; oral bioavailability ≥ 30% and drug-likeness ≥ 0.18), SwissTargetPrediction (accessed October 19, 2024), and PharmMapper (accessed October 17, 2024) were used to retrieve or predict NOB-related targets. DN-related genes were collected from GeneCards (accessed October 17, 2024; relevance score at or above the median), OMIM (accessed March 5, 2025), and the Therapeutic Target Database (TTD; accessed March 5, 2025) using the keywords “diabetic nephropathy” and “diabetic kidney disease.”

All targets were standardized using UniProt (accessed March 7, 2025), and duplicate entries were removed. Venn diagrams were used to identify overlapping NOB- and DN-related targets. Protein-protein interaction networks were generated using STRING data retrieved on March 12, 2025, with a minimum required interaction score of 0.9, and were visualized in Cytoscape (v3.10.0). Gene Ontology and KEGG pathway enrichment analyses were performed using DAVID. Database access dates and screening thresholds were recorded to improve reproducibility.

### 2.9 Immunofluorescence co-localization of TFEB and TP53 in renal tissue

Paraffin-embedded kidney sections were processed according to the instructions of a three-color immunofluorescence kit, including baking, deparaffinization, rehydration, antigen retrieval, peroxidase blocking, and serum blocking. Sections were incubated with TFEB primary antibody at 4°C overnight, followed by repeated processing and incubation with TP53 primary antibody at 4°C overnight. After a final high-temperature antigen retrieval, sections were mounted using anti-fade mounting medium containing DAPI. Fluorescence imaging was performed using a laser confocal microscope, and fluorescence intensity was quantified using ImageJ software.

### 2.10 Surface plasmon resonance (SPR) analysis

SPR was conducted to determine binding kinetics and affinity. Recombinant TP53 protein (P0358, Fine Test) was diluted to 30 μg/mL in immobilization buffer (1 × PBS-EP, pH7.4) and immobilized in the flow cell via amine coupling. Nobiletin (T6648, TOPSCIENCE) was dissolved in DMSO (V900090, Sigma-Aldrich) and serially diluted in PBS-EP containing 5% DMSO to final concentrations of 25, 12.5, 6.25, 3.125, 1.5625, and 0.78125 μM. Samples were injected at a flow rate of 30 μL/min, with an association phase of 60s and dissociation phase of 600s. Sensor surfaces were regenerated with 10mM glycine-HCl (pH2.0). Data were collected using BIAcore T200 control software and analyzed by a steady-state affinity model.

### 2.11 Western blot analysis of PANoptosis-related proteins

Renal cortical tissues were lysed in RIPA buffer containing protease and phosphatase inhibitor cocktails to extract total protein. Protein samples were separated on 10% or 12.5% SDS-PAGE gels and transferred to PVDF membranes. Membranes were blocked with 5% non-fat milk, incubated with primary antibodies (Supplementary Table 1) at 4°C overnight, and then with HRP-conjugated secondary antibodies at room temperature for 1 h. Target protein bands were visualized using an ECL detection kit, and band intensity was quantified with ImageLab software. β-Actin served as the internal loading control.

### 2.12 RNA extraction and quantitative real-time PCR analysis of PANoptosis-related genes

Total RNA from kidney tissues was extracted using Trizon NC reagent (GLPBIO, USA). cDNA synthesis and qRT-PCR were performed with commercial reverse transcription and SYBR Green PCR kits (GoldenBett, Beijing, China). Primer sequences are listed in Supplementary Table 2. β-Actin was used as the reference gene, and relative expression levels were calculated using the 2-ΔΔCt method.

### 2.13 Supplementary *in vitro* TP53 knockdown and overexpression in HK2 cells

HK2 human renal tubular epithelial cells were used for supplementary TP53 functional validation. To establish an *in vitro* diabetic stress model, HK2 cells were exposed to high glucose (HG), and NOB treatment conditions for subsequent experiments were selected according to the CCK-8 cell viability assay shown in Supplementary Figure 2. The specific treatment groups used for the cell validation experiments are indicated in Supplementary Figures 1–3. HK2 cells were transduced with commercial lentiviral vectors carrying either TP53 short hairpin RNA (SH-TP53), TP53 overexpression sequence (OE-TP53), or the corresponding negative controls (SH-NC and OE-NC). The vectors contained a GFP reporter and a puromycin resistance cassette, and puromycin selection was used to enrich successfully transduced cells according to the manufacturer’s protocol. Transduction efficiency was evaluated by GFP fluorescence, and TP53 knockdown or overexpression efficiency was confirmed by Western blotting. Protein expression levels of TP53, ASC, Caspase-1, Caspase-8, NLRP3, BAX, RIPK1, p-RIPK1, ZBP1, and TFEB were detected by Western blotting. β-Actin was used as the internal loading control. These *in vitro* experiments were included as supplementary functional validation to support, but not overextend, the *in vivo* mechanistic findings.

### 2.14 Statistical analysis

All quantitative data are expressed as the mean ± standard error of the mean (SEM). Statistical analyses were performed using SPSS v23.0 (IBM, USA) and GraphPad Prism v9.5.0 for Windows (GraphPad Software, San Diego, CA, USA). Normality was assessed using the Shapiro-Wilk test, and homogeneity of variance was evaluated using Levene’s or Brown-Forsythe test. For normally distributed data with equal variances, one-way ANOVA followed by Tukey’s *post-hoc* test was used. For normally distributed data with unequal variances, Welch’s ANOVA followed by an appropriate multiple-comparison test was used. For non-normally distributed data, the Kruskal-Wallis test followed by Dunn’s multiple-comparison test was applied. Time-course data, including body weight, fasting blood glucose, and OGTT curves, were analyzed using repeated-measures ANOVA or mixed-effects analysis where appropriate. For analyses of metabolite relative abundance, nonparametric tests including the Wilcoxon rank-sum test and Mann-Whitney U test were employed where appropriate. A two-sided *P*-value < 0.05 was considered statistically significant. Assay-specific biological replicate numbers were as follows: serum metabolic, lipid, renal-function, inflammatory, and renal ATP/ADP measurements, *n* = 6 per group; untargeted renal metabolomics, *n* = 5 per group; renal histology, fibronectin immunohistochemistry, and renal immunofluorescence, *n* = 3 animals per group; renal qPCR, *n* = 5 per group; renal Western blotting, *n* = 3 per group; and supplementary HK2 CCK-8 experiments, three independent experiments. Representative Western blot images are presented for the supplementary HK2 TP53 validation. Network pharmacology analyses do not have an animal-level n, and the SPR assay used six NOB concentrations to estimate binding kinetics rather than a between-group biological comparison.

## 3 Results

### 3.1 Glycemic control and metabolic improvements following nobiletin intervention in db/db mice

**FIGURE 2:**
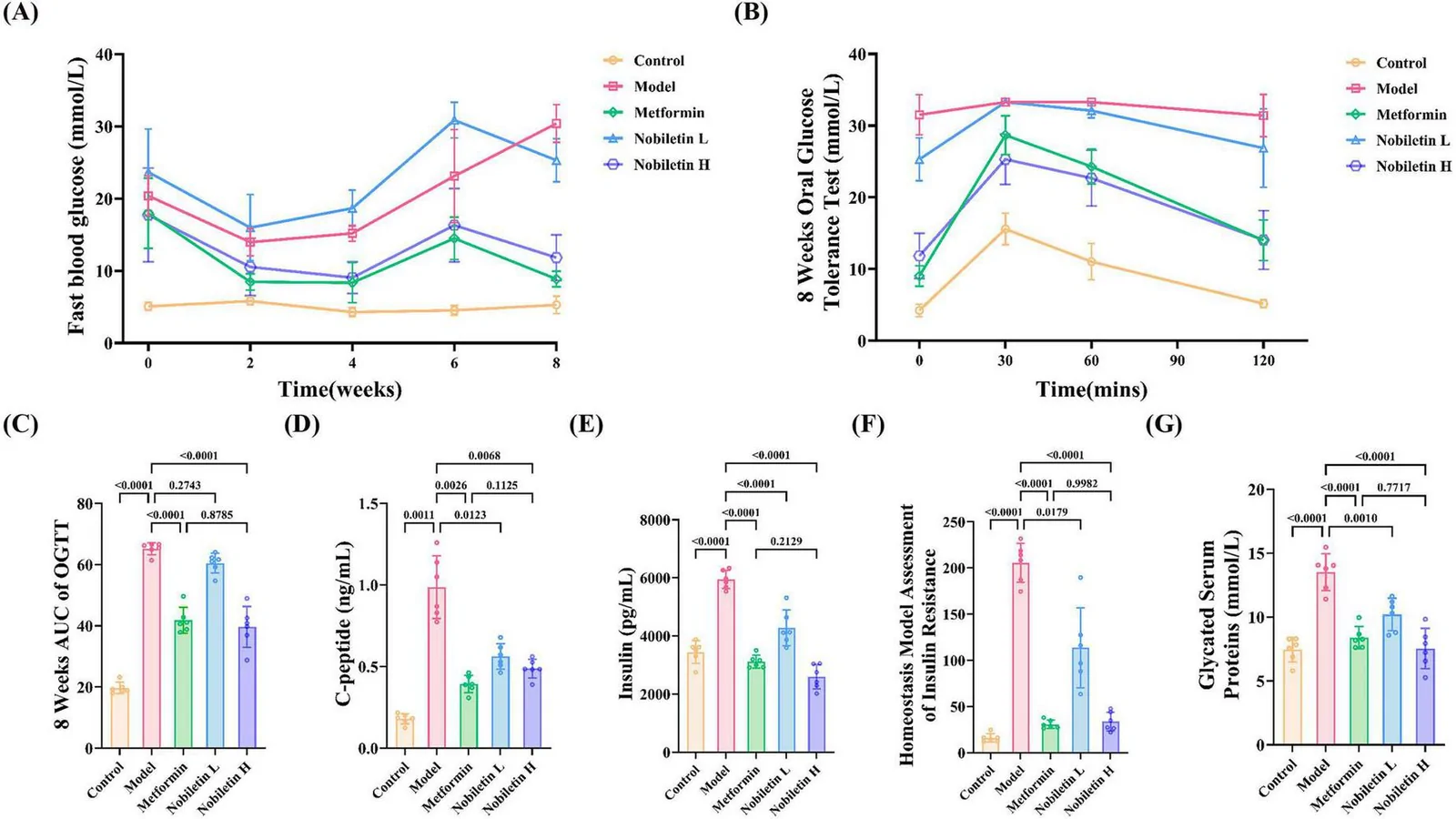
Effects of Nobiletin on glycemic control and metabolic parameters in db/db mice over an 8-week intervention period. **(A)** FBG levels measured biweekly; **(B)** oral glucose tolerance test (OGTT) at week 8; **(C)** area under the curve (AUC) of OGTT; **(D)** serum C-peptide; **(E)** serum insulin; **(F)** homeostatic model assessment of insulin resistance (HOMA-IR); **(G)** glycated serum protein (GSP). Data are presented as mean ± SEM (*n* = 6 per group for biochemical analyses). Time-course data were analyzed by repeated-measures ANOVA or mixed-effects analysis where appropriate; endpoint data were analyzed by one-way ANOVA followed by Tukey’s *post-hoc* test or an appropriate alternative test according to data distribution and variance homogeneity. Exact two-sided *P*-values are displayed above the corresponding comparison brackets, and each bracket identifies the groups compared.

During the 8-week intervention period, significant differences in metabolic parameters were observed among groups (Figures 2A–G). The diabetic nephropathy model group exhibited marked hyperglycemia, characterized by significantly elevated FBG, a higher peak in the oral glucose tolerance test (OGTT) curve at 30 min, and sustained elevation at 120 min (*P* < 0.0001). The area under the curve (AUC) for OGTT was also significantly increased (*P* < 0.0001). Both metformin and NOB treatment markedly improved hyperglycemic status, as evidenced by reduced FBG, lower OGTT peaks with faster declines, and decreased AUC. High-dose NOB exerted greater improvements than low-dose NOB, comparable to metformin. The low-dose group showed partial improvement, with some parameters not reaching statistical significance, indicating a dose-dependent effect. Model mice exhibited significantly elevated C-peptide and insulin levels (*p* < 0.0001), suggesting compensatory hypersecretion from pancreatic β-cells and pronounced insulin resistance. Both metformin and high-dose NOB significantly reduced C-peptide and insulin concentrations, as well as the homeostatic model assessment of insulin resistance (HOMA-IR) (NOB-H and metformin *p* < 0.0001; NOB-L *p* < 0.05). Glycated serum protein (GSP) was markedly increased in the model group (*P* < 0.0001) and significantly decreased by metformin and high-dose NOB (*P* < 0.0001), with low-dose NOB also lowering GSP (*P* = 0.001).

Taken together, these findings indicate that NOB confers dose-dependent hypoglycemic effects in db/db mice, improves both fasting and postprandial glycemic control, attenuates β-cell hypersecretion, and enhances insulin sensitivity, supporting its potential therapeutic role in glycemic regulation.

### 3.2 Effects of nobiletin on body weight, renal index, and lipid profile in db/db mice

**FIGURE 3:**
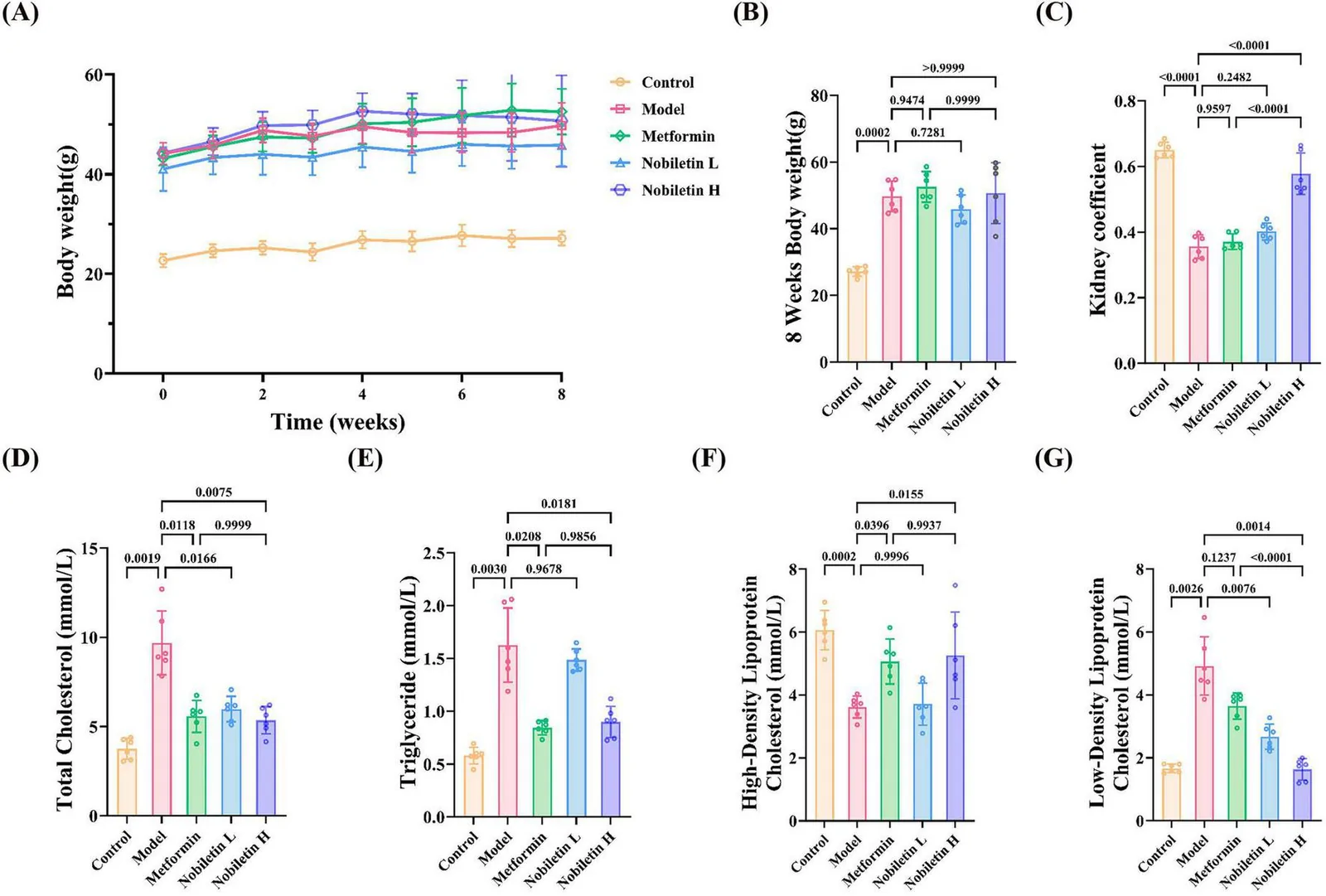
Effects of nobiletin on body weight, kidney coefficient, and serum lipid profile in db/db mice during an 8-week intervention. **(A)** Body weight changes over 8 weeks; **(B)** final body weight at week 8; **(C)** kidney coefficient [kidney weight/body weight × 100%]; **(D)** serum total cholesterol (TC); **(E)** serum triglycerides (TG); **(F)** high-density lipoprotein cholesterol (HDL-C); **(G)** low-density lipoprotein cholesterol (LDL-C). Data are presented as mean ± SEM (*n* = 6 per group for the analyzed endpoints). Time-course data were analyzed by repeated-measures ANOVA or mixed-effects analysis where appropriate; endpoint data were analyzed by one-way ANOVA followed by Tukey’s *post-hoc* test or an appropriate alternative test. Exact two-sided *P-*values are displayed above the corresponding comparison brackets, and each bracket identifies the groups compared.

During the 8-week experimental period, body weight changes in each group are shown in Figure 3A. The control group maintained significantly lower body weight compared with all other groups throughout the study (*p* < 0.0001), whereas the model group (Model), metformin group (Metformin), and both low-dose and high-dose nobiletin groups (NOB-L, NOB-H) displayed gradual weight gain over time, with no significant differences among intervention groups. At week 8 (Figure 3B), body weight in the model group was significantly higher than in the control group (*p* = 0.0002), but no significant differences were observed between the metformin, NOB-L, or NOB-H groups and the model group (*p* > 0.05).

Analysis of the renal index [kidney weight (g)/body weight (g) × 100%] (Figure 3C) revealed a significant increase in the model group compared to controls (*p* < 0.0001), indicating a relative enlargement of the kidney under diabetic conditions. Both metformin and NOB-H markedly reduced the renal index (*p* < 0.0001), whereas NOB-L showed a non-significant downward trend (*p* > 0.05).

Lipid profile analysis (Figures 3D–G) demonstrated that the model group exhibited significantly elevated total cholesterol (TC, *p* = 0.0019), triglycerides (TG, *p* = 0.0030), and low-density lipoprotein cholesterol (LDL-C, *p* = 0.0026), along with decreased high-density lipoprotein cholesterol (HDL-C, *p* = 0.0002). Both metformin and NOB-H significantly reduced TC (*p* = 0.0118; *p* = 0.0166), TG (*p* = 0.0208; *p* = 0.0181), and LDL-C (*p* = 0.0076; *p* = 0.0014), while increasing HDL-C (*p* = 0.0396; *p* = 0.0155). The NOB-L group significantly improved TC (*p* = 0.0075), but showed no statistical changes in TG, HDL-C, or LDL-C (*p* > 0.05).

Overall, nobiletin effectively ameliorated dyslipidemia in db/db mice, with high-dose treatment showing pronounced improvements in reducing TC, TG, and LDL-C while increasing HDL-C, with efficacy in several parameters comparable to metformin. Additionally, NOB-H significantly improved the renal index, suggesting potential benefits in protecting renal structure and mitigating diabetes-related metabolic disturbances.

### 3.3 Renal protective and anti-inflammatory effects of nobiletin in db/db mice

To investigate the effects of NOB on renal function and inflammatory response in db/db mice, serum creatinine (SCr), urinary protein (UP), blood urea nitrogen (BUN), uric acid (UA), interleukin-1β (IL-1β), and interleukin-6 (IL-6) were measured.

**FIGURE 4:**
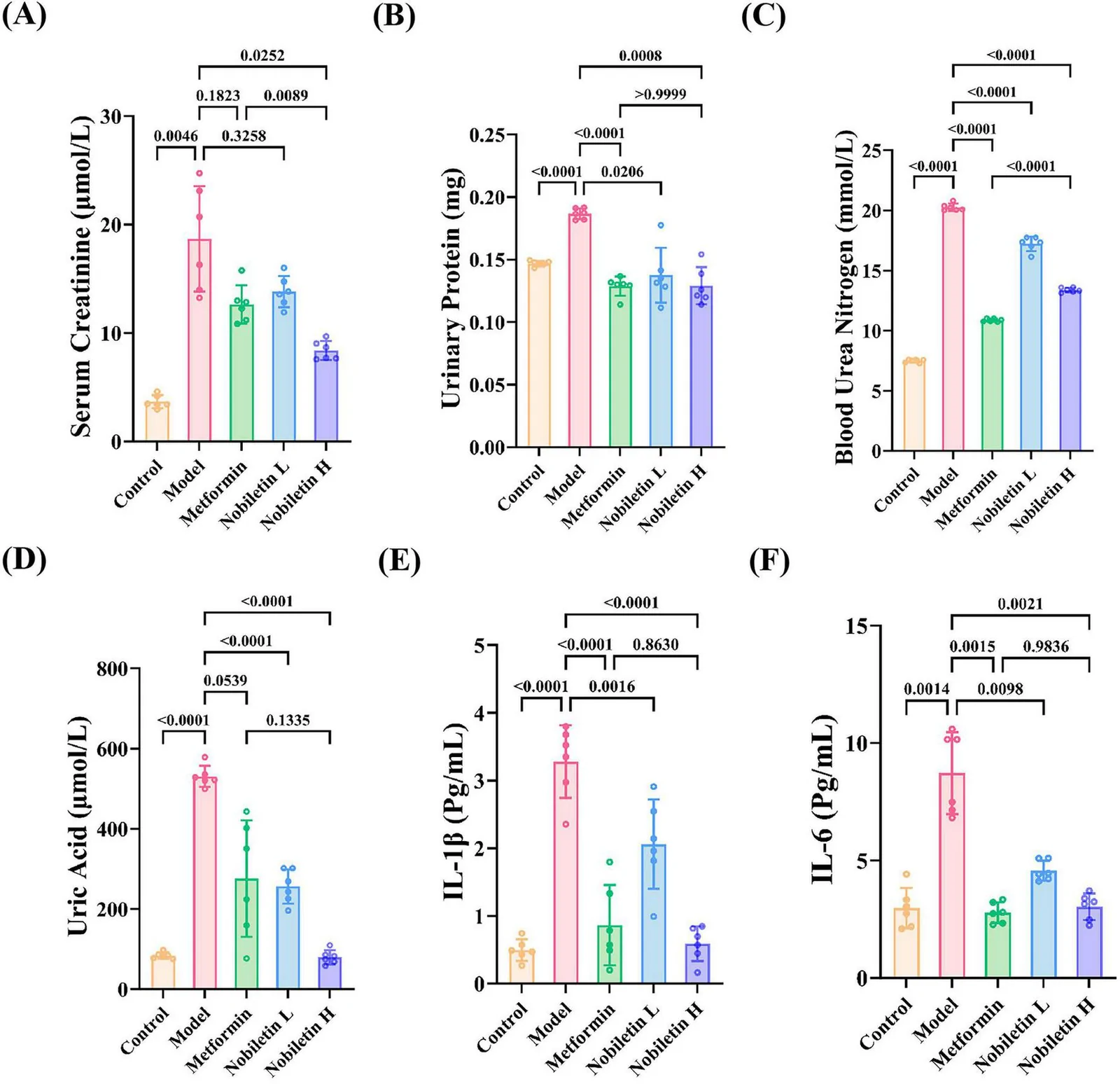
Effects of nobiletin on renal function and inflammatory cytokines in db/db mice. **(A)** Serum creatinine (SCr); **(B)** urinary protein (UP); **(C)** blood urea nitrogen (BUN); **(D)** uric acid (UA); **(E)** interleukin-1β (IL-1β); **(F)** interleukin-6 (IL-6). Data are presented as mean ± SEM (*n* = 6 per group). Statistical significance was determined by one-way ANOVA followed by Tukey’s *post-hoc* test or an appropriate alternative test according to data distribution and variance homogeneity. Exact two-sided *P*-values are displayed above the corresponding comparison brackets, and each bracket identifies the groups compared.

Renal function parameters (Figures 4A–D) showed that the model group (Model) had significantly higher SCr, UP, BUN, and UA levels than the control group (Control) (*p* < 0.0001 or *p* < 0.01), indicating evident renal injury and metabolic disturbance under diabetic conditions. Metformin markedly reduced all of these parameters. High-dose NOB (NOB-H) significantly improved most markers, with pronounced reductions in SCr (*p* = 0.0089), UP (*p* = 0.0206), and BUN (*p* < 0.0001), and a modest decrease in UA. Low-dose NOB (NOB-L) significantly lowered BUN (*p* < 0.0001) but showed no significant improvement in other indices.

Regarding inflammatory cytokines (Figures 4E,F), IL-1β and IL-6 levels were markedly elevated in the model group (IL-1β: *p* < 0.0001; IL-6: *p* = 0.0014), reflecting persistent systemic inflammation in diabetes. Metformin significantly reduced IL-1β (*p* < 0.0001) and IL-6 (*p* = 0.0021). NOB-H similarly decreased IL-1β (*p* = 0.0016) and IL-6 (*p* = 0.0098), whereas NOB-L exhibited a non-significant downward trend (*p* > 0.05).

Collectively, these results demonstrate that the model group developed notable renal dysfunction and elevated inflammatory cytokines. High-dose NOB effectively attenuated renal abnormalities (reducing SCr, UP, BUN, UA) and suppressed IL-1β and IL-6 overexpression, with efficacy in several parameters approaching that of metformin. These findings suggest a potential therapeutic and preventive role of NOB in diabetic renal protection and anti-inflammatory intervention.

### 3.4 Histopathological and fibrotic protective effects of nobiletin in the kidneys of db/db mice

**FIGURE 5:**
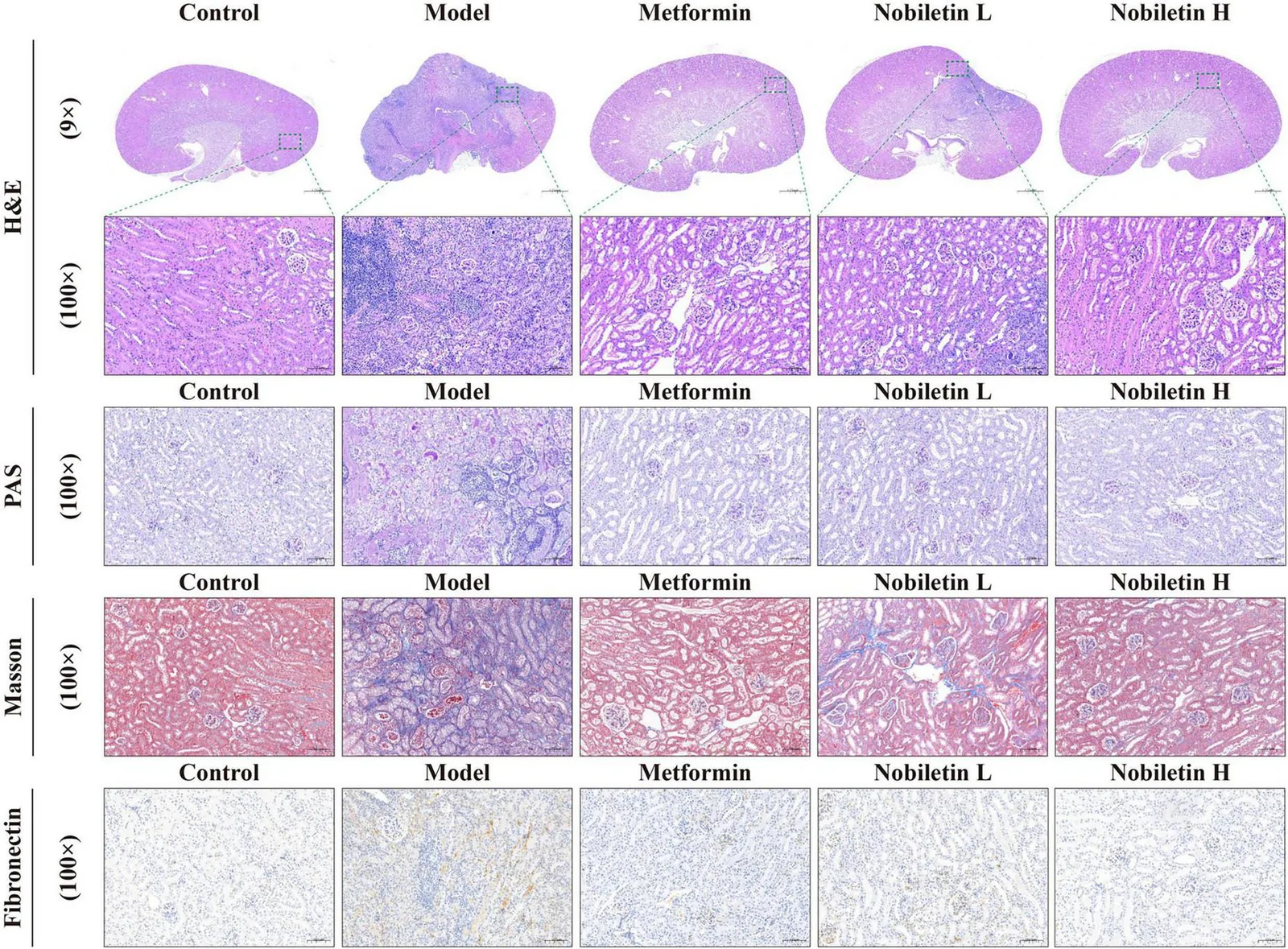
Histopathological and fibrotic changes in the kidneys of db/db mice and protective effects of nobiletin. Representative images of renal tissue stained with hematoxylin and eosin (H&E, 9 × and 100 ×), periodic acid-Schiff (PAS, 100 ×), Masson’s trichrome (100 ×), and fibronectin immunohistochemistry (100 ×) in the control, model, metformin, NOB-L, and NOB-H groups. H&E staining shows glomerular and tubular morphology; PAS staining highlights basement-membrane and mesangial-matrix changes; Masson’s trichrome detects collagen deposition (blue); and fibronectin staining shows extracellular-matrix accumulation. Scale bars are indicated in the images. Renal histology and fibronectin immunohistochemistry were performed in *n* = 3 independently processed animals per group, and representative images are shown. Slides and digital images were assigned coded identifiers before review, and the evaluator was not informed of treatment-group identity.

To evaluate the histological protective effects of NOB against diabetic nephropathy in db/db mice, hematoxylin and eosin (H&E), periodic acid-Schiff (PAS), Masson’s trichrome, and fibronectin (FN) staining were performed (Figure 5).

H&E staining revealed that the control group exhibited intact cortical and medullary structures, normal glomerular morphology, tightly arranged renal tubules, and histologically normal tubular epithelial cells. The model group showed severe pathological injury, including marked glomerular hypertrophy, expansion of the mesangial area, increased inflammatory cell infiltration, and degeneration/necrosis of tubular epithelial cells. Metformin treatment mitigated most pathological alterations, restoring glomerular and tubular morphology close to normal. The NOB-H group displayed clear improvement, with well-defined glomerular architecture, reduced inflammation, and nearly normal tubular morphology; NOB-L also alleviated some injuries but to a lesser extent.

PAS staining showed tightly organized glomerular structures and normal basement membrane thickness in controls. In the model group, the mesangial region appeared expanded, basement membranes appeared thickened, and glycoprotein deposition was increased, consistent with diabetic glomerular and basement-membrane injury. Metformin and NOB-H visibly attenuated these morphological changes, whereas NOB-L showed milder improvement.

Masson’s trichrome staining revealed minimal collagen deposition in controls, restricted mainly to vessel walls and occasional interstitial regions. The model group displayed prominent blue-stained collagen accumulation within glomerular and interstitial regions. Collagen deposition appeared reduced after metformin and NOB-H treatment, whereas NOB-L showed a smaller visual change.

Fibronectin immunostaining was low in controls and was mainly localized to the mesangial region and tubular basement membrane. The model group showed stronger and more extensive fibronectin staining in the glomerular mesangium and tubular interstitium. Metformin and NOB-H visibly reduced this staining pattern, while NOB-L showed a less pronounced reduction.

Collectively, the histological images showed glomerular hypertrophy, mesangial expansion, basement-membrane thickening, glycoprotein and collagen deposition, and increased fibronectin staining in diabetic db/db mice. These morphological abnormalities appeared attenuated after NOB treatment, particularly at the high dose. Because Figure 5 presents representative images rather than quantitative histomorphometric comparisons, these observations are interpreted qualitatively.

### 3.5 Metabolomic and energy metabolism modulation by nobiletin in the kidneys of db/db mice

To investigate the regulatory effects of NOB on the renal metabolic network in diabetic nephropathy, multidimensional statistical analysis and pathway enrichment were performed based on kidney tissue metabolomics data.

**FIGURE 6:**
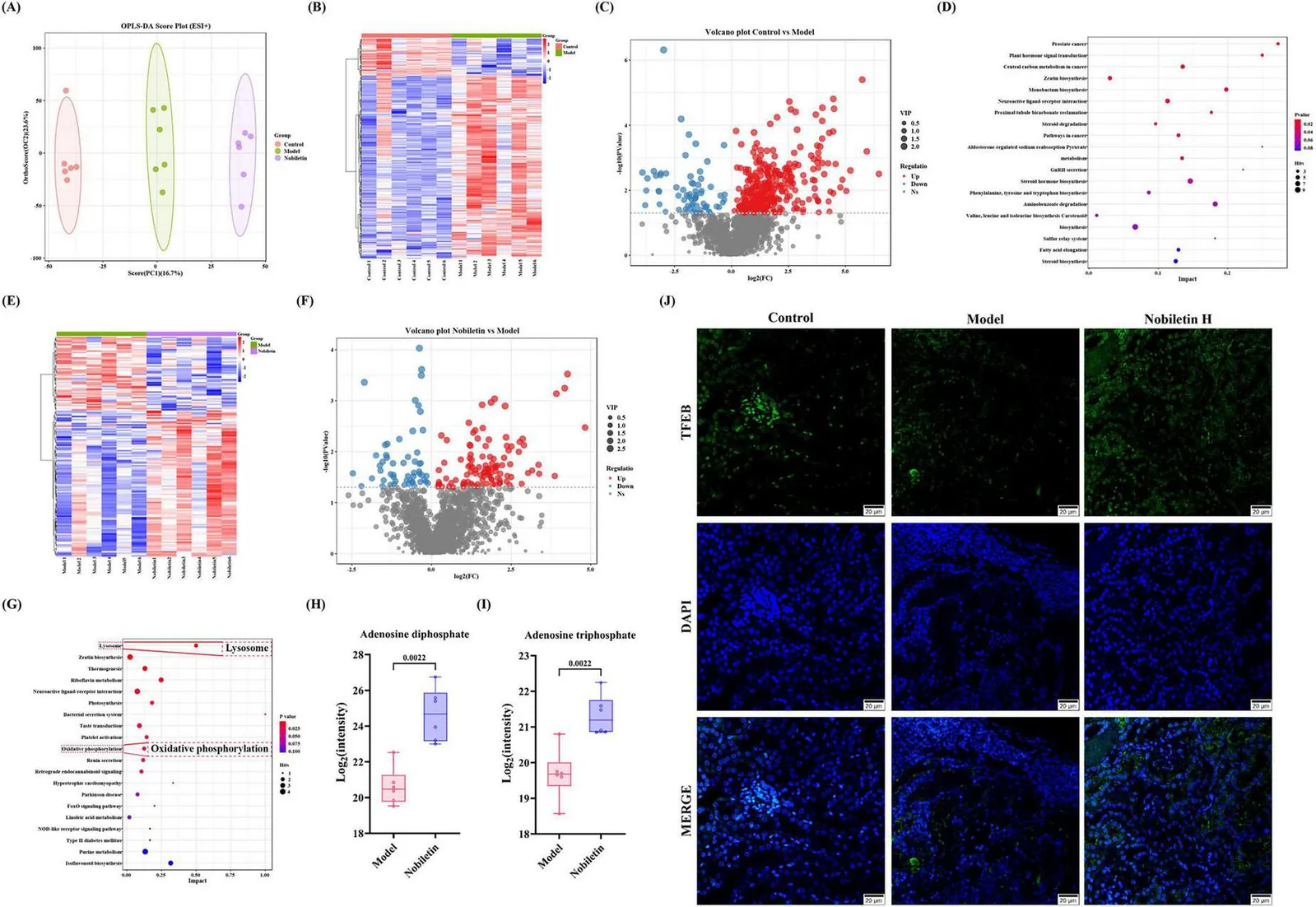
Nobiletin modulates renal metabolomic profiles, energy metabolism, and TFEB activation in db/db mice. **(A)** OPLS-DA score plot showing separation of metabolic profiles among Control, Model, and NOB-H groups; **(B,E)** heatmaps of FDR-controlled differential metabolites for Control vs Model and Model vs NOB-H comparisons; **(C,F)** volcano plots displaying increased and decreased differential metabolites, with plotted *P*-values representing FDR-adjusted *P-*values; **(D,G)** KEGG pathway enrichment illustrating diabetes- and NOB-affected metabolic pathways, including oxidative phosphorylation and lysosome-related function; **(H,I)** box plots comparing renal ADP and ATP levels between the Model and NOB-H groups; **(J)** immunofluorescence staining of TFEB (green), nuclei (DAPI, blue), and merged images in Control, Model, and NOB-H groups, showing increased TFEB expression and nuclear localization after NOB intervention (400 ×). The metabolomics results are interpreted as pathway-level evidence and require targeted metabolite validation in future studies. Untargeted metabolomics included *n* = 5 animals per group; ATP/ADP measurements included *n* = 6 animals per group; and TFEB immunofluorescence included *n* = 3 animals per group. Differential metabolites were screened using VIP > 1.0, FDR-adjusted *P* < 0.05, and FC > 1.0 or < 1.0. Exact *P*-values for the Model-versus-NOB-H ATP/ADP comparisons are displayed above the corresponding brackets.

Orthogonal partial least squares–discriminant analysis (OPLS-DA) score plots (Figure 6A) showed separation of renal metabolic profiles among the Control, Model, and NOB-H groups (*n* = 5 per group), indicating marked metabolomic variation in diabetes and its modulation by NOB treatment. Heatmap and volcano-plot analyses (Figures 6B,C,E,F) identified differential metabolites satisfying VIP > 1.0, FDR-adjusted *P* < 0.05, and directional FC criteria (FC > 1.0 for increased metabolites and FC < 1.0 for decreased metabolites).

KEGG pathway enrichment based on the FDR-controlled differential metabolites (Figures 6D,G) indicated changes involving oxidative phosphorylation, lysosomal function, fatty acid metabolism, the tricarboxylic acid cycle, and amino acid metabolism. NOB-H treatment significantly modulated oxidative phosphorylation- and lysosome-related pathways. Although multiple-testing correction was applied at the feature-selection stage, targeted metabolomics is still required to quantify representative metabolites and confirm specific metabolite-level mechanisms.

Measurement of energy-related molecules (Figures 6H,I) showed that NOB-H treatment significantly increased renal adenosine diphosphate (ADP) and adenosine triphosphate (ATP) levels relative to the Model group (*P* = 0.0022 for each comparison). These findings are consistent with partial restoration of renal energy status after NOB treatment.

Given the metabolomics data, the lysosomal and oxidative phosphorylation-regulating transcription factor EB (TFEB) was examined. Immunofluorescence (Figure 6J) demonstrated that NOB-H upregulated TFEB expression and promoted its nuclear localization, suggesting that NOB may improve mitochondrial energy generation, lysosomal function, and cellular recycling. However, because the untargeted metabolomics analysis was designed as pathway-level screening, targeted metabolomics is needed in future studies to identify and quantify the key metabolites driving these pathway changes.

### 3.6 Network pharmacology analysis and experimental validation of nobiletin targets in diabetic nephropathy

**FIGURE 7:**
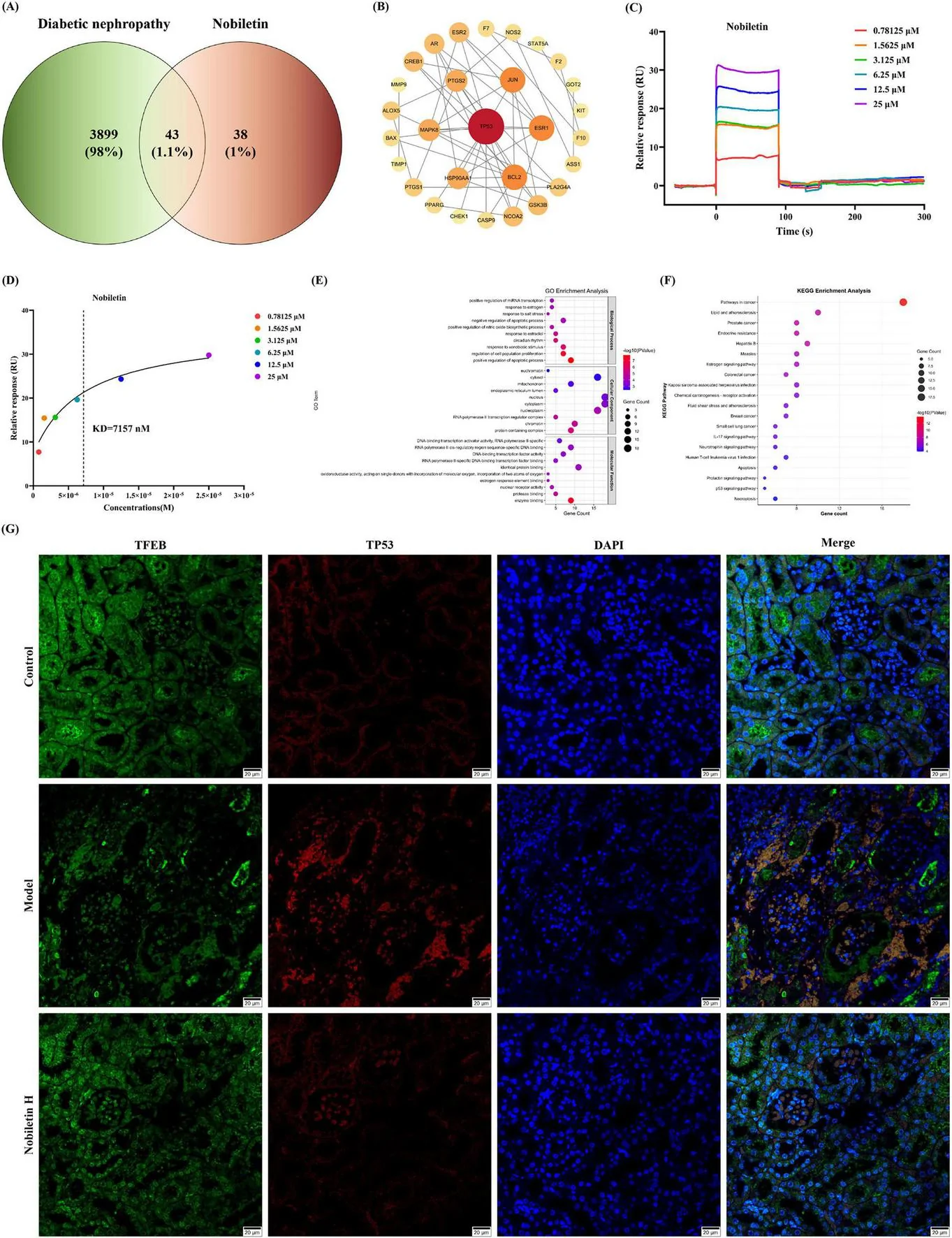
Network pharmacology analysis and validation of TP53 as a candidate target associated with the effect of nobiletin in diabetic nephropathy. **(A)** Venn diagram showing overlapping targets between diabetic nephropathy (DN) and NOB; **(B)** protein-protein interaction (PPI) network highlighting TP53 as a central hub; **(C,D)** surface plasmon resonance (SPR) sensorgrams and binding curves showing interaction between NOB and TP53 (KD = 7,157 nM); **(E)** Gene Ontology (GO) enrichment of overlapping targets; **(F)** Kyoto Encyclopedia of Genes and Genomes (KEGG) pathway enrichment analysis; **(G)** immunofluorescence staining of TFEB (green) and TP53 (red) in renal tissue from control, model, and NOB-H groups. Nuclei are stained with DAPI (blue). These data show that reduced TP53 signal after NOB treatment was accompanied by increased TFEB expression/nuclear localization, but they do not prove direct transcriptional regulation of TFEB by TP53 (400 ×). Renal TP53/TFEB immunofluorescence was performed in *n* = 3 animals per group. The KD of 7.157 μM indicates moderate micromolar affinity and supports a measurable interaction under the SPR assay conditions without establishing potent inhibition or *in vivo* target engagement.

Network pharmacology was applied to identify putative targets and enriched pathways of NOB in DN. Database mining yielded 3,899 DN-related targets and 81 NOB-related targets, of which 43 targets overlapped between DN and NOB (Figure 7A), suggesting that NOB may exert renoprotective effects through this shared target set.

Protein-protein interaction (PPI) network analysis (Figure 7B) revealed TP53 as a central hub, closely associated with metabolic regulation, cellular stress, and apoptosis-related pathways. Surface plasmon resonance (SPR) assays indicated binding between NOB and TP53 protein, with an equilibrium dissociation constant (KD) of 7,157 nM (Figures 7C,D), suggesting that TP53 may be a candidate target associated with the renal protective effect of NOB. The corresponding KD of 7.157 μM represents moderate micromolar affinity; therefore, the SPR result supports a measurable interaction under the assay conditions but does not by itself establish potent inhibition, intracellular target engagement, or exclusive TP53 dependence *in vivo*.

Gene Ontology (GO) enrichment (Figure 7E) showed that overlapping targets were significantly involved in oxidative stress response, metabolic regulation, mitochondrial function, apoptosis, and lysosome biogenesis. Kyoto Encyclopedia of Genes and Genomes (KEGG) pathway analysis (Figure 7F) identified key pathways for NOB action, including PI3K-Akt signaling, MAPK signaling, p53 signaling, lipid metabolism, and oxidative phosphorylation. These pathways were consistent with the metabolomics findings, particularly in relation to oxidative phosphorylation, lysosomal function, and energy metabolism deficits.

To integrate metabolomics and network pharmacology data, double immunofluorescence staining was performed to assess TP53 and TFEB signals in renal tissue (Figure 7G). Compared with the control group, diabetic kidneys showed increased TP53 signal and reduced TFEB expression/nuclear localization, suggesting enhanced cellular stress and impaired lysosomal regulatory status under diabetic conditions. NOB-H treatment reduced TP53 signal and was accompanied by increased TFEB expression and nuclear localization. These findings suggest that the renoprotective effect of NOB is associated with suppression of TP53 activation and restoration of TFEB-related lysosomal regulation. However, these data do not prove direct transcriptional regulation of TFEB by TP53.

### 3.7 Nobiletin attenuates pyroptosis, apoptosis, and necroptosis pathways in the kidneys of db/db mice

**FIGURE 8:**
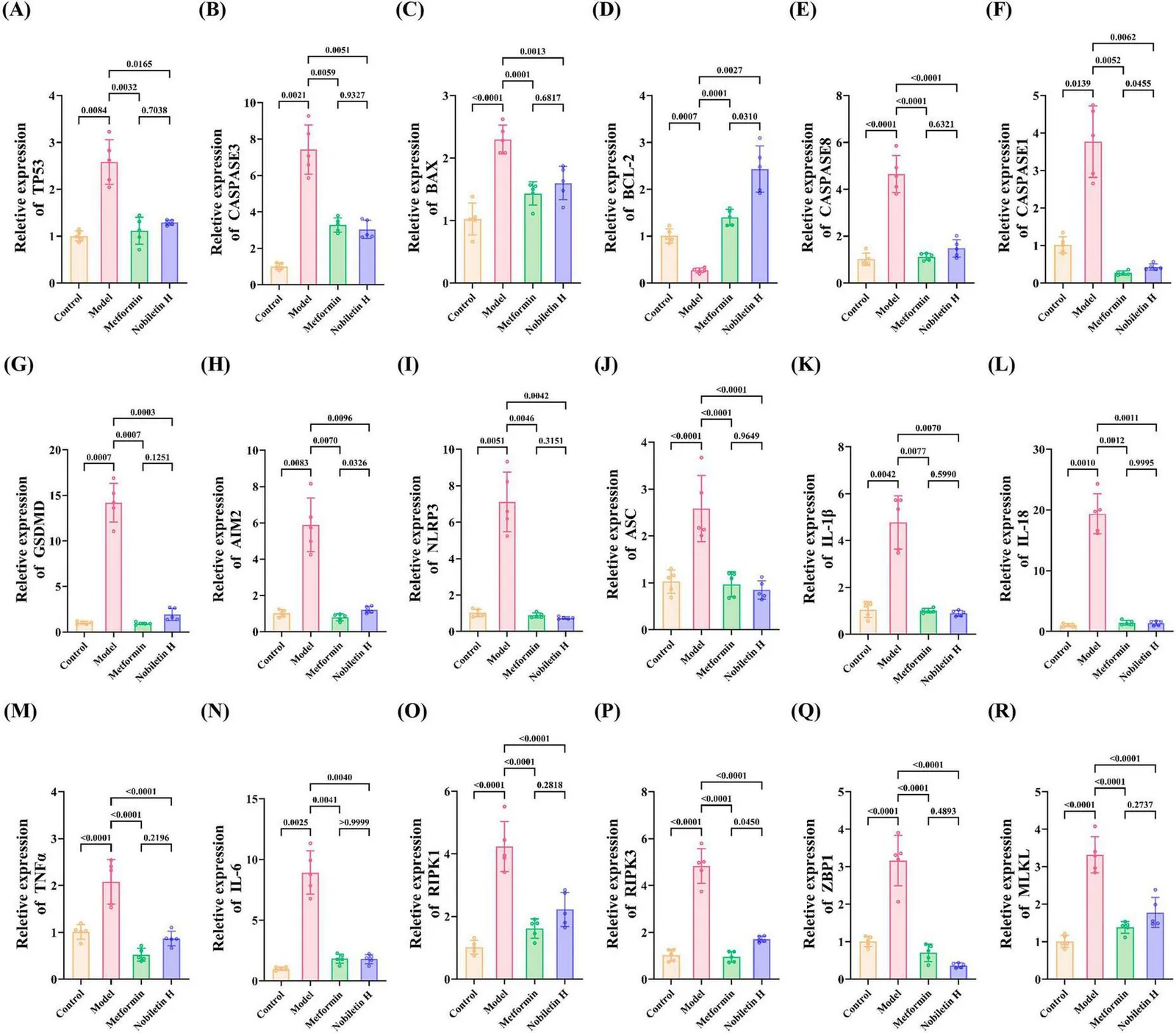
Nobiletin modulates pyroptosis-, apoptosis-, and necroptosis-related mRNA expression in the kidneys of db/db mice. **(A–F)** Apoptosis-related genes (TP53, CASPASE3, BAX, BCL-2, CASPASE8, and CASPASE9); **(G–L)** pyroptosis-related genes (GSDMD, AIM2, NLRP3, ASC, CASPASE1, and IL-1β); **(M–R)** necroptosis-related genes (TNF, IL-17, RIPK1, RIPK3, ZBP1, and MLKL). Data are presented as mean ± SEM (*n* = 5 per group), and statistical significance was determined by one-way ANOVA followed by Tukey’s *post-hoc* test or an appropriate alternative test (*P* < 0.05). Exact two-sided *P*-values are displayed above the corresponding comparison brackets, and each bracket identifies the groups compared.

Quantitative PCR analyses (Figures 8A–R) demonstrated that, compared with the control group, the kidneys of model mice exhibited significantly increased expression of pyroptosis-related genes NLRP3, CASPASE1, GSDMD, AIM2, ASC, and IL-1β (*P* < 0.01), indicating enhanced inflammatory programmed cell death in diabetic nephropathy. NOB treatment, particularly at high dose, markedly downregulated these genes (*P* < 0.05 or *P* < 0.01), restoring expression levels toward those in controls. For apoptosis-related genes, the model group showed elevated TP53, CASPASE3, CASPASE8, and BAX, along with reduced anti-apoptotic BCL-2 (*P* < 0.01). NOB intervention significantly suppressed pro-apoptotic gene expression and increased BCL-2 levels (*P* < 0.05–0.01), suggesting inhibition of apoptotic signaling in diabetic kidneys. Regarding necroptosis, mRNA levels of RIPK1, RIPK3, MLKL, and the upstream regulator ZBP1 were markedly elevated in the model group (*P* < 0.01). NOB treatment significantly reduced the expression of these necroptotic markers, indicating suppression of excessive necroptosis pathway activation. These results collectively indicate that NOB exerts renoprotective effects by simultaneously attenuating pyroptosis, apoptosis, and necroptosis at the transcriptional level.

**FIGURE 9:**
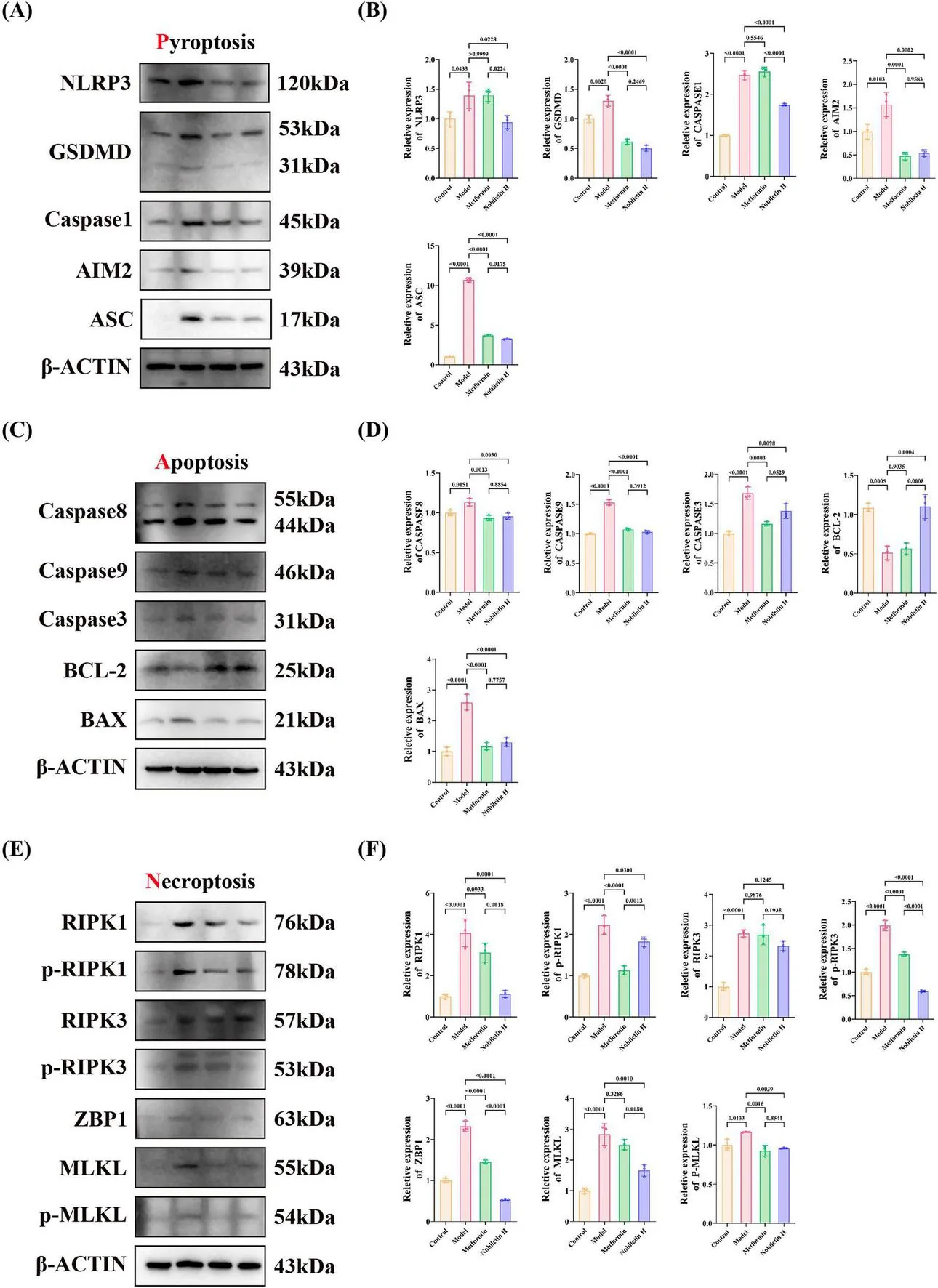
Western blot analysis of pyroptosis-, apoptosis-, and necroptosis-related proteins in the kidneys of db/db mice following Nobiletin treatment. **(A,B)** Pyroptosis-related proteins (NLRP3, cleaved GSDMD, Caspase-1, AIM2, and ASC); **(C,D)** apoptosis-related proteins (Caspase-8, Caspase-9, Caspase-3, BCL-2, and BAX); **(E,F)** necroptosis-related proteins (RIPK1, p-RIPK1, RIPK3, p-RIPK3, ZBP1, MLKL, and p-MLKL). Data are presented as mean ± SEM (*n* = 3 per group for Western blot analysis), and one-way ANOVA followed by Tukey’s *post-hoc* test or an appropriate alternative test was used (*P* < 0.05). Original uncropped renal Western blot images are provided as Supplementary Figure 4. Exact two-sided *P*-values are displayed above the corresponding comparison brackets, and each bracket identifies the groups compared.

Western blot analysis (Figures 9A–F) confirmed the qPCR findings. In the model group, protein levels of pyroptosis-related markers NLRP3, cleaved GSDMD, Caspase-1, AIM2, and ASC were markedly elevated compared with controls. NOB treatment significantly reduced the expression of these proteins, with high-dose NOB showing the most pronounced reduction. For apoptosis-related proteins, Caspase-8, Caspase-9, Caspase-3, and BAX were all increased in the model group, while anti-apoptotic BCL-2 was decreased. NOB intervention effectively reversed these changes—lowering pro-apoptotic protein levels and increasing BCL-2 expression. In the necroptosis pathway, the model group exhibited elevated levels of RIPK1, phosphorylated p-RIPK1, RIPK3, p-RIPK3, MLKL, p-MLKL, and upstream regulator ZBP1. NOB treatment significantly decreased the expression of these necroptosis-related proteins, again with a more marked effect observed in the high-dose group. These results provide protein-level confirmation that NOB mitigates the activation of pyroptosis, apoptosis, and necroptosis pathways in diabetic nephropathy.

Collectively, our findings indicate that in the setting of DN, the kidney exhibits concurrent activation of three forms of programmed cell death: inflammasome-dependent pyroptosis, mitochondria-associated apoptosis, and necroptosis. These pathological processes may interact and synergistically exacerbate renal injury. NOB mitigated this pathological cascade by downregulating the NLRP3-Caspase-1-GSDMD pyroptotic pathway, suppressing TP53-associated apoptotic signaling, and reducing activation of the RIPK1-RIPK3-MLKL necroptotic axis. These results suggest that NOB possesses multi-target, multi-pathway intervention potential for DN, but further functional validation is required to confirm the causal role of TP53.

### 3.8 Supplementary TP53 functional validation in HK2 cells

To further support the involvement of TP53 in PANoptosis-related protein regulation, we performed supplementary *in vitro* experiments using HK2 cells. Lentiviral transduction with GFP-positive TP53 knockdown and TP53 overexpression vectors showed successful cell transduction, and reduced TP53 expression in SH-TP53 cells and increased TP53 expression in OE-TP53 cells (Supplementary Figures 1, 3A). CCK-8 analysis was used to evaluate the effects of HG and NOB on HK2 cell viability and to guide subsequent treatment conditions (Supplementary Figure 2). Under HG conditions, expression patterns consistent with reductions in ASC, Caspase-1, NLRP3, Caspase-8, BAX, RIPK1, p-RIPK1, and ZBP1 after TP53 knockdown (Supplementary Figures 3B–H). Under NOB-treated conditions, TP53 overexpression showed expression patterns consistent with maintenance or elevation of NLRP3, Caspase-8, ZBP1, and p-RIPK1 relative to the corresponding overexpression control (Supplementary Figures 3I–K). TFEB expression was also examined under TP53 perturbation (Supplementary Figure 3L). These findings support the involvement of TP53 in NOB-associated regulation of PANoptosis-related proteins, while quantitative rescue studies are still needed to define causality and determine whether TP53 directly regulates TFEB transcription or lysosomal biogenesis.

## 4 Discussion

DN is a global public health issue, and specific therapeutic drugs have yet to be developed (26, 27). This highlights the need to explore natural medicines that may offer complementary strategies for DN intervention. NOB (28, 29), a flavonoid derived from citrus, is easily accessible and possesses the characteristics of both medicine and food (30). This study systematically evaluated the multi-target renoprotective effects of NOB against DN by integrating metabolomics, network pharmacology, and molecular biology approaches. Although NOB has been reported to ameliorate type 2 diabetes (31), its protective mechanisms against diabetes-induced renal injury remain unclear. Our results suggest that NOB attenuates DN progression partly in association with suppression of TP53-associated PANoptosis signaling and restoration of energy/lysosomal metabolic status.

In db/db mice, DN was characterized by significant disturbances in glucose and lipid metabolism, renal functional impairment, elevated inflammatory cytokines, and marked renal fibrosis, accompanied by disorders in energy metabolism and impaired lysosomal function, which is consistent with previous reports (32). At the molecular level, we observed concurrent activation of inflammasome-dependent pyroptosis, apoptosis, and necroptosis. This co-activation supports the emerging concept that multiple programmed cell death pathways interact synergistically to exacerbate chronic kidney injury (33). TP53 is a classic stress response transcription factor that can sense DNA damage, oxidative stress, and energy metabolism imbalance, thereby contributing to cellular injury and programmed cell death (34). NOB intervention, especially at high dose, markedly improved metabolic and functional parameters and suppressed the activation of these cell death pathways.

Metabolomics analysis suggested that oxidative phosphorylation and lysosome-related pathways were altered in the kidneys of DN mice, indicating that energy-metabolic abnormalities may contribute to DN progression (35, 36). In the direct Model-versus-NOB-H comparison, high-dose NOB increased renal ATP and ADP levels and promoted TFEB expression and nuclear localization. TFEB is an important regulator of lysosomal biogenesis and cellular recycling; therefore, restoration of TFEB-related lysosomal function may facilitate clearance of damaged organelles and abnormal protein aggregates while reducing excessive inflammasome activation and programmed cell death (37, 38).

Network pharmacology is a research approach that constructs a relationship network among active ingredients, targets, signaling pathways, and diseases (39). In our analysis, TP53 emerged as a core intersecting target between NOB and DN. SPR assays indicated a binding interaction between NOB and TP53, with a KD value of 7,157 nM. Immunofluorescence revealed a marked upregulation of renal TP53 in DN, which was reduced by NOB treatment, accompanied by restoration of TFEB expression and nuclear localization. Subsequent qPCR and Western blot analyses indicated that NOB downregulated multiple PANoptosis-related molecules, including BAX, Caspase-8, RIPK1, and NLRP3, suggesting broad inhibition of pyroptotic, apoptotic, and necroptotic pathways. This prioritization was supported by recent evidence that p53 coordinates both apoptotic and non-apoptotic cell-death programs and can participate in chronic inflammation and PANoptosis-associated responses (18, 19). Nevertheless, TP53 was interpreted as a candidate integrative stress-response hub rather than the only PANoptosis regulator.

The SPR-derived KD of 7.157 μM falls within the micromolar range commonly measurable for small-molecule–protein interactions (40). In the present context, this value indicates moderate affinity and supports TP53 as a candidate NOB-interacting protein, but it should not be interpreted as evidence of high-potency pharmacological inhibition or *in vivo* target engagement.

These findings indicate that apoptosis, pyroptosis, and necroptosis in DN are not independent events but may be synergistically activated, consistent with PANoptosis (41, 42). NOB was associated with reduced TP53 activation, attenuation of PANoptosis-related pathways, and restoration of energy metabolism and lysosomal regulatory status. The supplementary HK2 experiments provided representative expression patterns consistent with reduced HG-associated ASC, Caspase-1, NLRP3, Caspase-8, BAX, RIPK1, p-RIPK1, and ZBP1 after TP53 knockdown, whereas TP53 overexpression showed patterns that partially opposed the NOB-associated changes in NLRP3, Caspase-8, ZBP1, and p-RIPK1. However, these representative data do not establish direct transcriptional regulation of TFEB by TP53 or prove that the renoprotective effect of NOB is exclusively dependent on TP53. Therefore, quantitative rescue experiments, TP53 conditional knockout models, TFEB promoter luciferase assays, ChIP-qPCR, and targeted metabolomics are still needed.

Recent studies on Wogonoside in DN further emphasize the importance of podocyte injury, tubular epithelial oxidative damage, and inflammatory signaling in disease progression. Li et al. reported that Wogonoside attenuated podocyte injury in DN by targeting the NF-κB p65-MMP28 axis (43). Another study by Li et al. showed that Wogonoside ameliorated oxidative damage in tubular epithelial cells of DN by modulating the HNF4A-NRF2 axis (44). In line with these reports, our findings suggest that a citrus-derived polymethoxyflavone may exert renoprotective effects through multi-target regulation of metabolic stress, oxidative stress, inflammation, podocyte/tubular injury, and programmed cell-death-related pathways.

Recent publications also illustrate broader methodological and translational directions relevant to future natural-product research. Integrated computational, transcriptomic, and *in vitro* strategies have been applied to characterize bioactive plant compounds and epigenetic regulators (45–49), whereas machine-learning and precision-nutrition approaches have been used to optimize functional-food development and individualized dietary intervention (50, 51). In parallel, biocompatible nanoscaffolds, advanced nanoparticles, extracellular-vesicle hybrids, and other materials-based delivery platforms are being developed to improve stability, targeting, and translational performance (52–56). Work on cellular senescence further highlights the intersection of metabolic stress, neuroendocrine signaling, and epigenetic regulation (57). These studies do not directly establish NOB efficacy in DN; rather, they identify analytical, formulation, and delivery strategies that may inform future translational development of NOB.

The mTOR-TFEB-lysosome/autophagy axis may also be relevant to the protective effect of NOB. mTORC1 integrates nutrient signals with β-cell function and metabolic disease through phosphorylation-dependent regulatory programs (58), while nuclear mTOR signaling can coordinate transcriptional programs underlying cellular growth and metabolism (59). Amino-acid sensing through mTOR also contributes to systemic glucose regulation (60). More broadly, mTOR-related metabolic and stress-response pathways are implicated in muscle aging and disease (61), and nutrient-sensing mechanisms contribute to the lifespan and healthspan effects of dietary restriction across species (62). Under diabetic conditions, persistent nutrient overload and metabolic stress may disturb mTOR-TFEB signaling, impair lysosomal homeostasis, and amplify inflammatory cell death. In the present study, NOB improved renal energy-related measures, increased TFEB expression/nuclear localization, and reduced PANoptosis-related markers. Although mTOR pathway proteins were not directly measured, these observations support future investigation of whether mTOR-TFEB-dependent lysosomal regulation links TP53-associated metabolic stress with PANoptosis in DN.

This study has several limitations. First, although db/db mice are widely used as a model of type 2 diabetes-associated renal injury, they do not fully reproduce all pathological features of human DN. Second, the supplementary HK2 cell experiments support the involvement of TP53 in HG-induced PANoptosis-related protein activation and in the regulatory effect of NOB; however, these data do not fully establish that the renoprotective effect of NOB is exclusively dependent on TP53. Third, although TP53 and TFEB showed opposite changes after NOB treatment *in vivo* and TFEB expression changed under TP53 perturbation *in vitro*, the present study does not prove direct transcriptional regulation of TFEB by TP53. Future studies using TFEB promoter luciferase assays, ChIP-qPCR, TP53 rescue experiments, and conditional TP53 knockout models are needed. Fourth, the untargeted metabolomics analysis provided pathway-level evidence for changes in oxidative phosphorylation, lysosomal function, fatty acid metabolism, and energy metabolism, but targeted metabolomics is required to identify and quantify the key metabolites driving these pathway changes. Finally, additional renal cell types, including podocytes and mesangial cells, will be included in future studies to further clarify the cell-type-specific mechanism of NOB in DN. In addition, only male db/db mice were studied. Sex-dependent differences in glucose metabolism, renal inflammation, fibrosis, and programmed-cell-death susceptibility may influence both DN progression and the response to NOB; therefore, the present findings should not be generalized to females without direct testing. Future studies should include female animals and formally evaluate sex-by-treatment interactions. Network pharmacology is also affected by database coverage, update frequency, prediction algorithms, target annotation, and publication bias, so database-derived targets should be regarded as candidates requiring experimental validation. Finally, all metabolomics samples were acquired in a single batch and no inter-batch correction was required. Feature-level *P*-values were adjusted to control the FDR; nevertheless, untargeted metabolite annotation and pathway enrichment still require targeted metabolomics for quantitative confirmation.

In conclusion, NOB ameliorated metabolic disturbance, renal dysfunction, inflammation, histological injury, and fibrosis in db/db mice. Mechanistically, these protective effects were associated with suppression of TP53 activation, restoration of TFEB-related lysosomal and energy metabolic status, and inhibition of PANoptosis-related pyroptotic, apoptotic, and necroptotic pathways. Supplementary HK2 cell experiments further supported the involvement of TP53 in HG-induced PANoptosis-related protein activation and in the regulatory effect of NOB. These findings suggest that NOB may be a promising natural compound for DN intervention and provide a basis for future studies targeting TP53-associated PANoptosis and metabolic stress in diabetic kidney disease.

## Data Availability

The raw data supporting the conclusions of this article will be made available by the authors, without undue reservation.
