# Supplementary material for: Nobiletin ameliorates diabetic nephropathy by modulating TP53-associated PANoptosis: an experimental study
Source: Front Nutr. 2026 Sep 4;13:1816623. doi: 10.3389/fnut.2026.1816623 (PMC13585718; doi:10.3389/fnut.2026.1816623)
Supplement: Supplementary file 1 [file Data_Sheet_1.zip › Data Sheet 1/Supplementary Figure S1-S3.PDF]

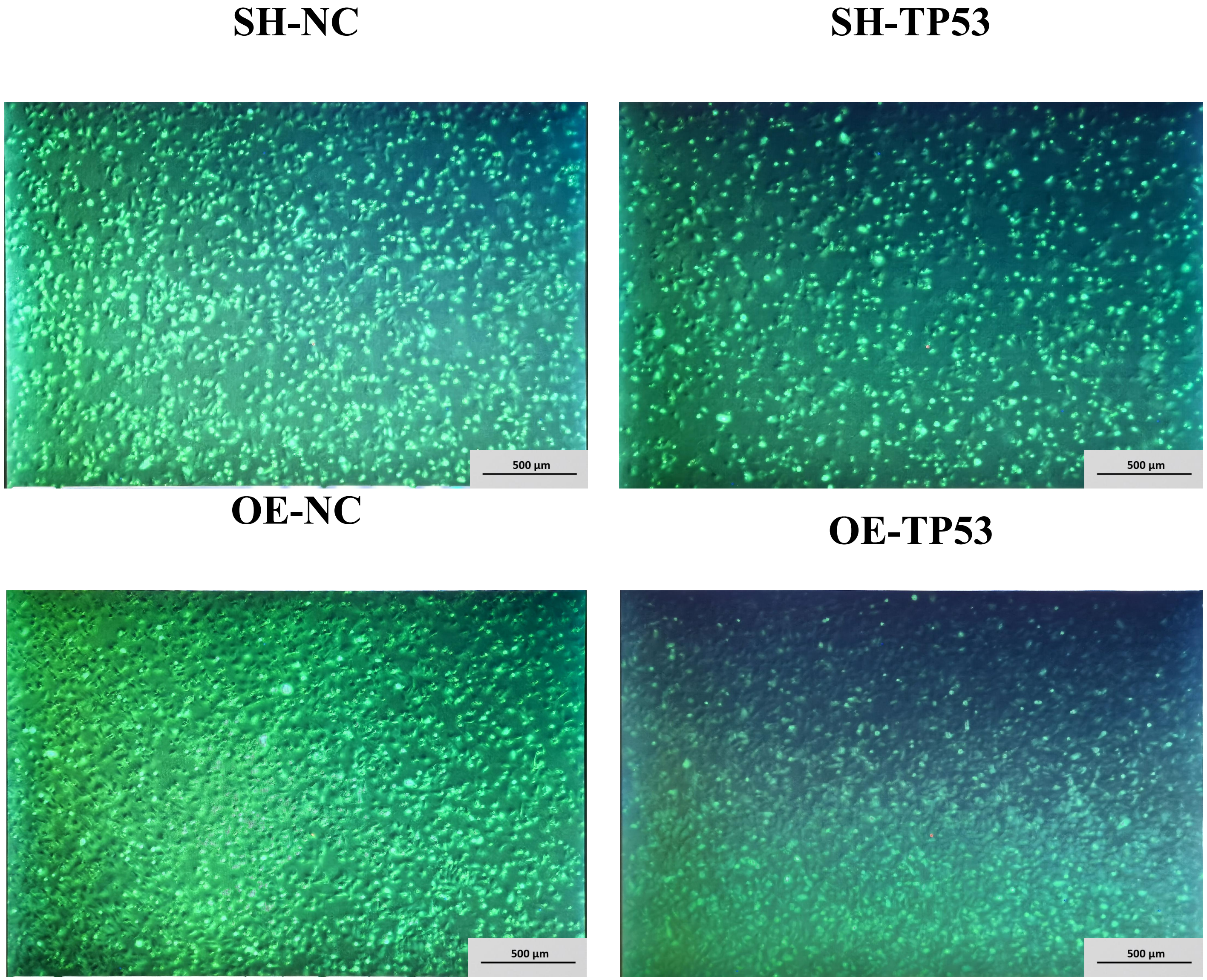

Figure S1. HK2 cells were transduced with lentiviruses containing the GFP gene and a puromycin resistance cassette to achieve TP53 knockdown and overexpression.

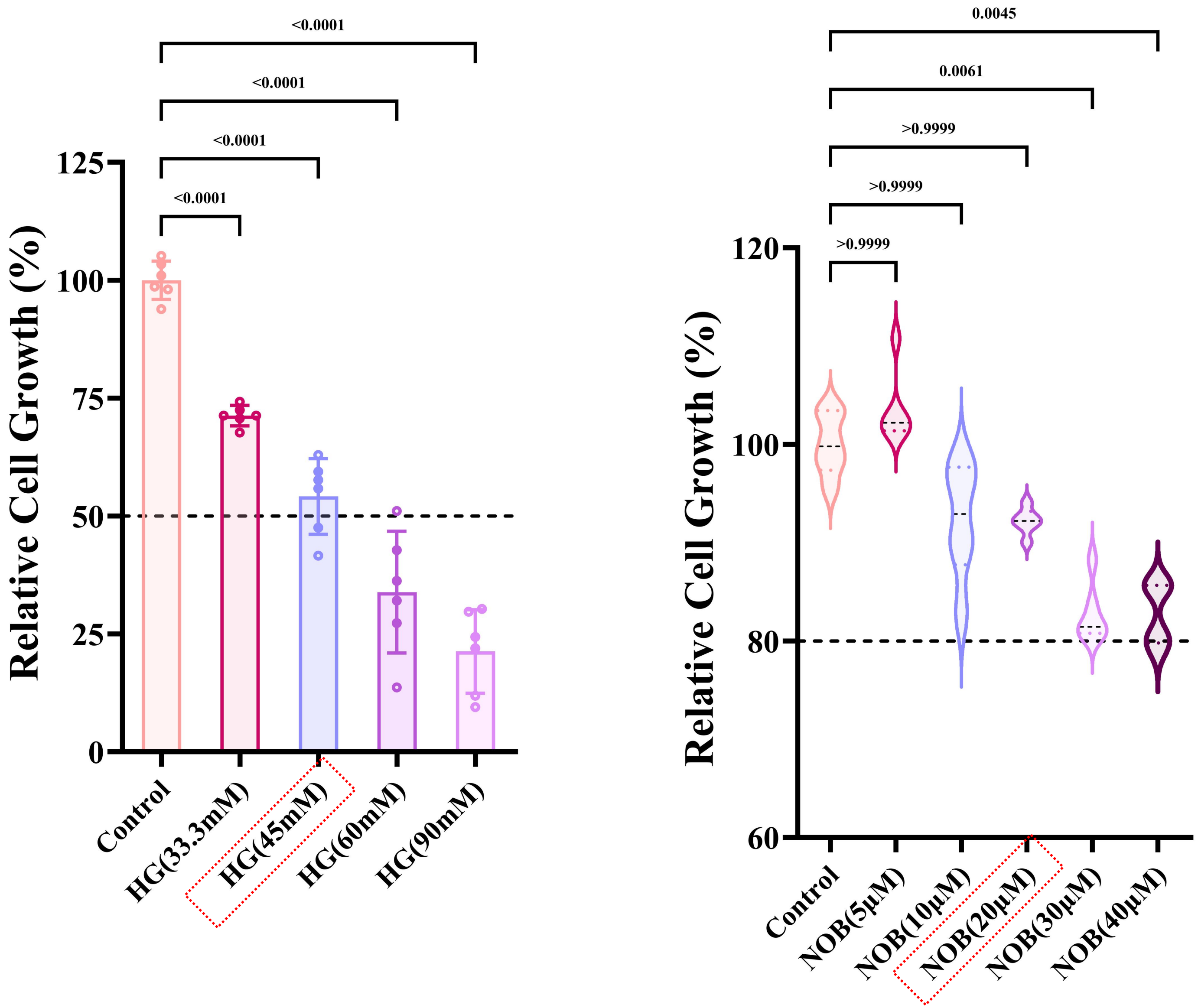

Figure S2.The effects of high glucose (HG) and NOB on the viability of HK2 cells were evaluated using the CCK-8 assay to inform subsequent experiments.

TP53

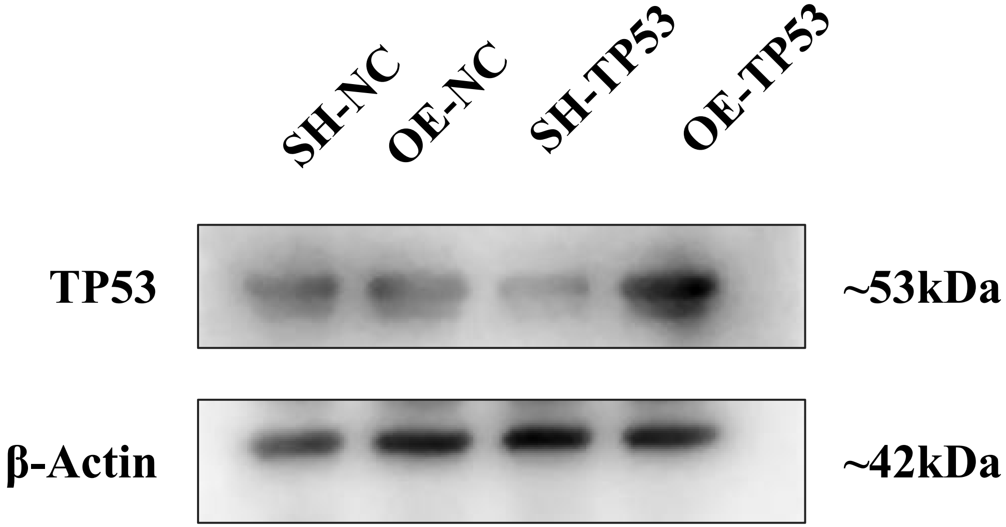

TP53

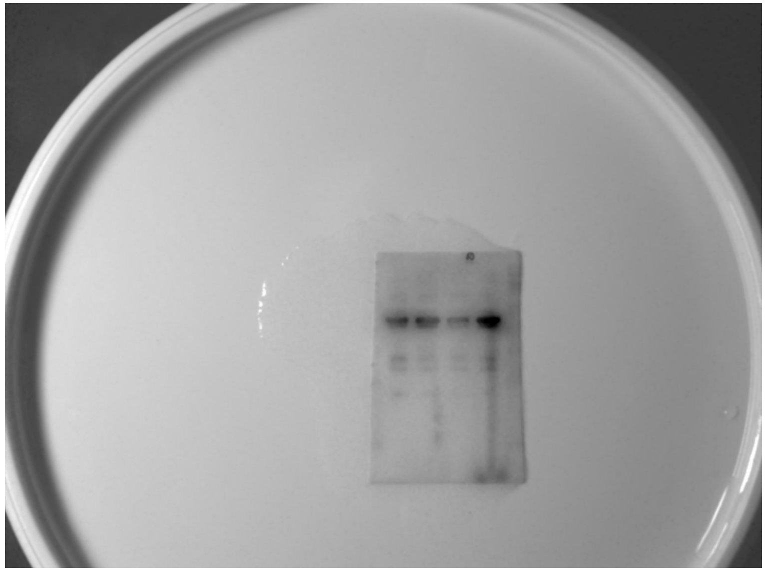

β-Actin

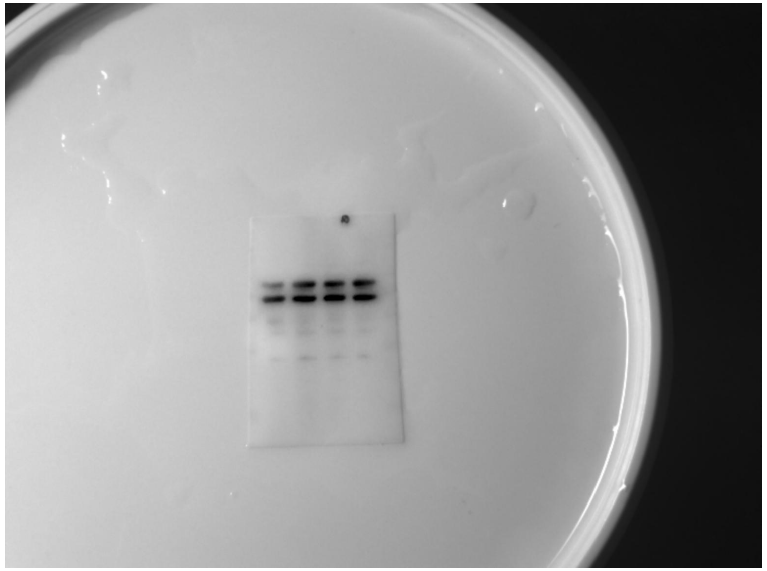

Figure S3A.The protein expression levels of TP53 in each group were detected using Western blotting.

ASC

TP53 knockdown

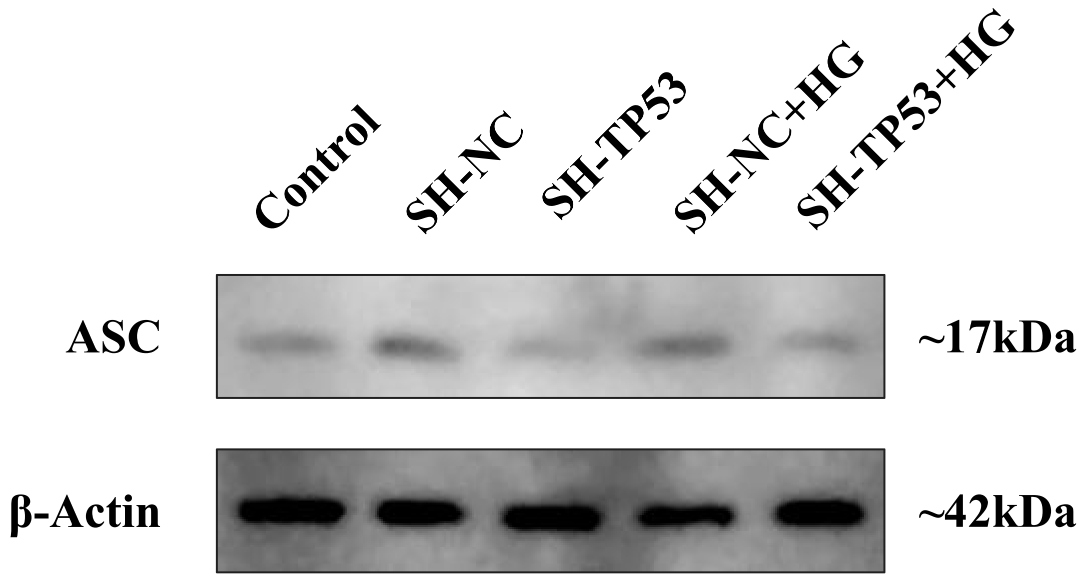

ASC

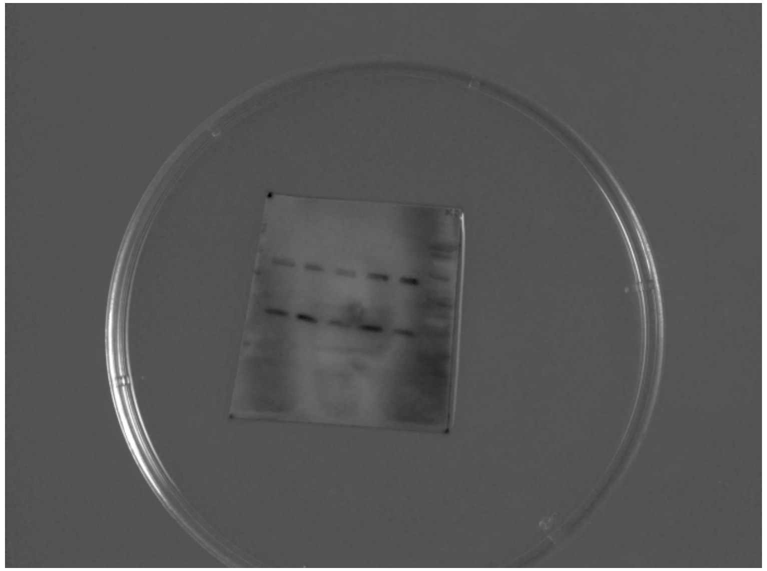

β-Actin

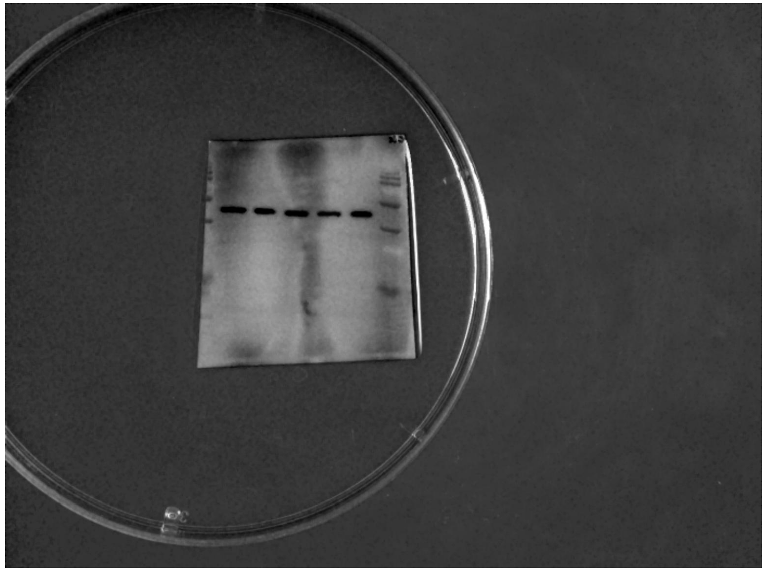

Figure S3B.The protein expression levels of ASC in each group were detected using Western blotting.

CASPASE1

TP53 knockdown

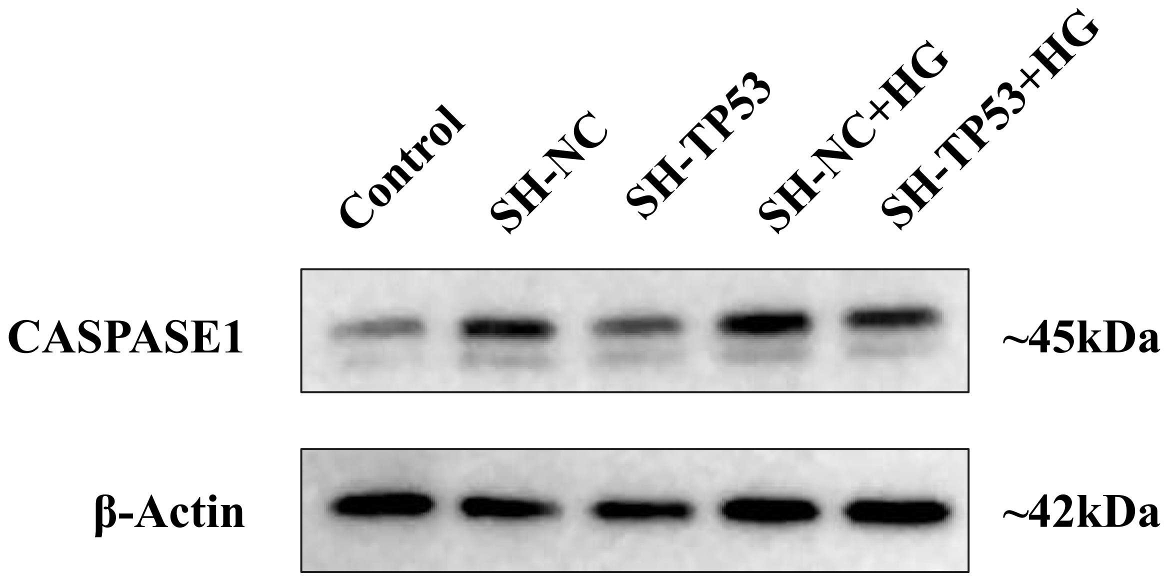

CASPASE1

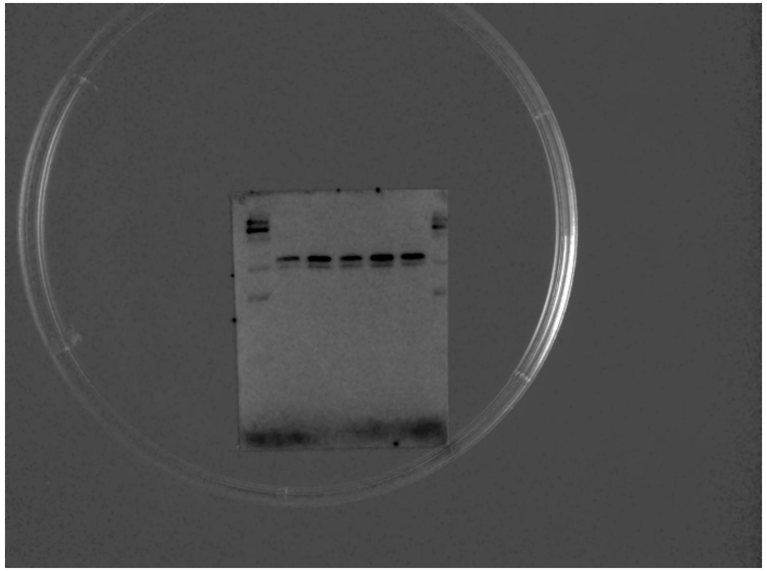

β-Actin

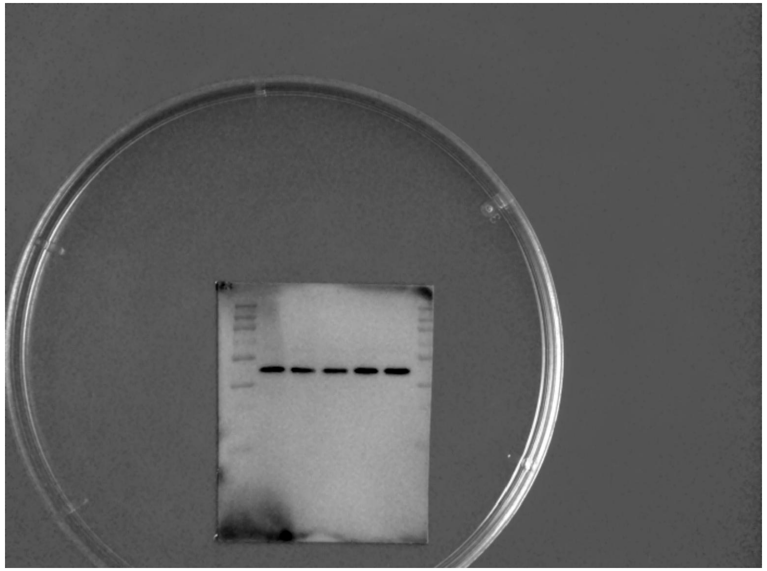

Figure S3C.The protein expression levels of CASPASE1 in each group were detected using Western blotting.

CASPASE8

TP53 knockdown

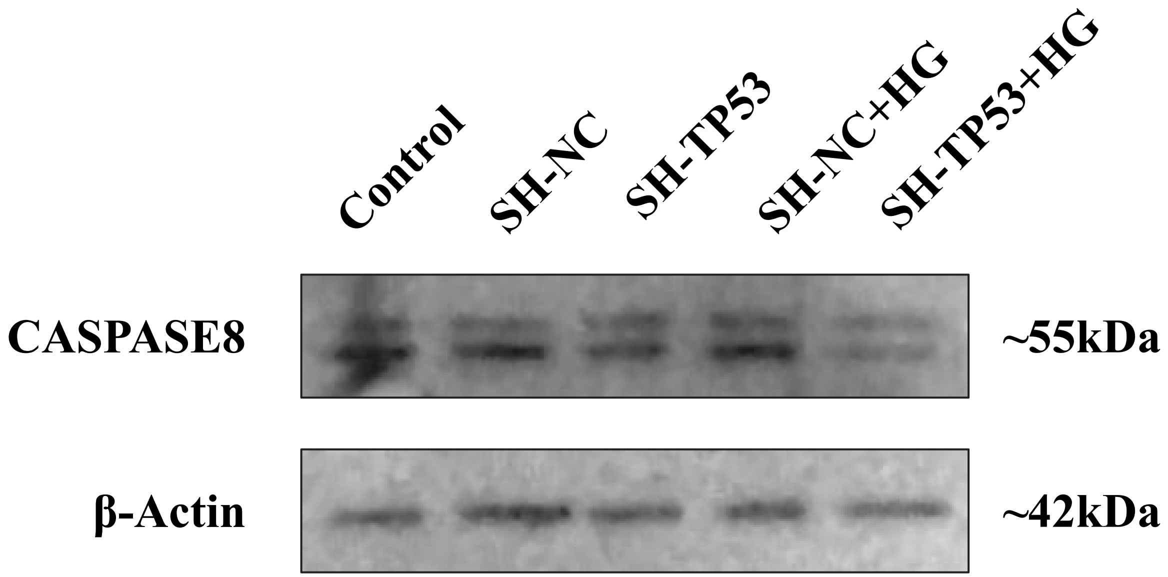

CASPASE8

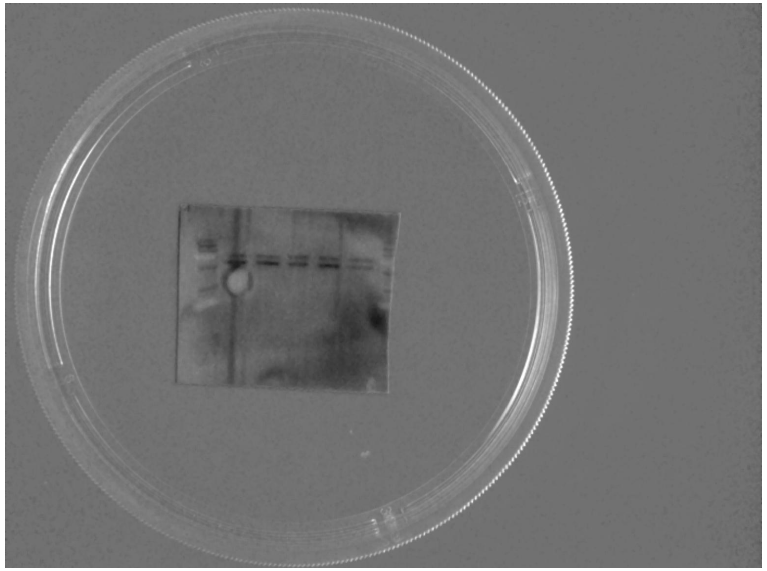

β-Actin

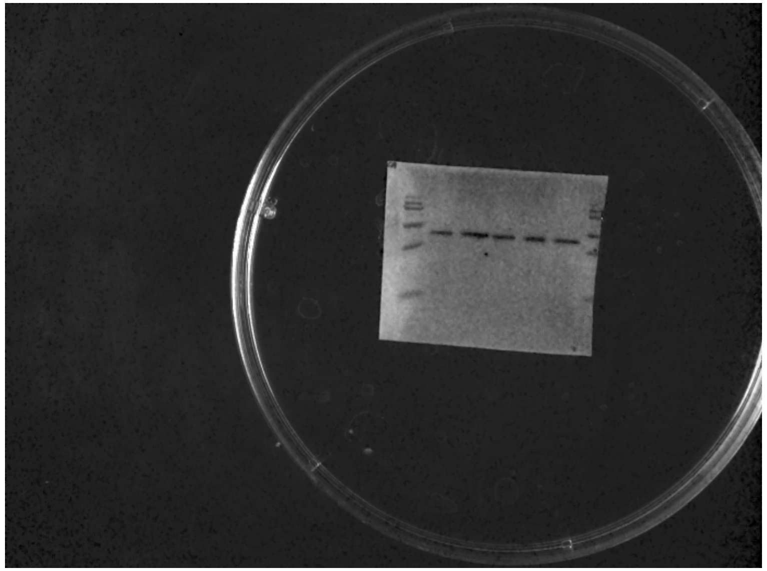

Figure S3D.The protein expression levels of CASPASE8 in each group were detected using Western blotting.

NLRP3+BAX

TP53 knockdown

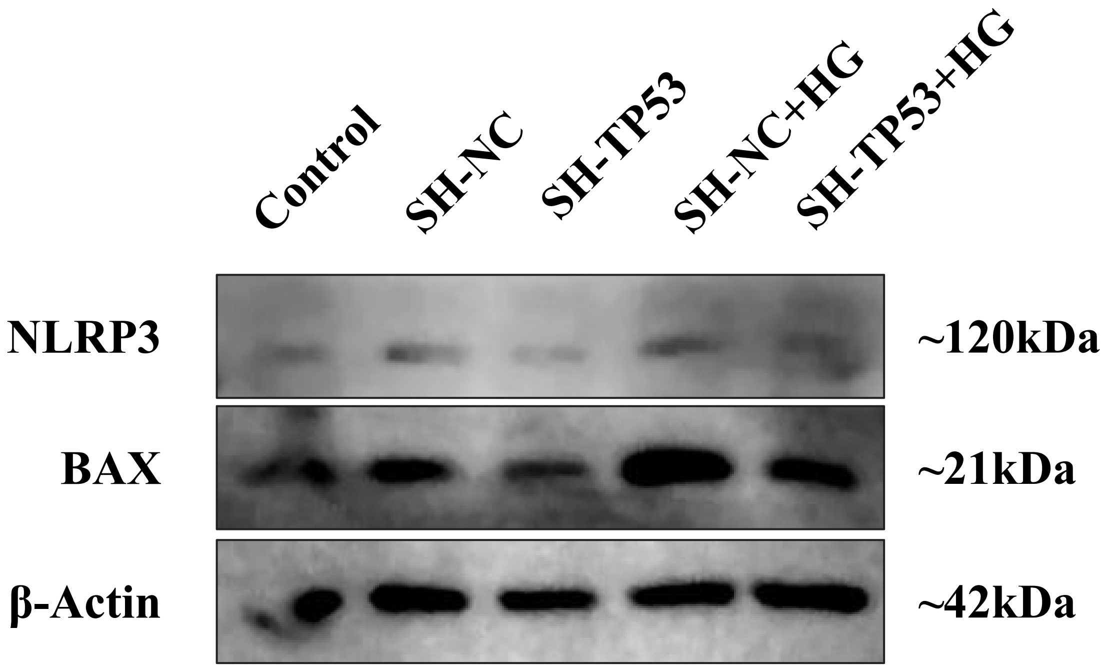

BAX

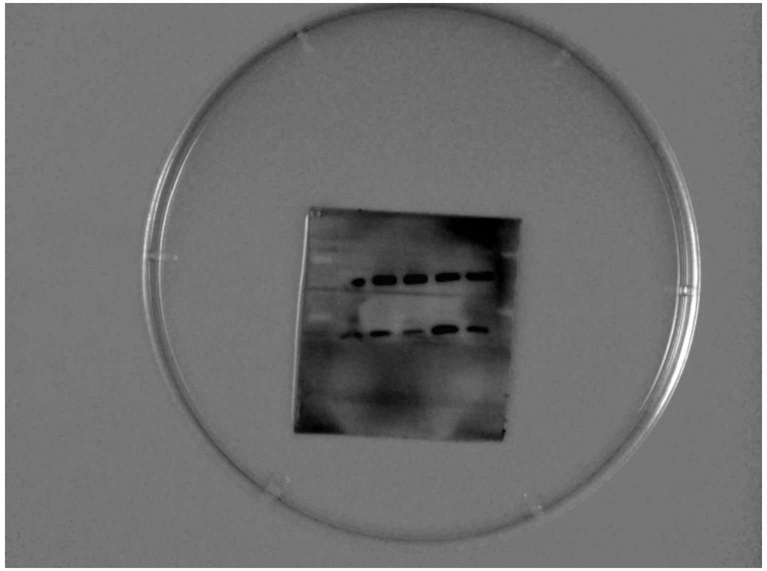

NLRP3

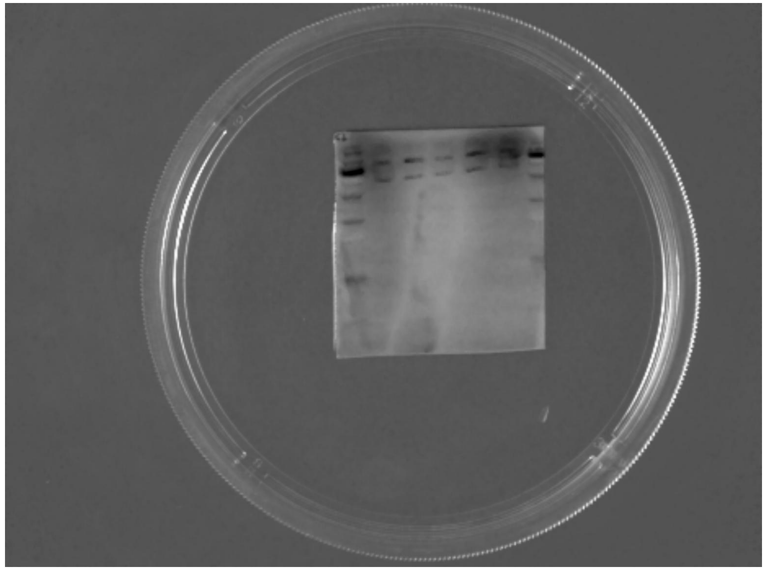

β-Actin

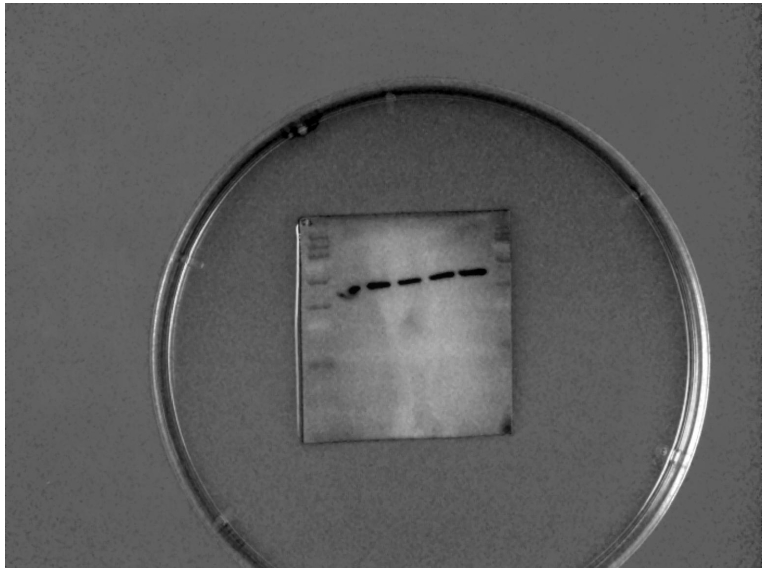

Figure S3E.The protein expression levels of NLRP3 and BAX in each group were detected using Western blotting.

RIPK1

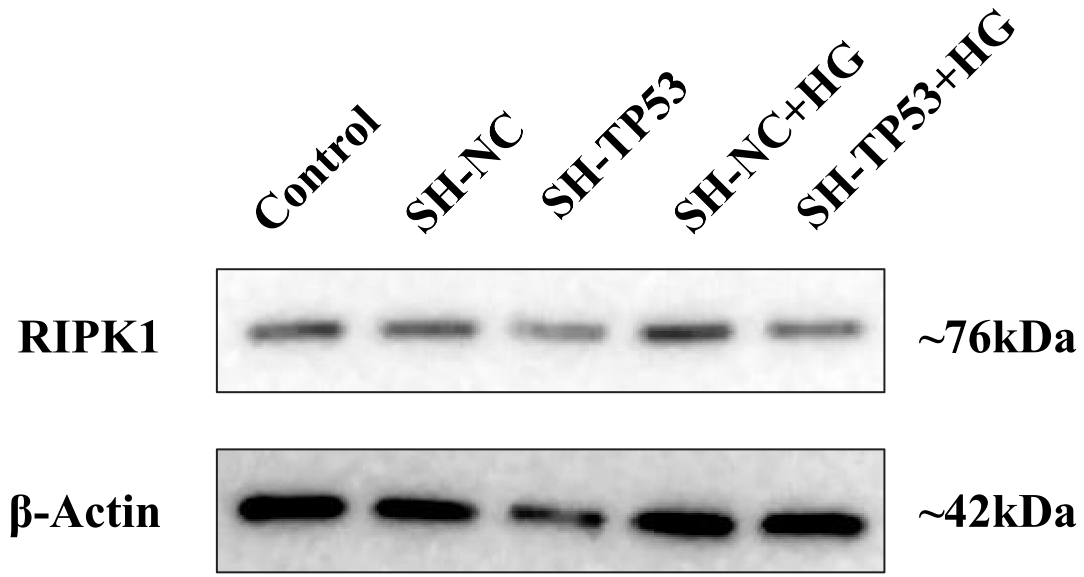

Figure S3F.The protein expression levels of RIPK1 in each group were detected using Western blotting.

TP53 knockdown

RIPK1

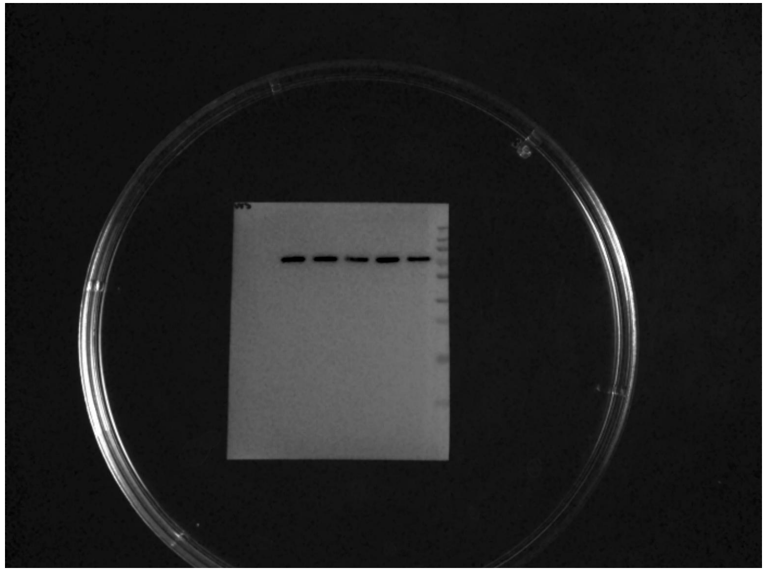

β-Actin

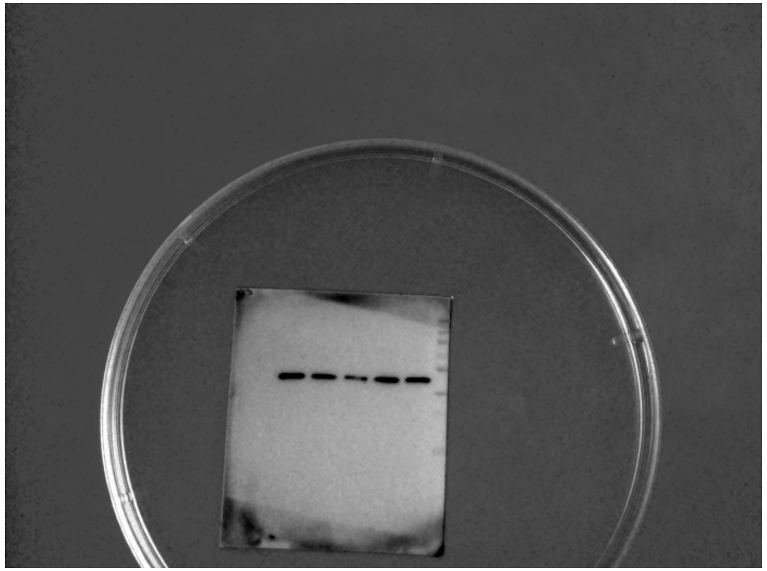

P-RIPK1

TP53 knockdown

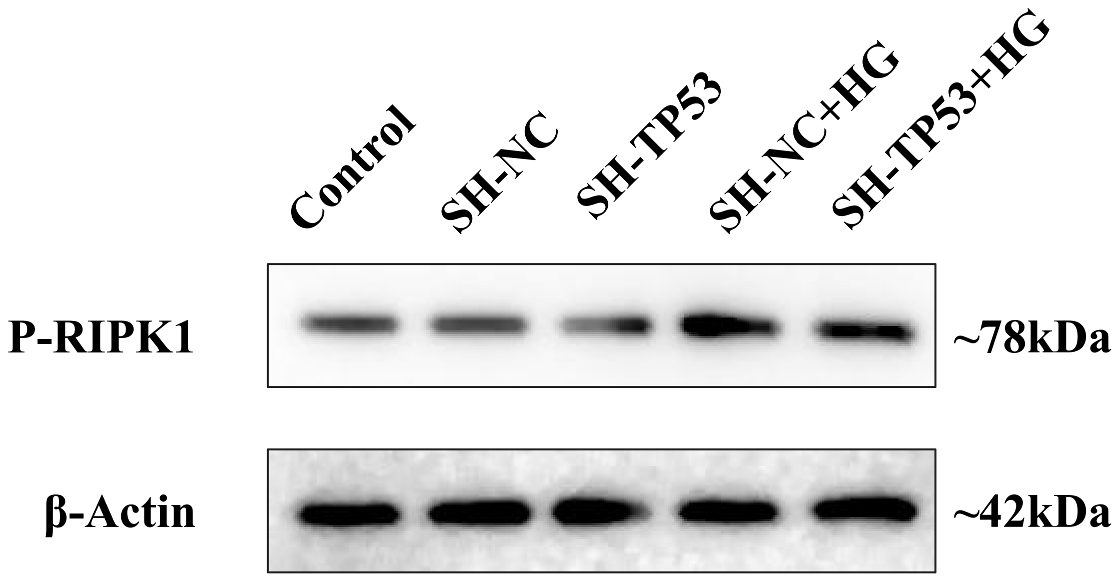

P-RIPK1

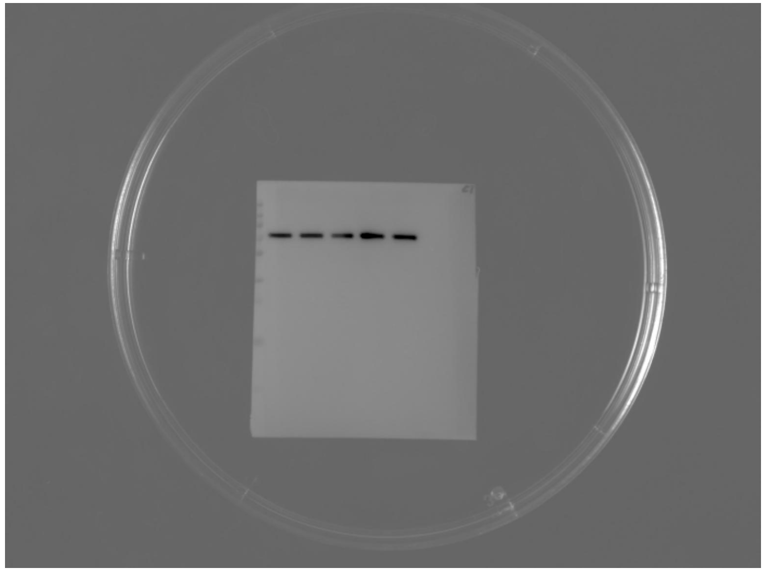

β-Actin

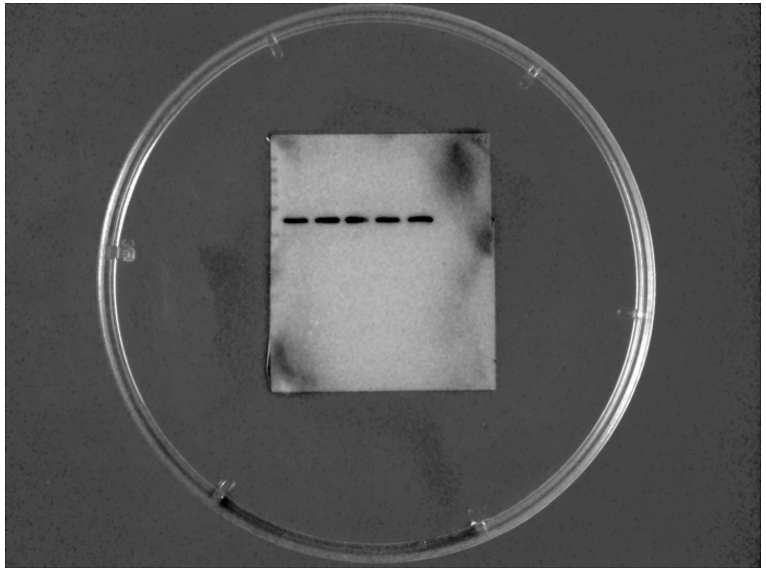

Figure S3G.The protein expression levels of p-RIPK1 in each group were detected using Western blotting.

ZBP1

TP53 knockdown

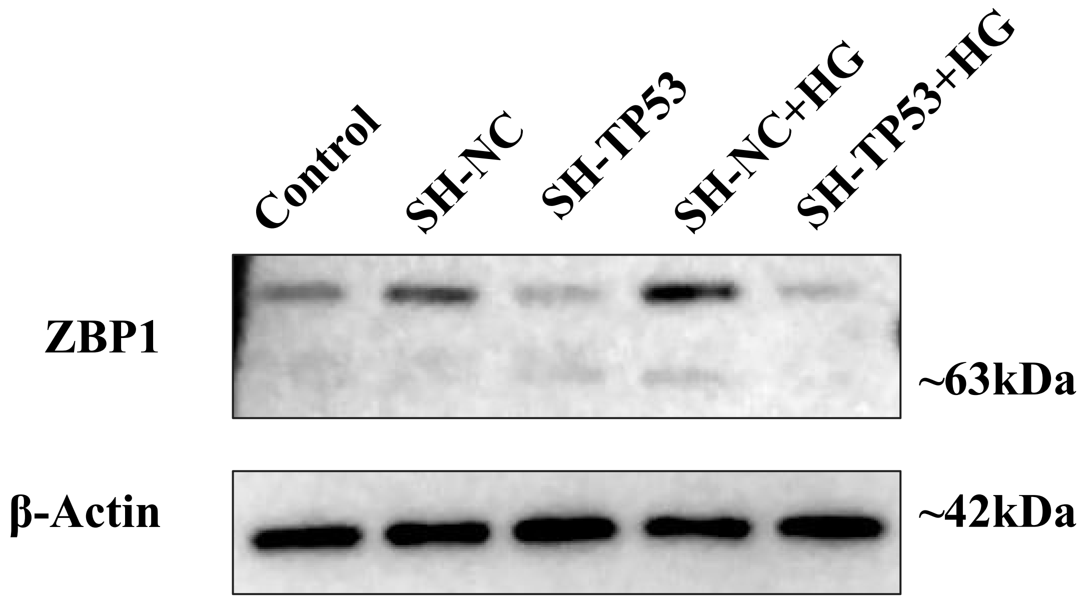

ZBP1

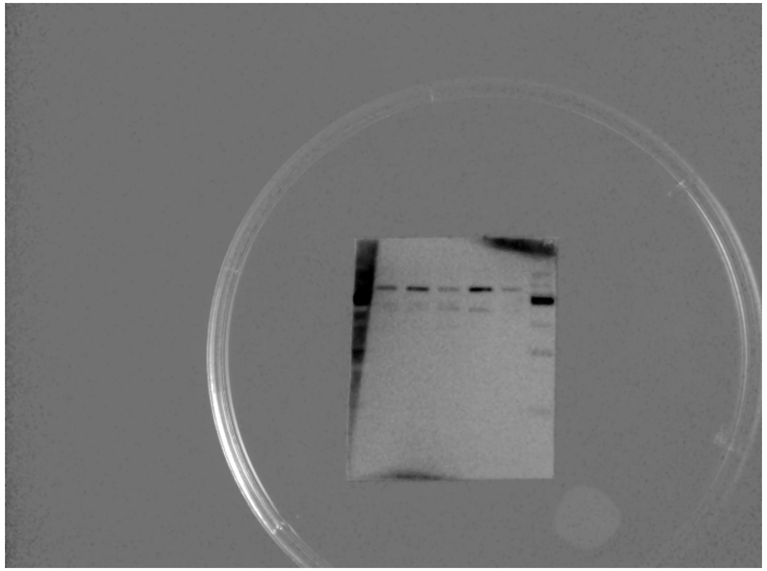

β-Actin

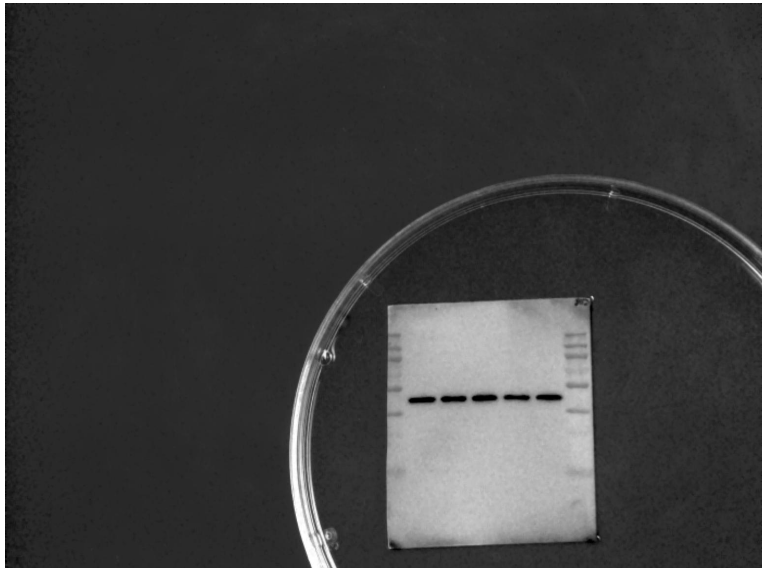

Figure S3H.The protein expression levels of ZBP1 in each group were detected using Western blotting.

P-RIPK1+ZBP1

TP53 overexpression

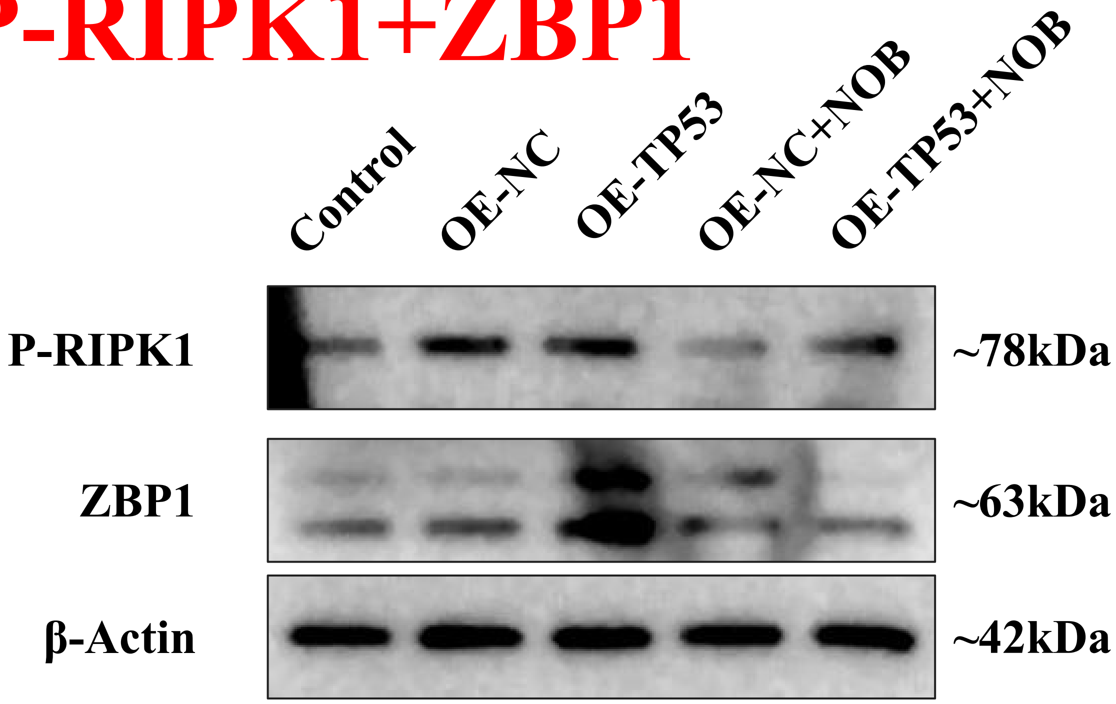

P-RIPK1

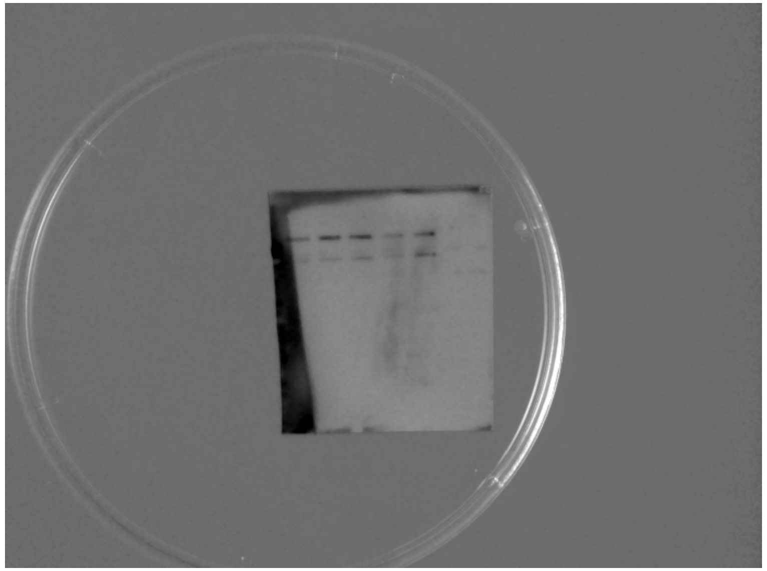

ZBP1

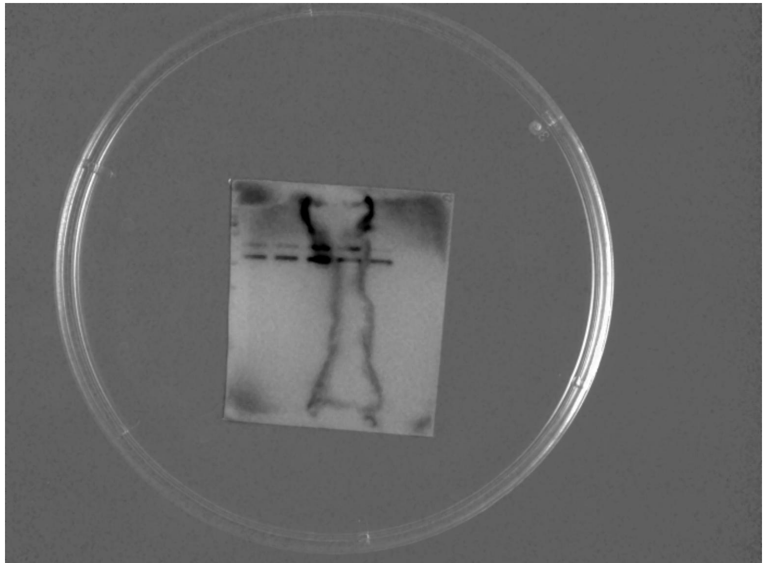

β-Actin

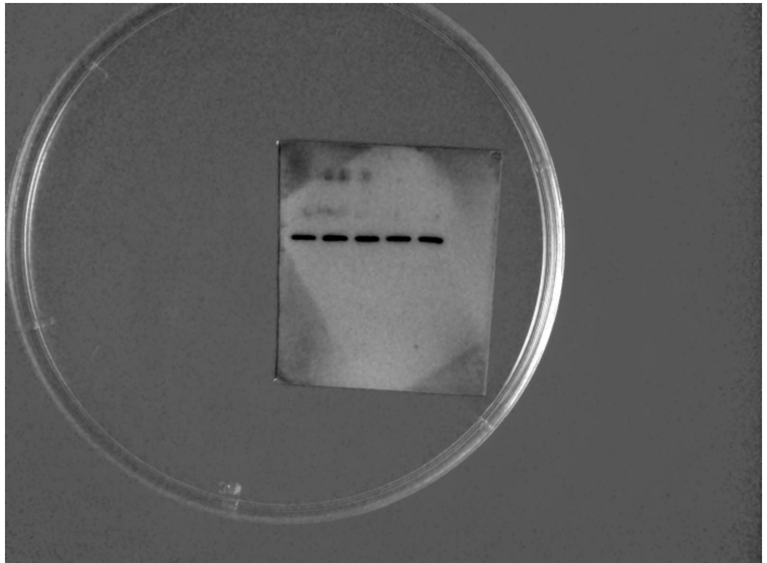

Figure S3I.The protein expression levels of ZBP1 and p-RIPK1 in each group were detected using Western blotting.

NLRP3

TP53 overexpression

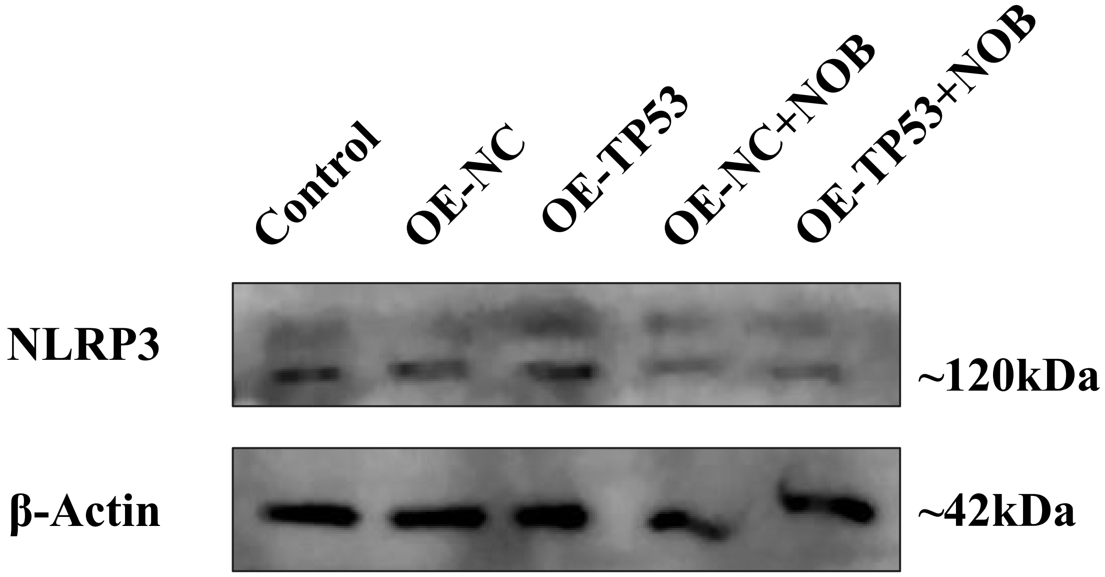

NLRP3

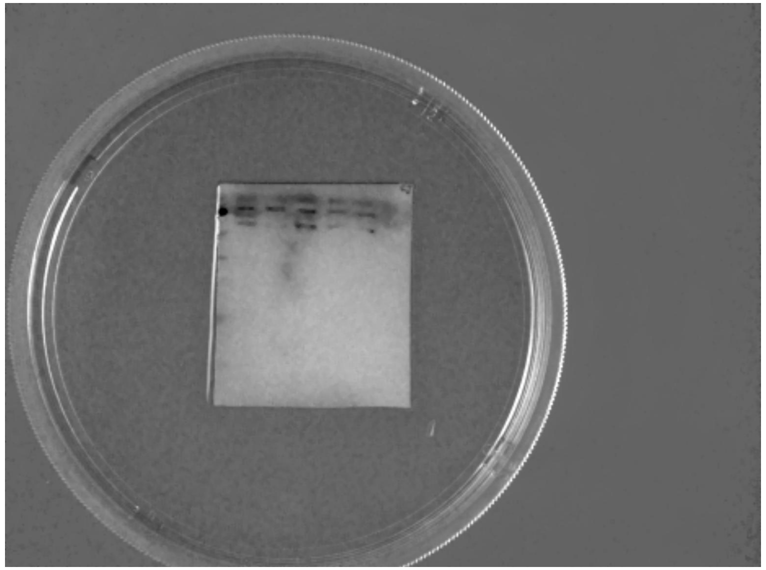

β-Actin

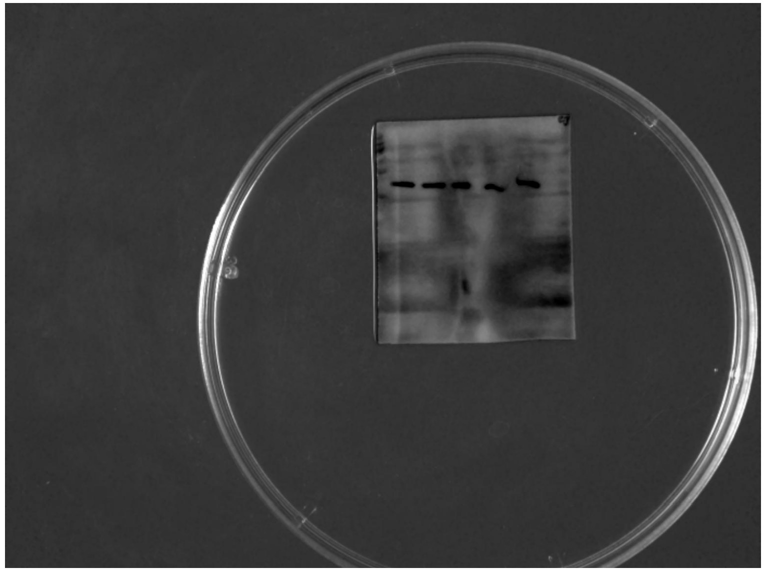

Figure S3J.The protein expression levels of NLRP3 in each group were detected using Western blotting.

CASPASE8

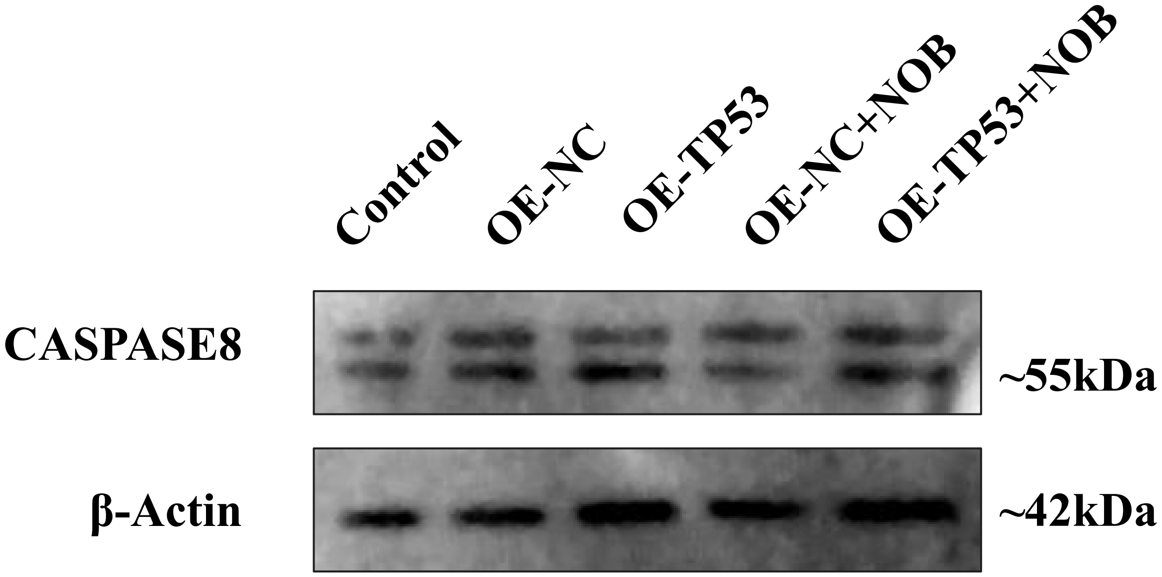

Figure S3K.The protein expression levels of CASPASE8 in each group were detected using Western blotting.

TP53 overexpression

CASPASE8

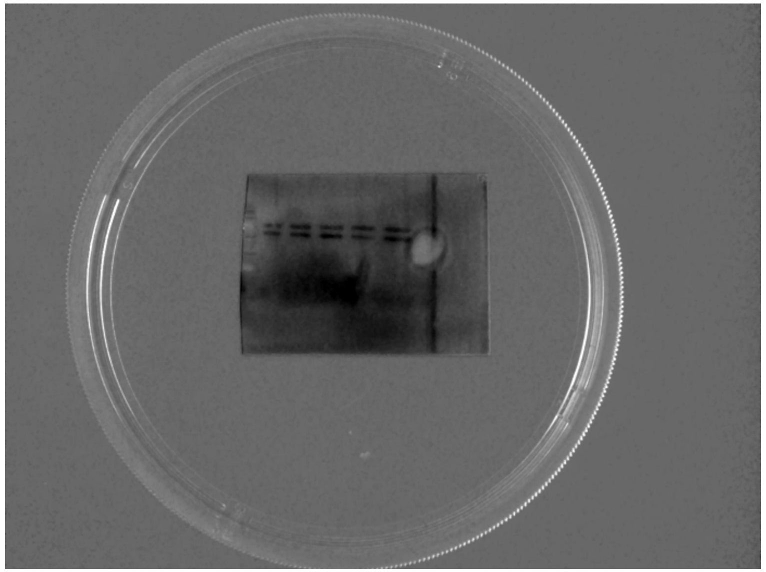

β-Actin

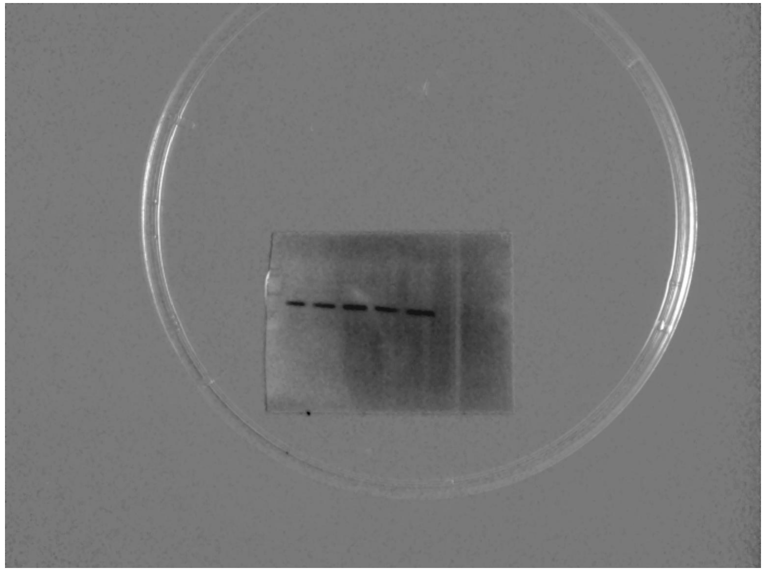

TFEB

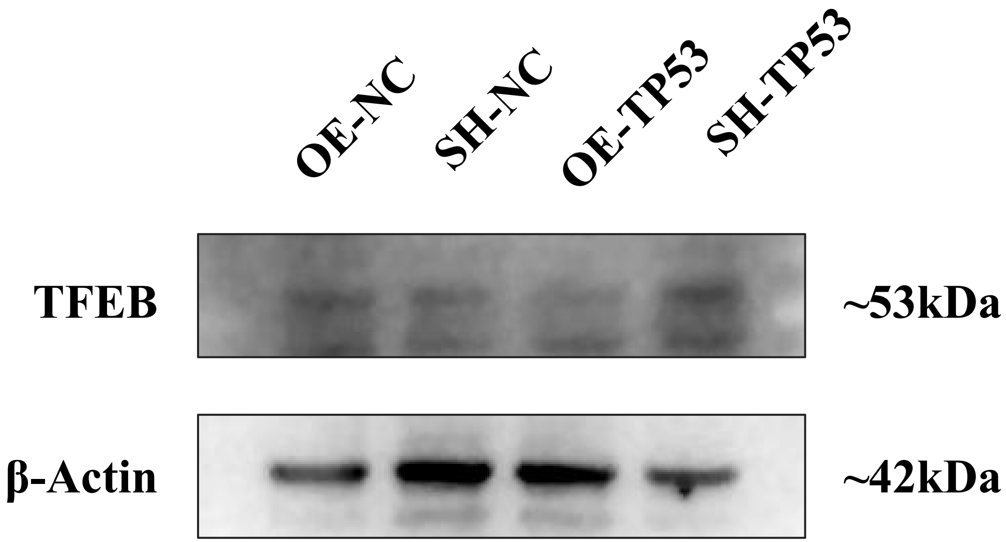

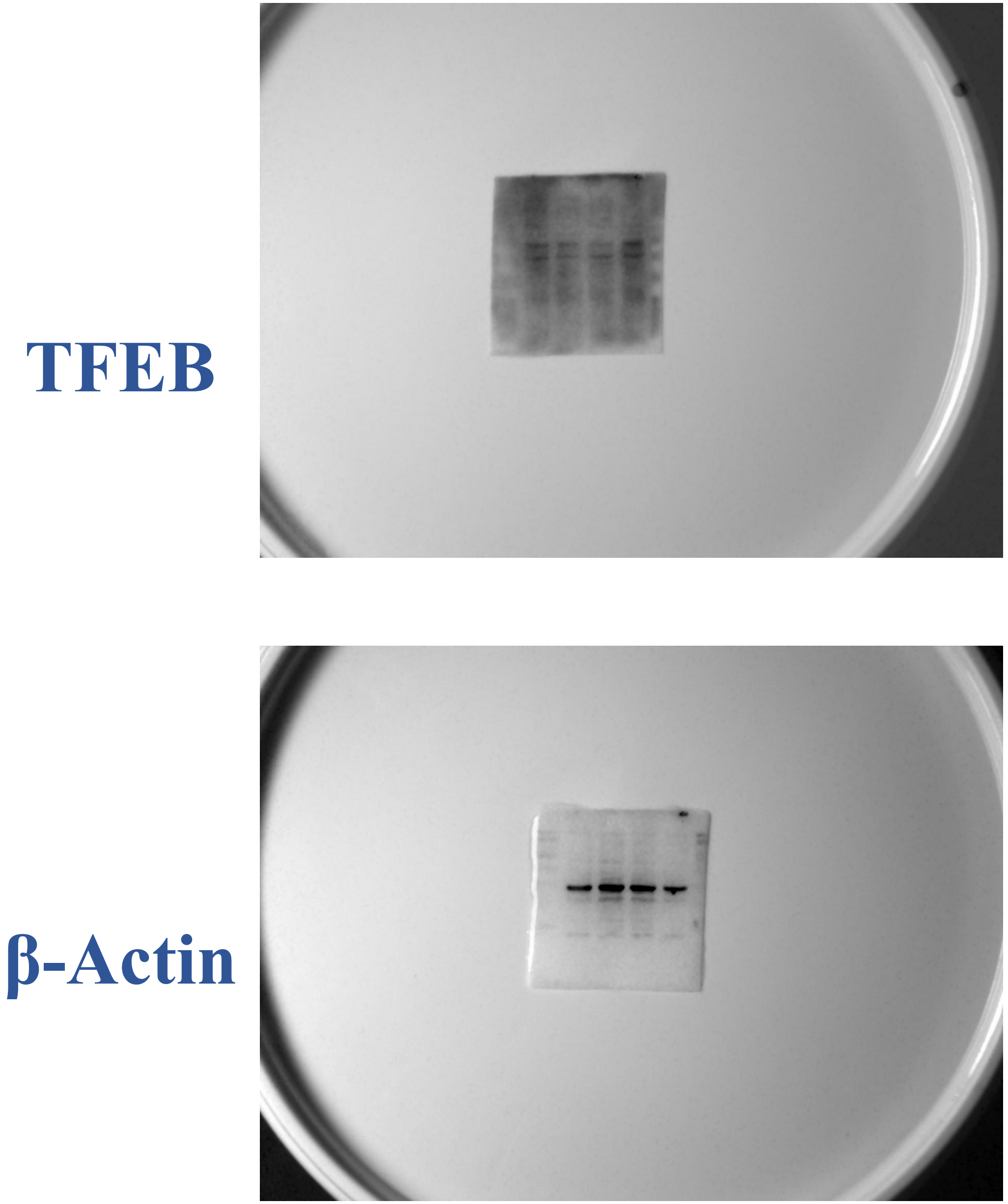

Figure S3L.The protein expression levels of TFEB in each group were detected using Western blotting.
