# Supplementary material for: Nobiletin ameliorates diabetic nephropathy by modulating TP53-associated PANoptosis: an experimental study
Source: Front Nutr. 2026 Sep 4;13:1816623. doi: 10.3389/fnut.2026.1816623 (PMC13585718; doi:10.3389/fnut.2026.1816623)
Supplement: Supplementary file 1 [file Data_Sheet_1.zip › Data Sheet 1/Supplementary Figure S4.PDF]

Figure9A Pyroptosis

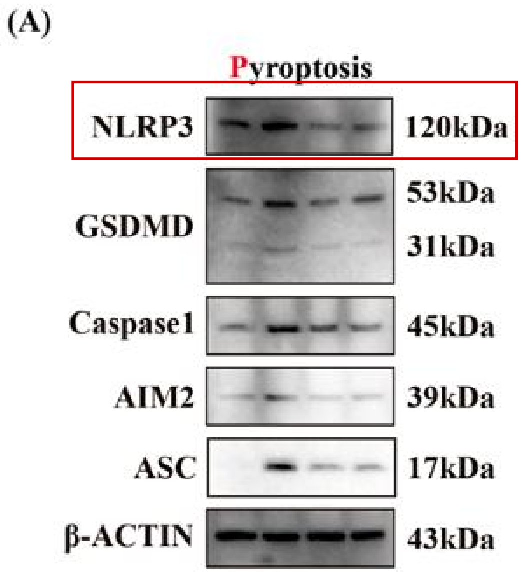

NLRP3

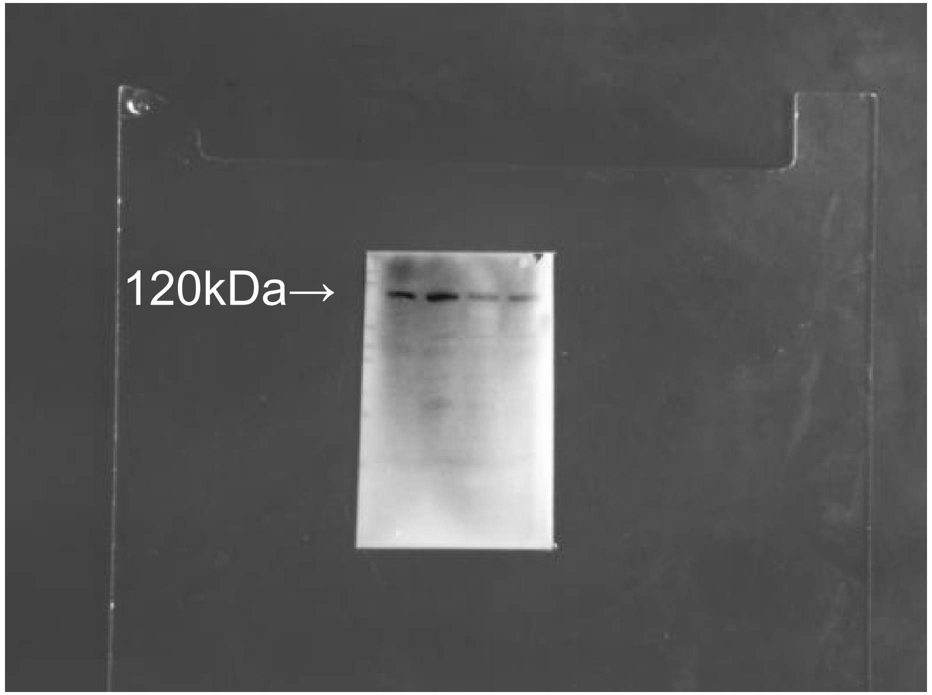

β-Actin

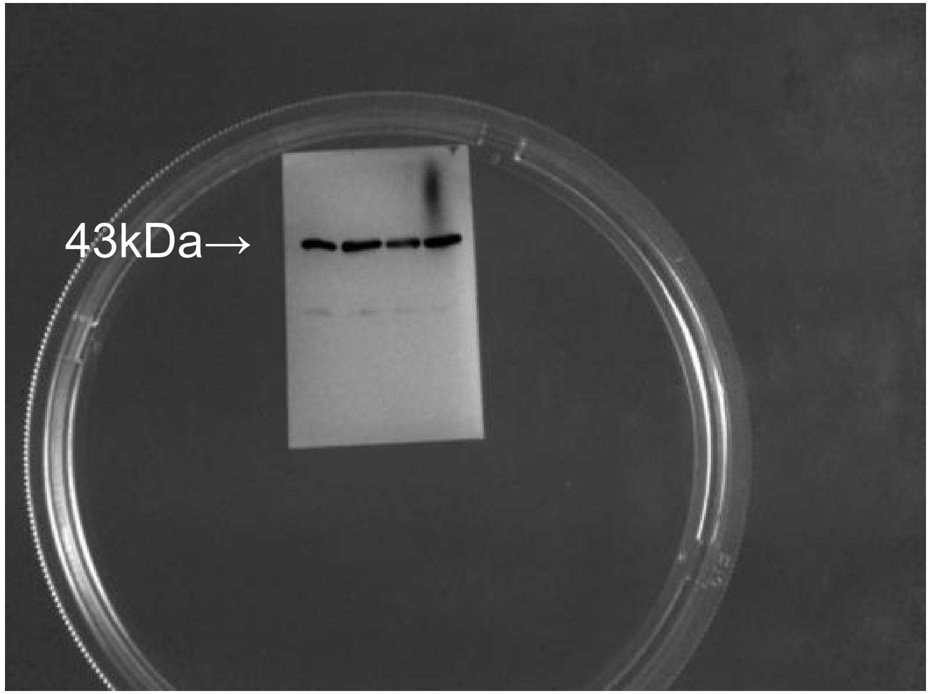

nlrp3→（stripping buffer）→actin

Figure9A Pyroptosis

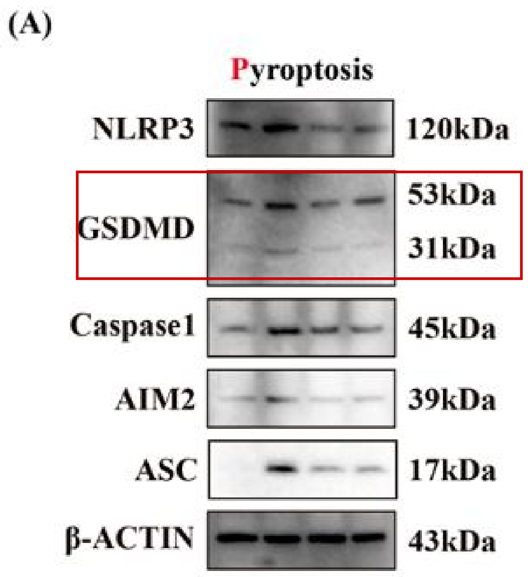

GSDMD

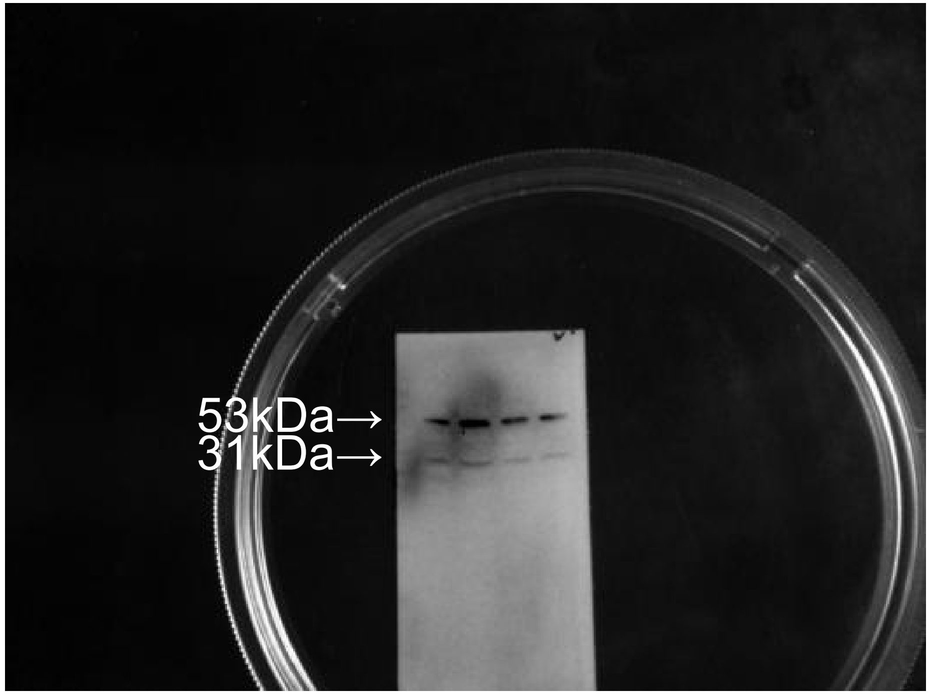

β-Actin

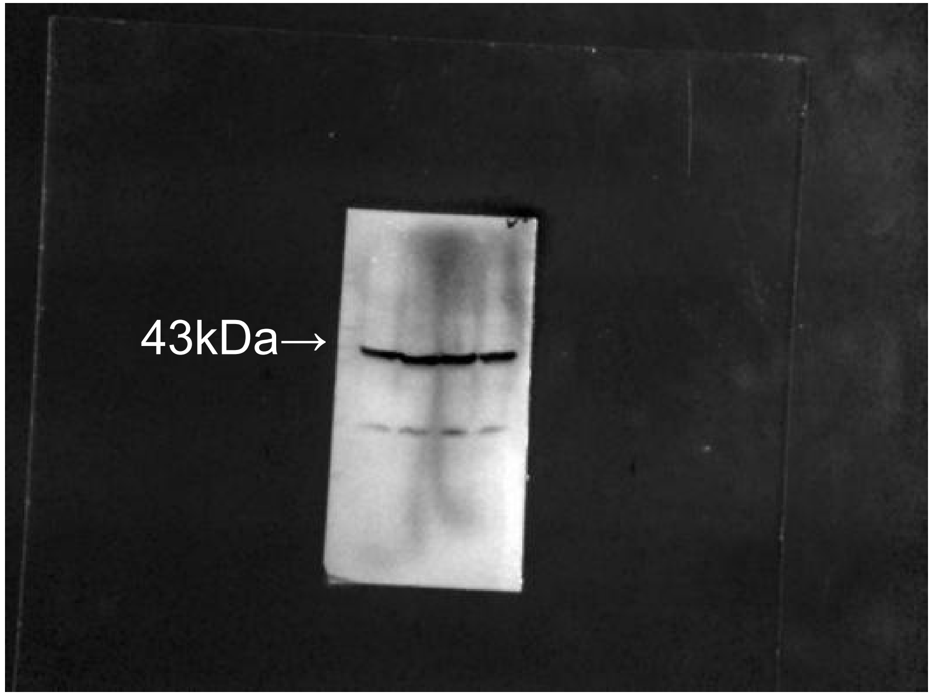

gsdmd→（stripping buffer）→actin

Figure9A Pyroptosis

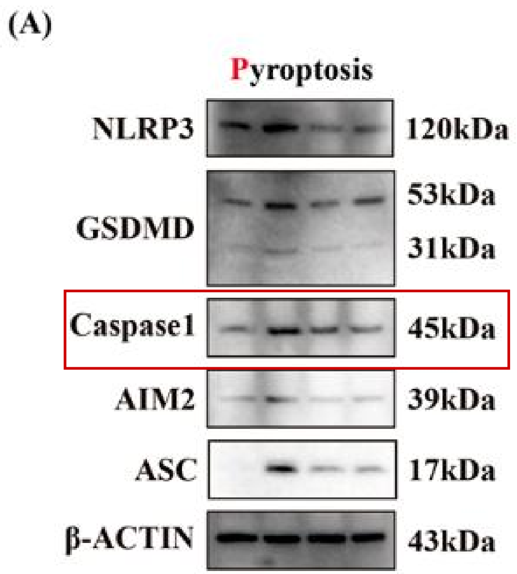

Caspase1

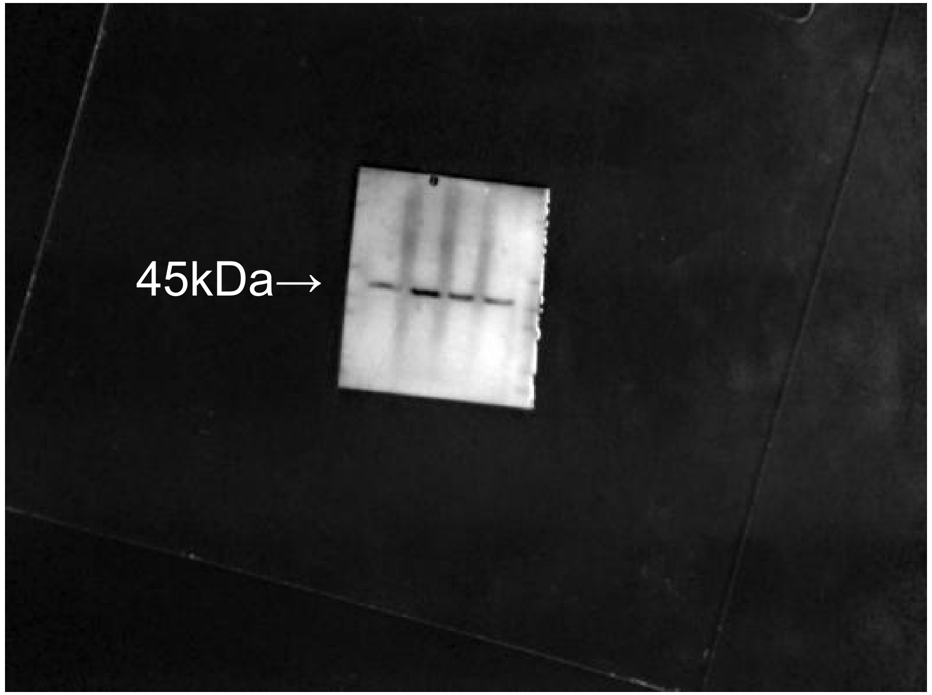

β-Actin

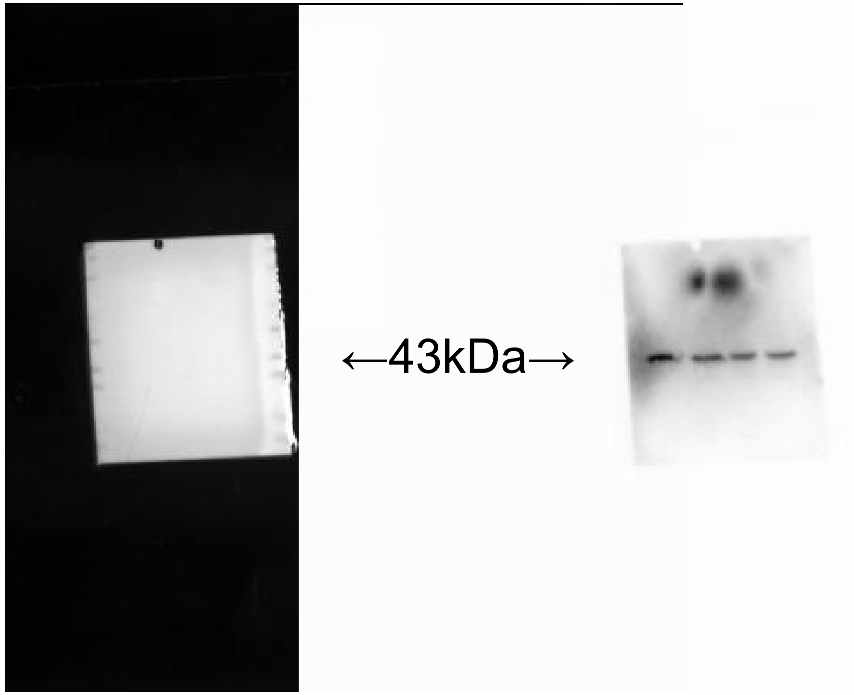

caspase1→（stripping buffer）→actin

Figure9A Pyroptosis

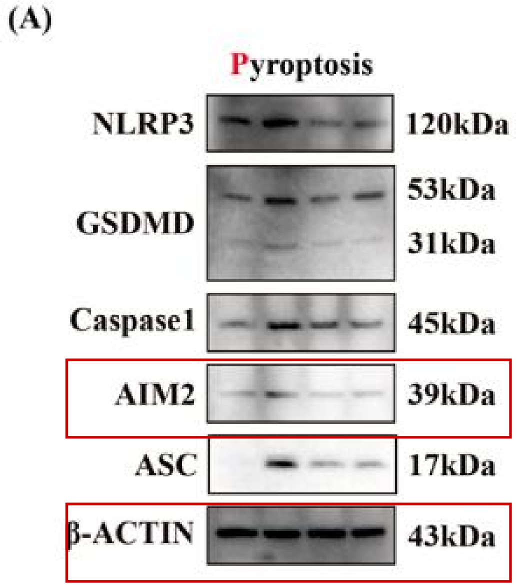

AIM2

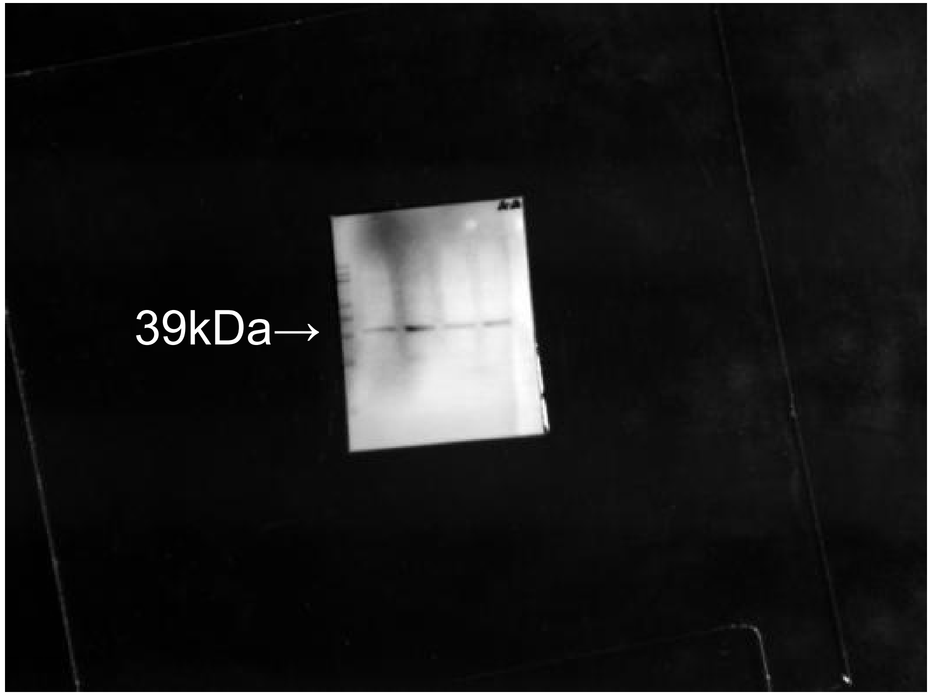

β-Actin

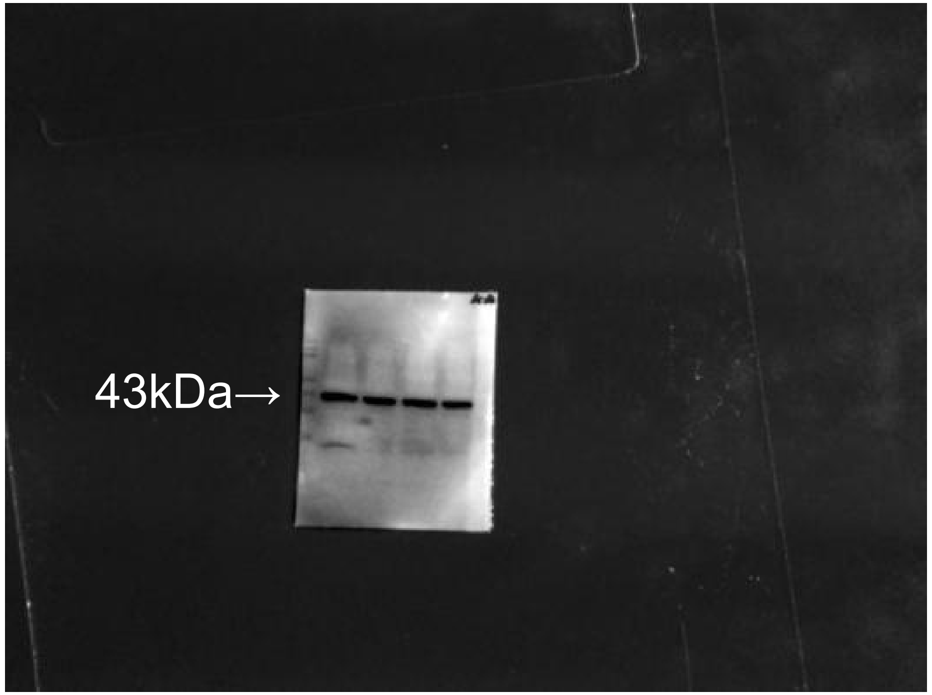

aim2→（stripping buffer）→actin

Figure9A Pyroptosis

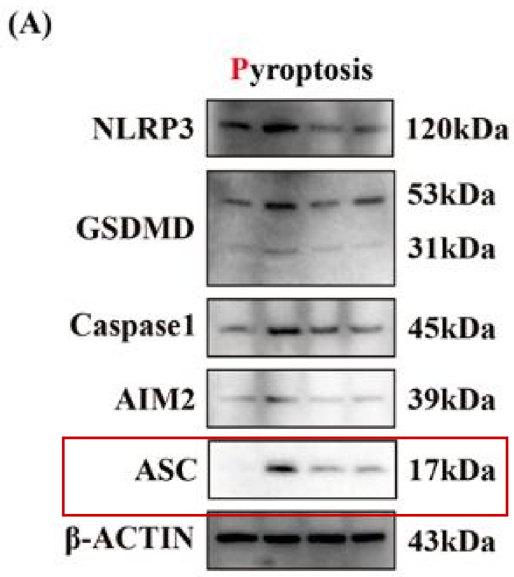

ASC

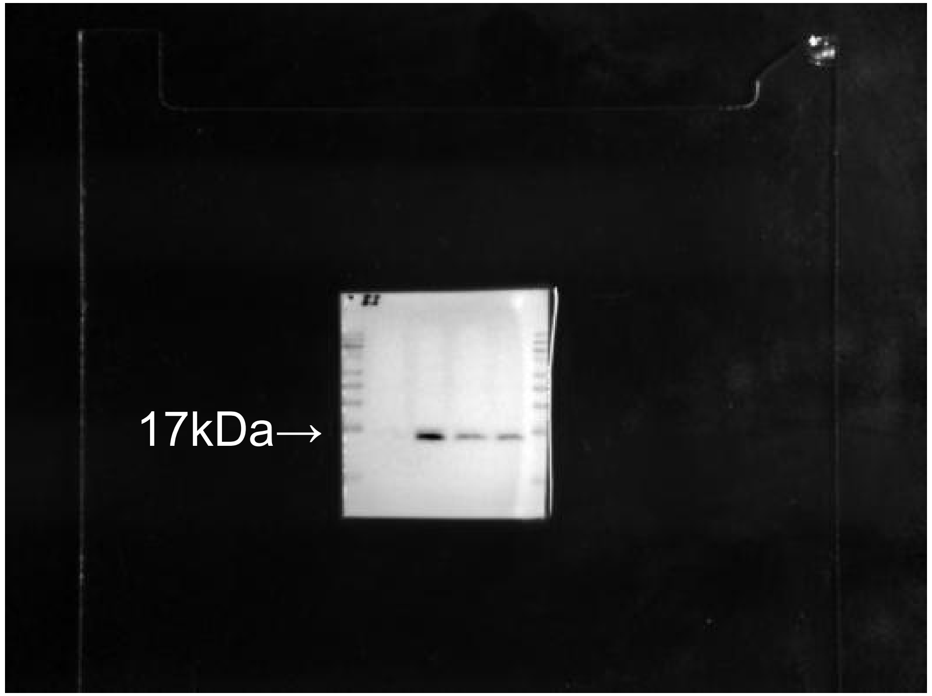

β-Actin

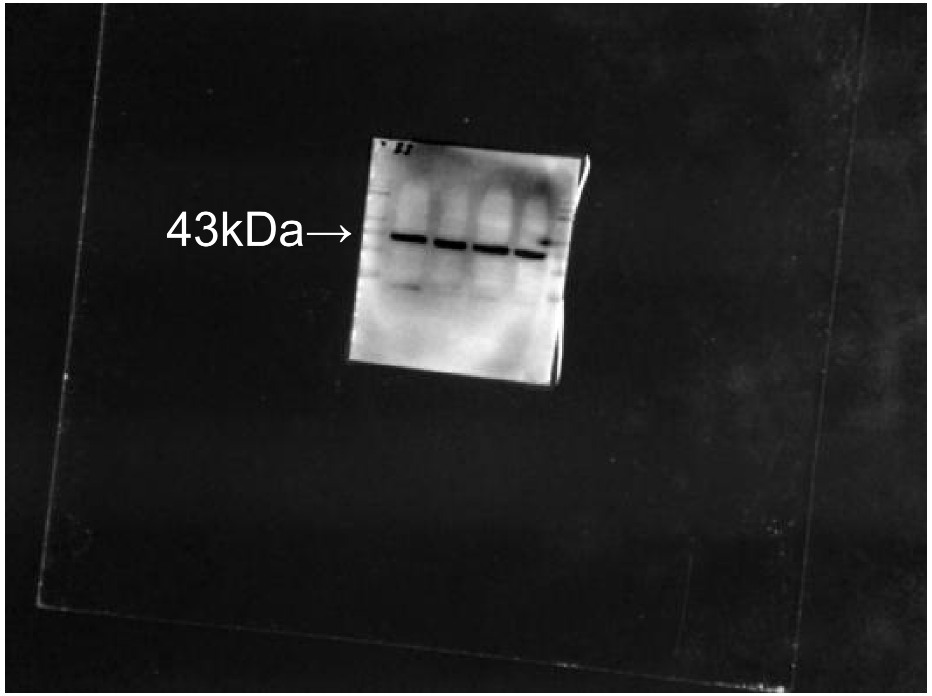

asc→（stripping buffer）→actin

Figure9C Apoptosis

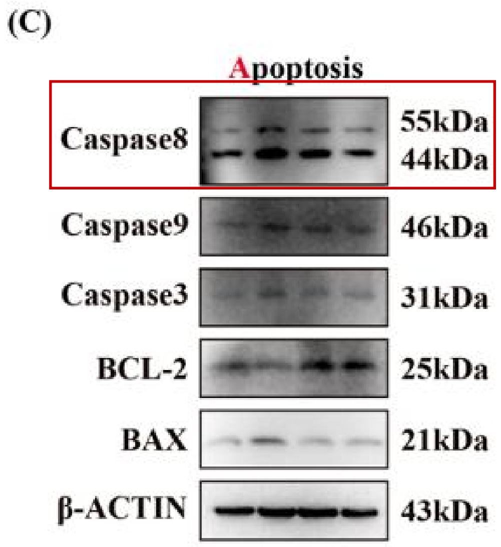

Caspase8

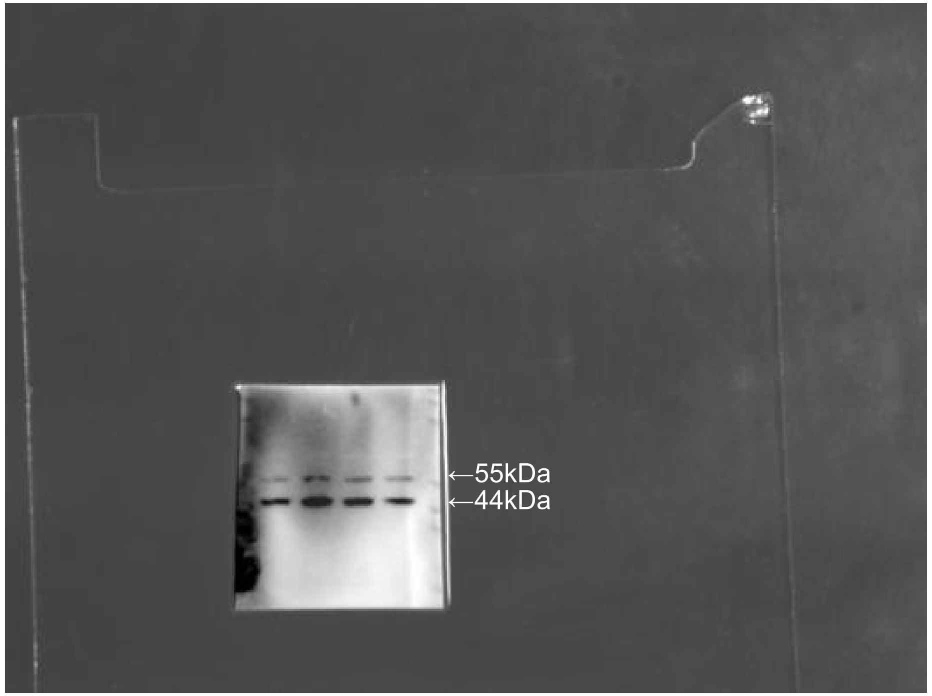

β-Actin

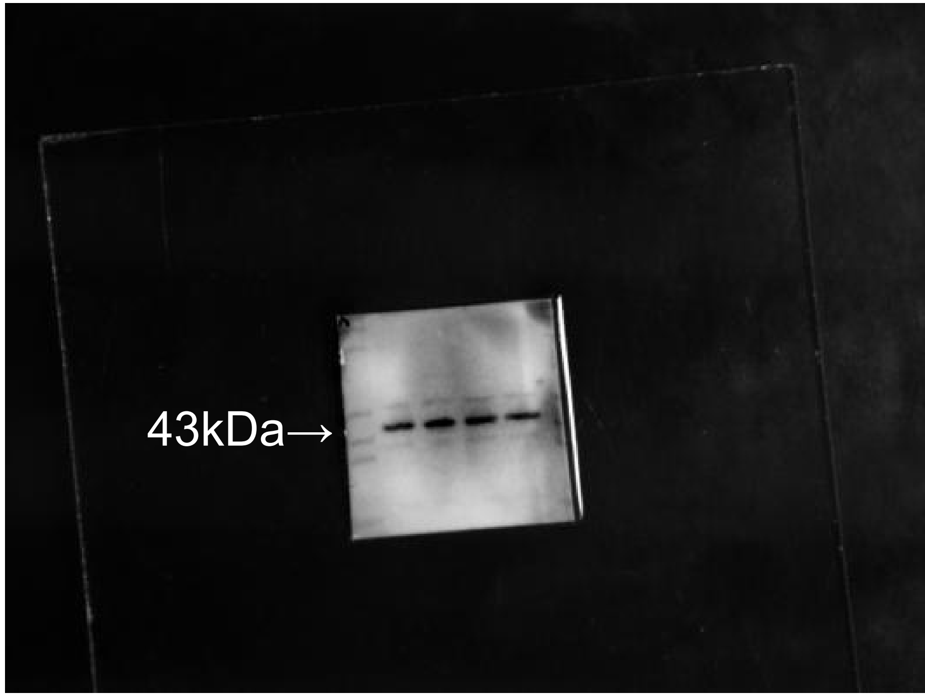

Caspase8→（stripping buffer）→actin

Figure9C Apoptosis

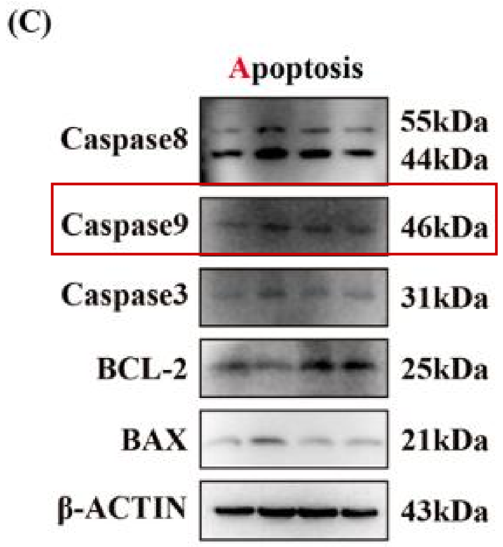

caspase9

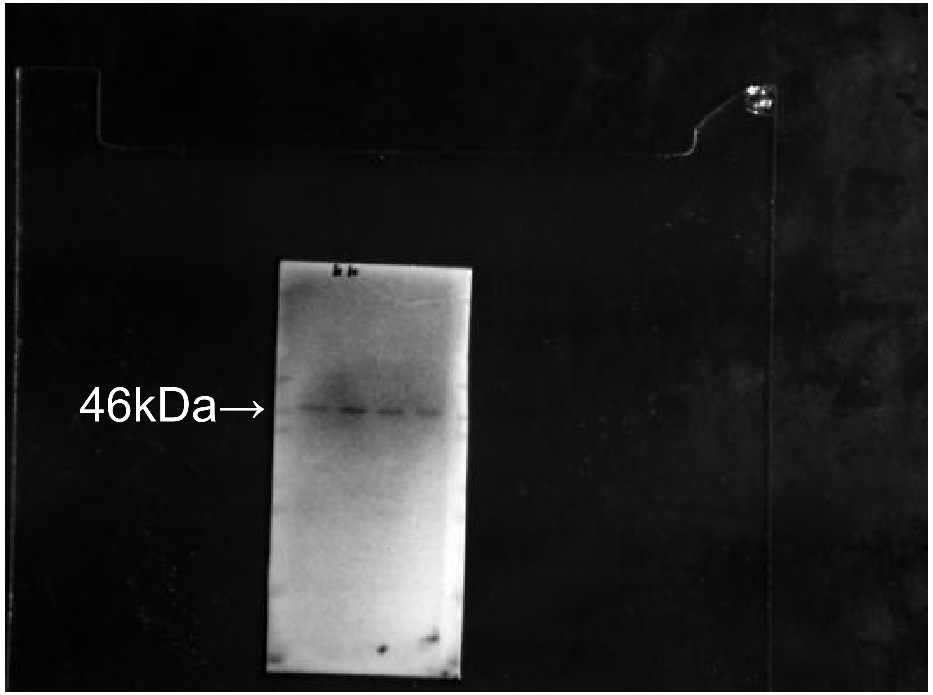

β-Actin

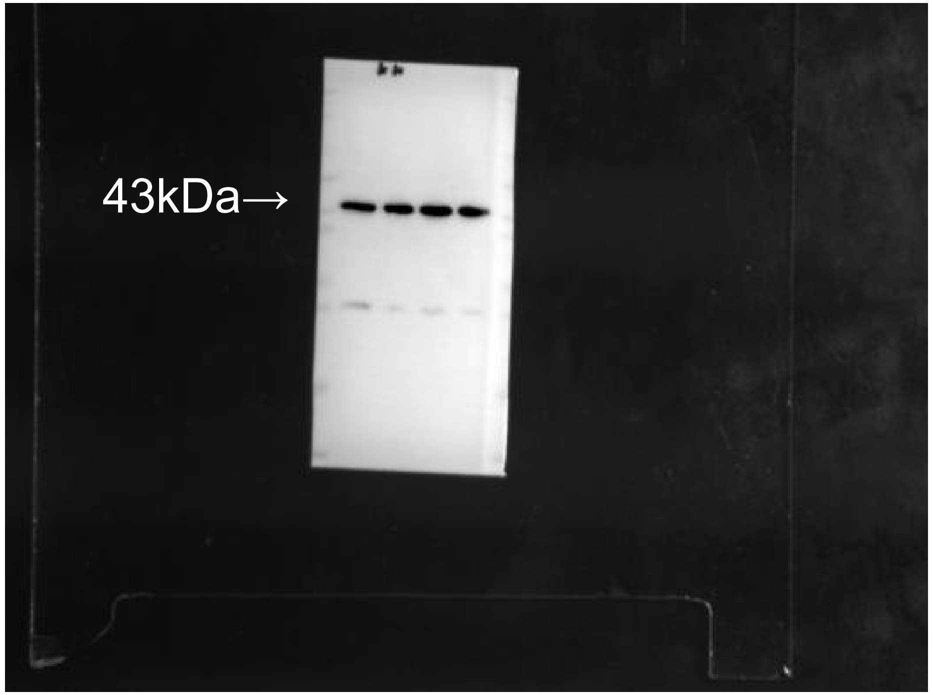

caspase9→（stripping buffer）→actin

Figure9C Apoptosis

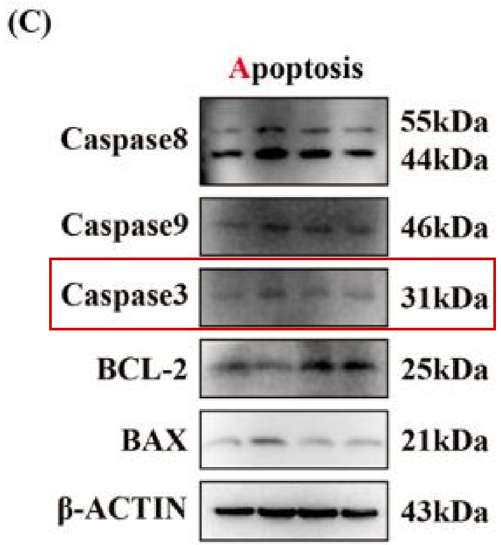

caspase3

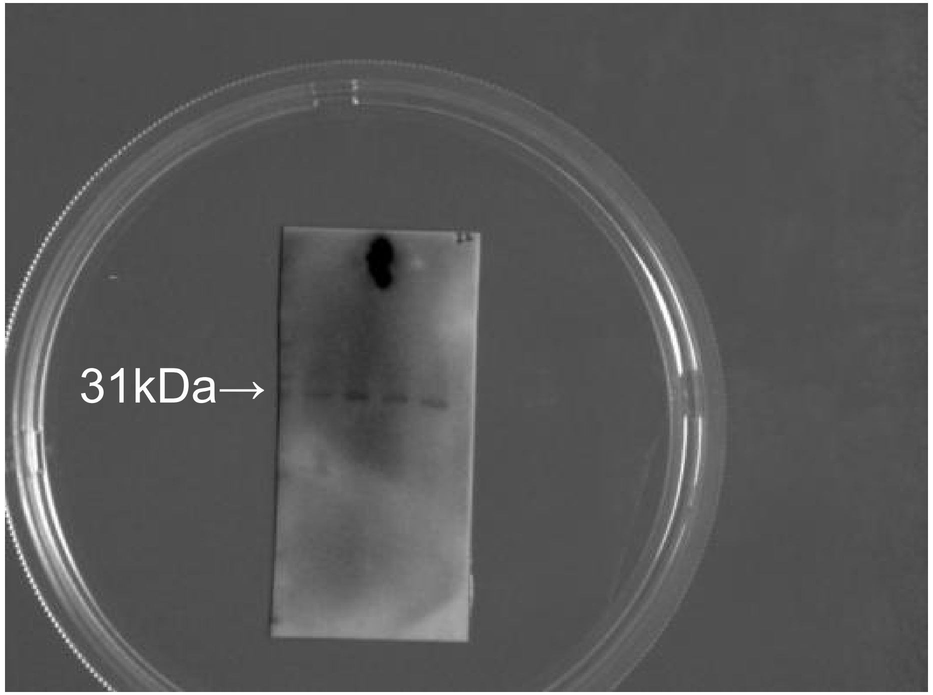

β-Actin

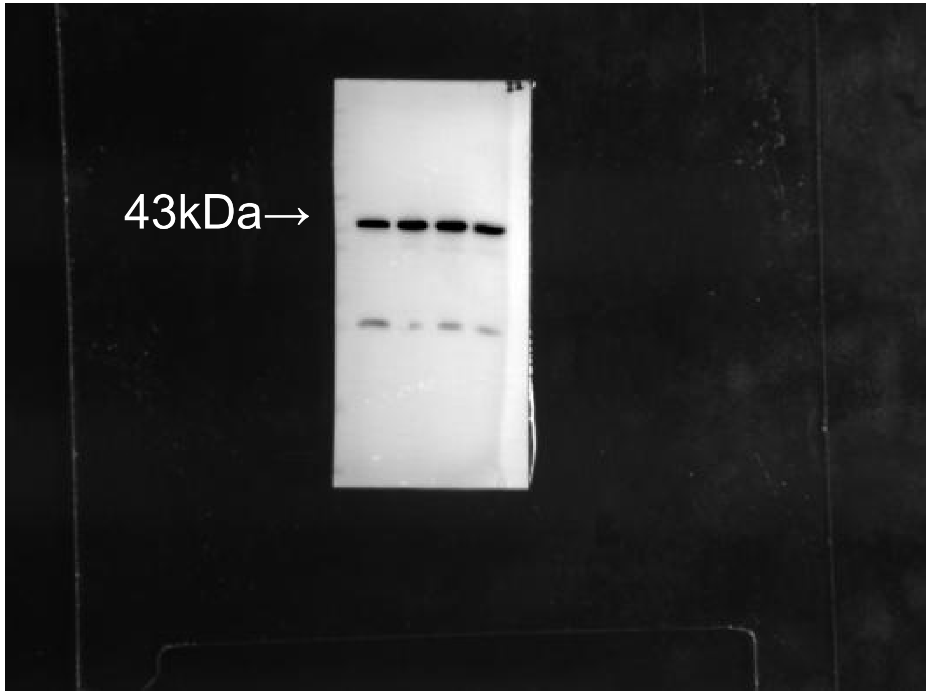

caspase3→（stripping buffer）→actin

Figure9C Apoptosis

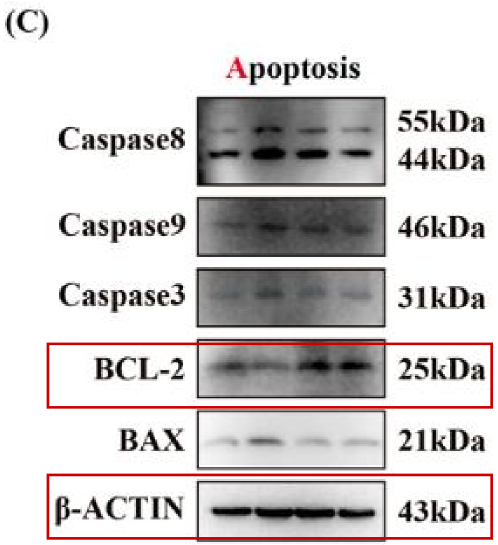

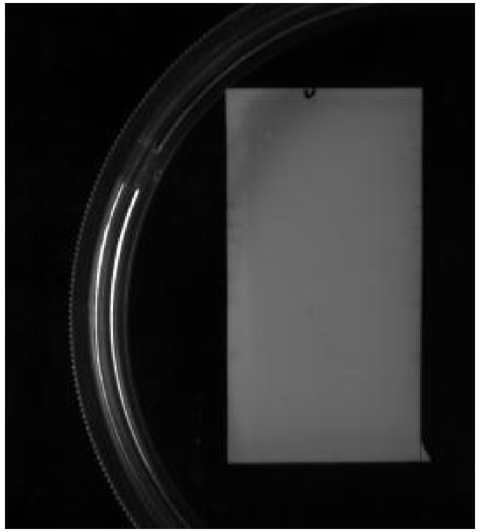

BCL-2

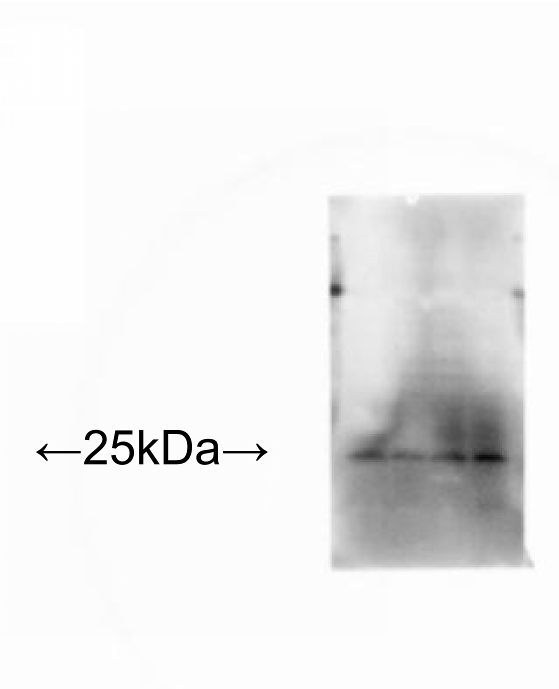

β-Actin

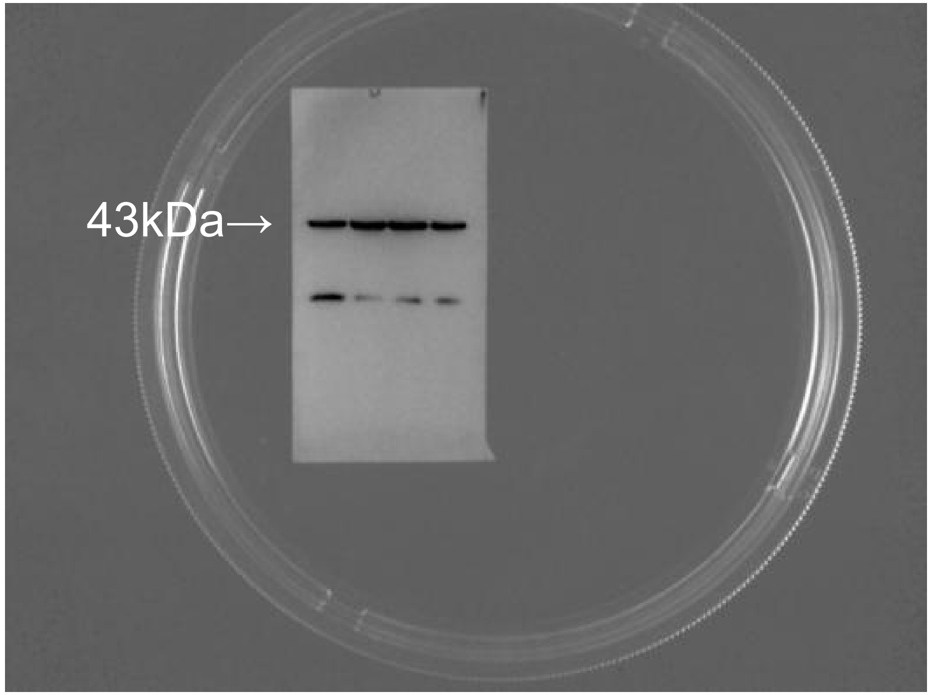

bcl-2→（stripping buffer）→actin

Figure9C Apoptosis

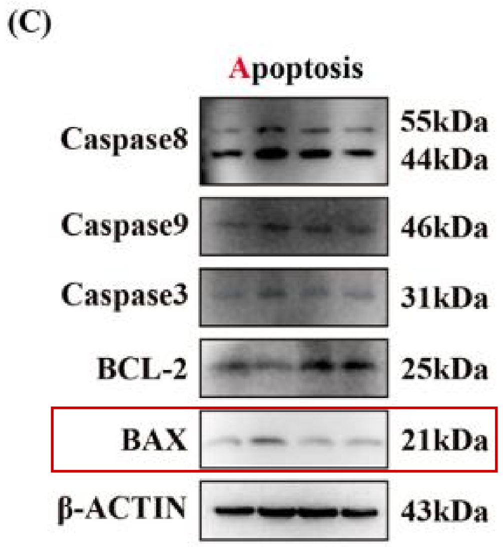

BAX

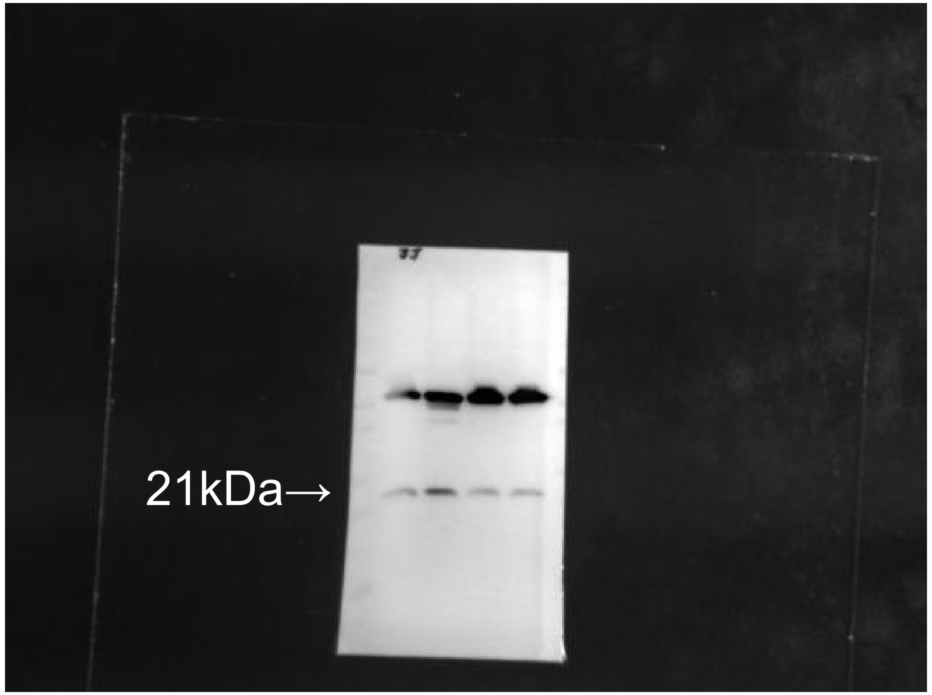

β-Actin

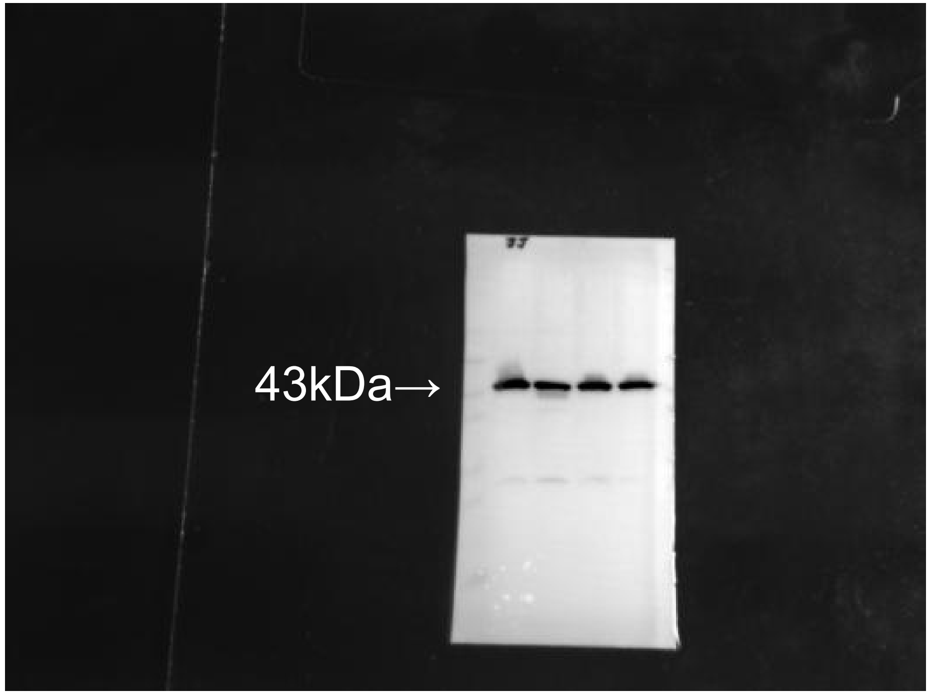

actin→（stripping buffer）→bax

Figure9E Necroptosis

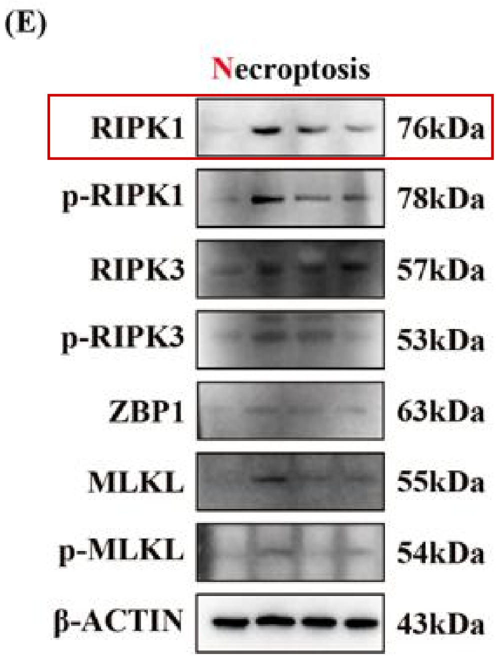

RIPK1

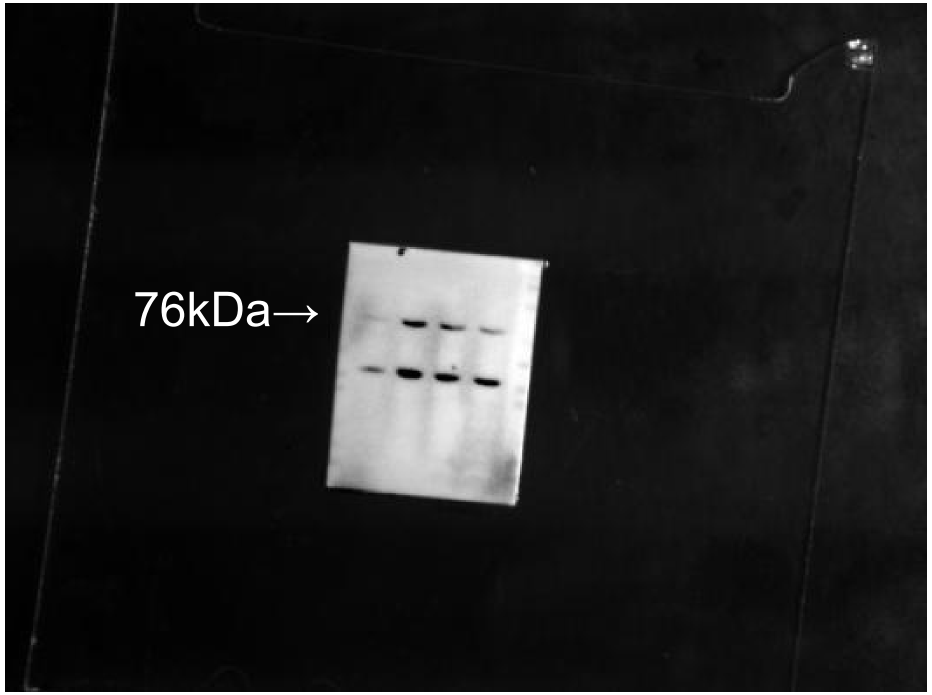

β-Actin

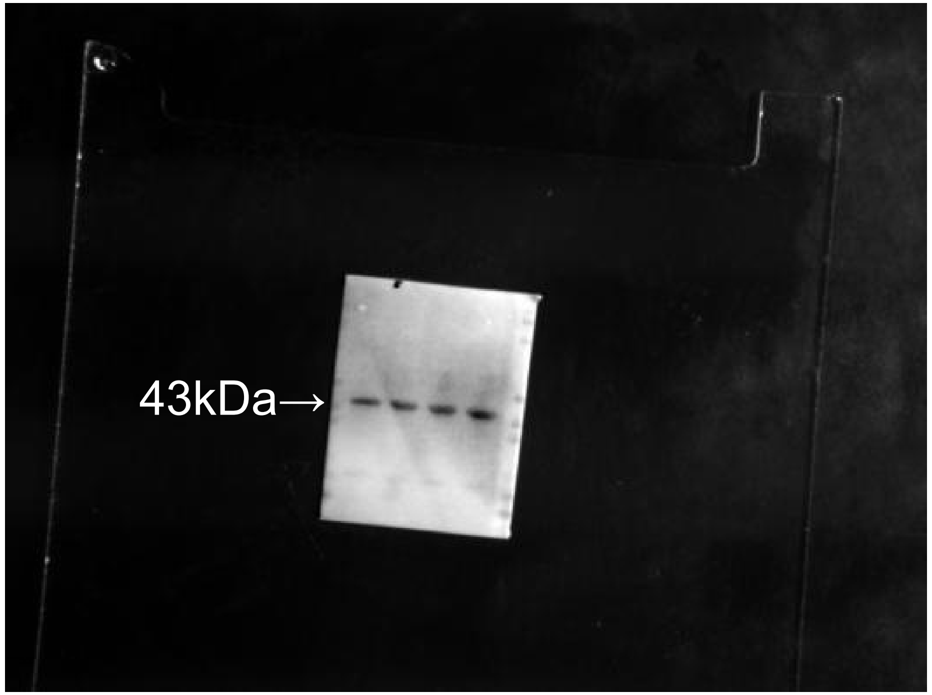

actin→（stripping buffer）→ripk1

Figure9E
Necroptosis

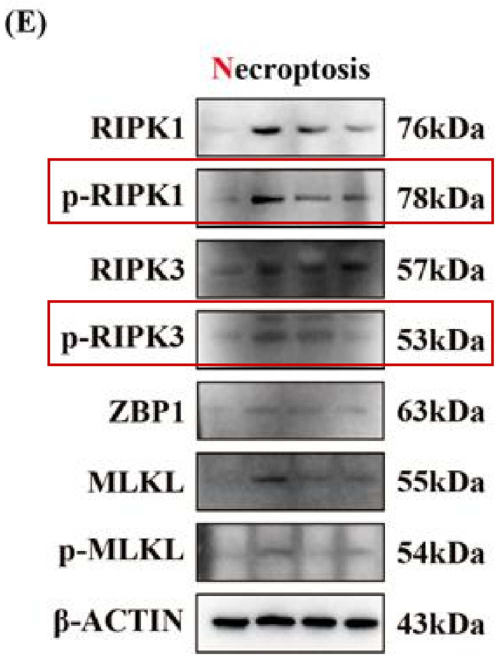

P-RIPK1

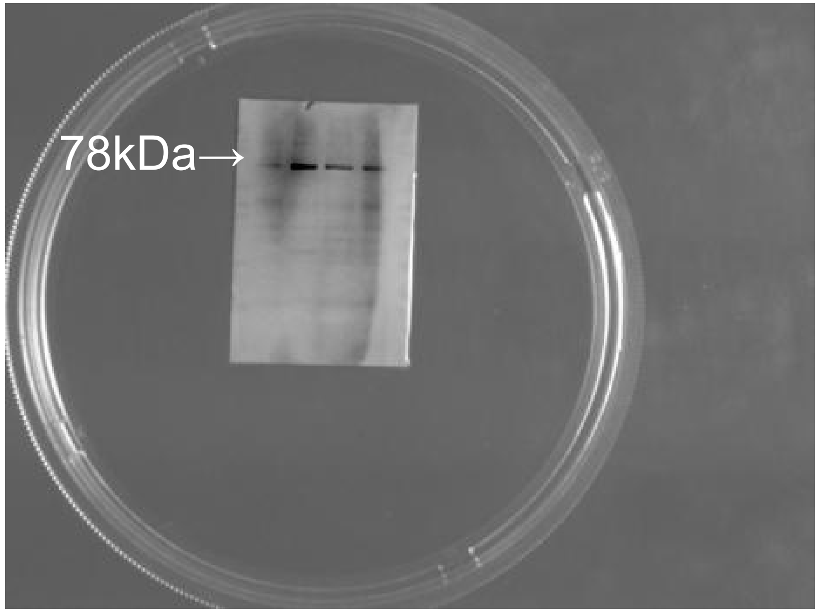

P-RIPK3

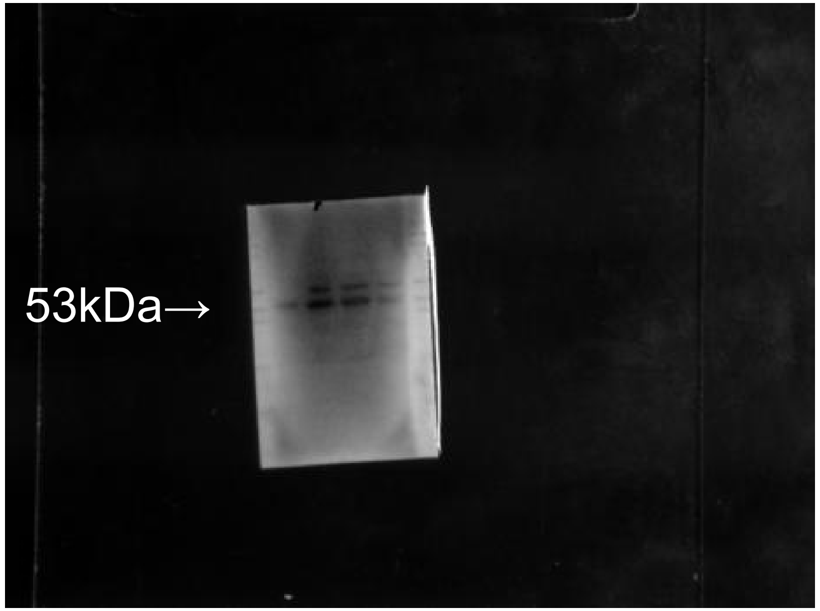

β-Actin

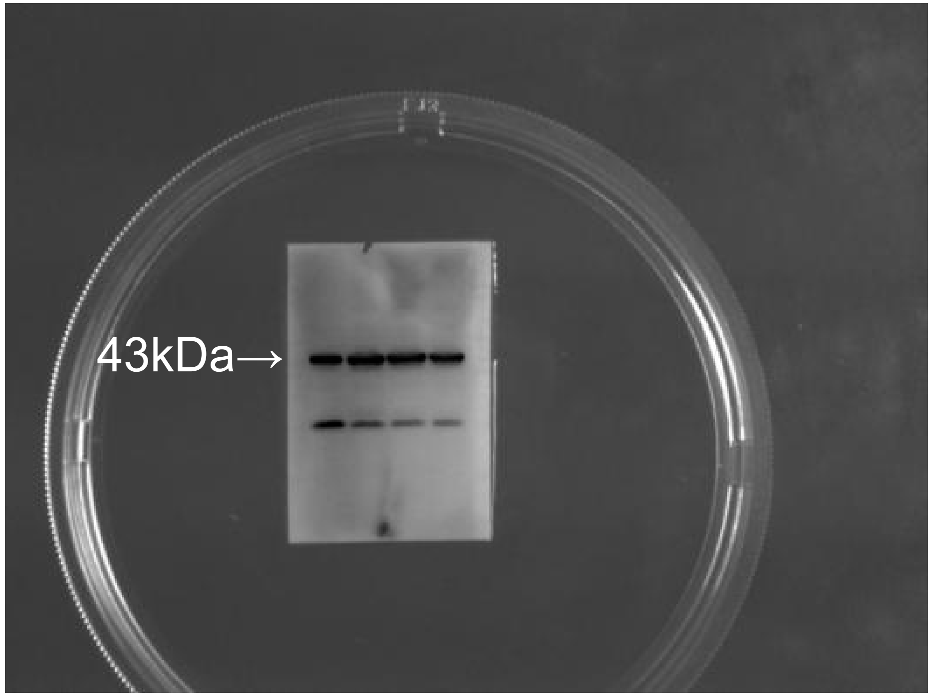

p-ripk1→（stripping buffer）→p-ripk3→（stripping buffer）→actin

Figure9E Necroptosis

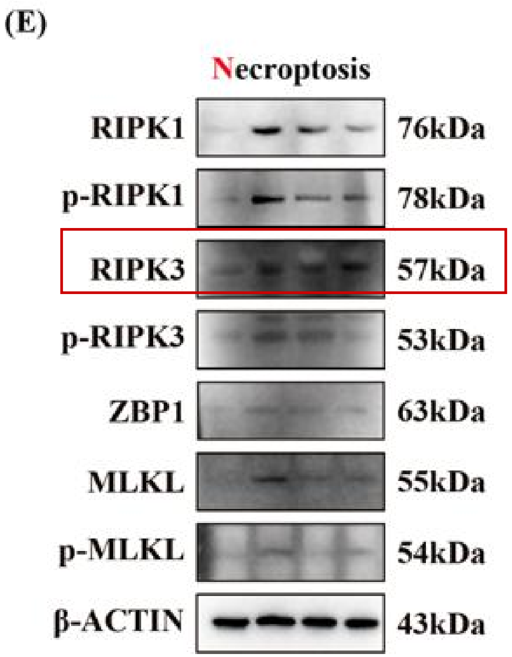

RIPK3

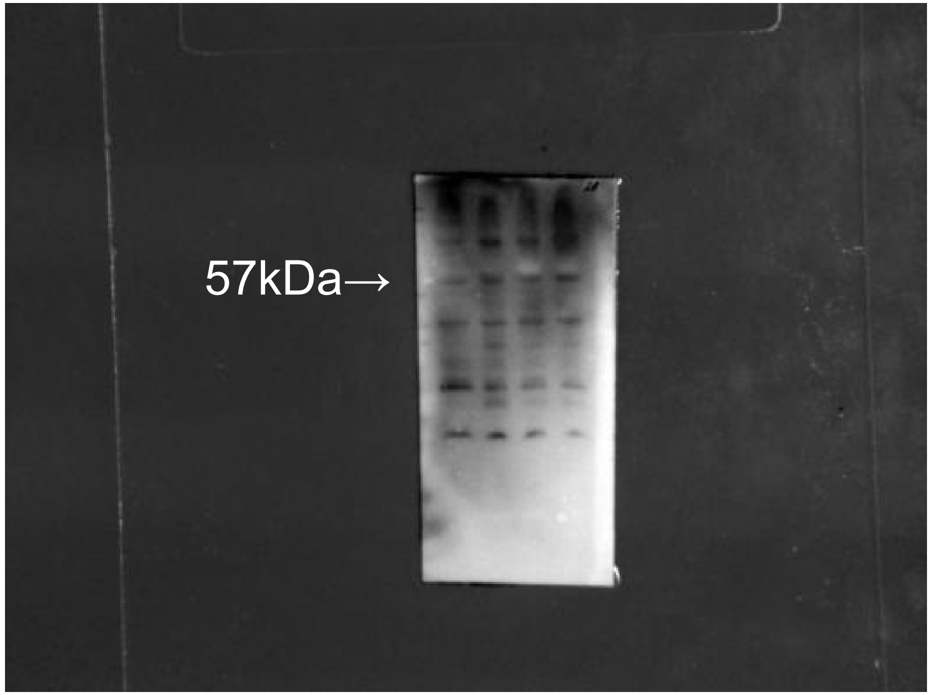

β-Actin

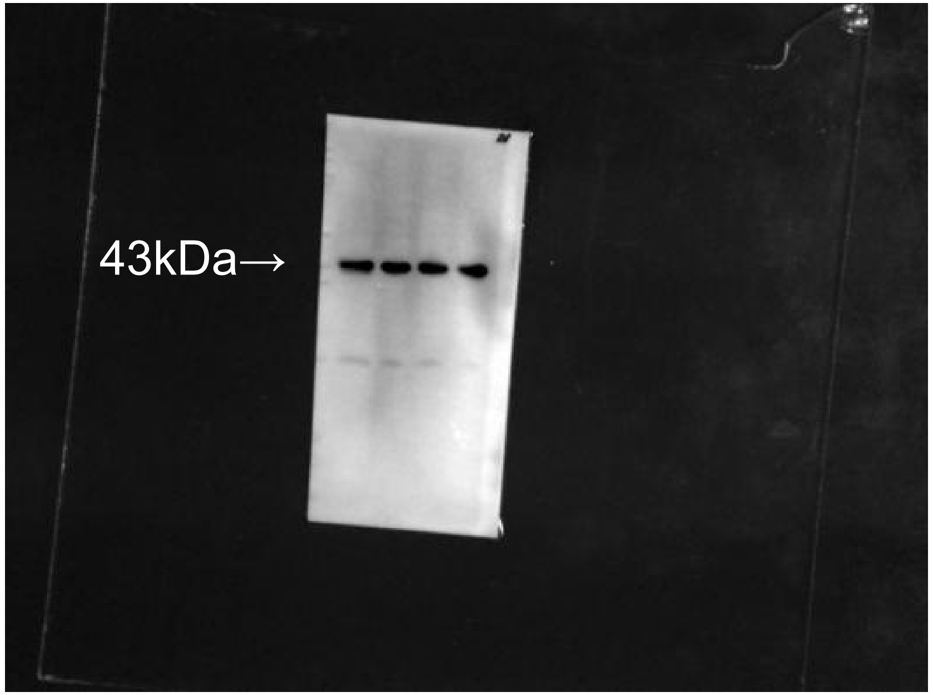

RIPK3→（stripping buffer）→actin

Figure9E Necroptosis

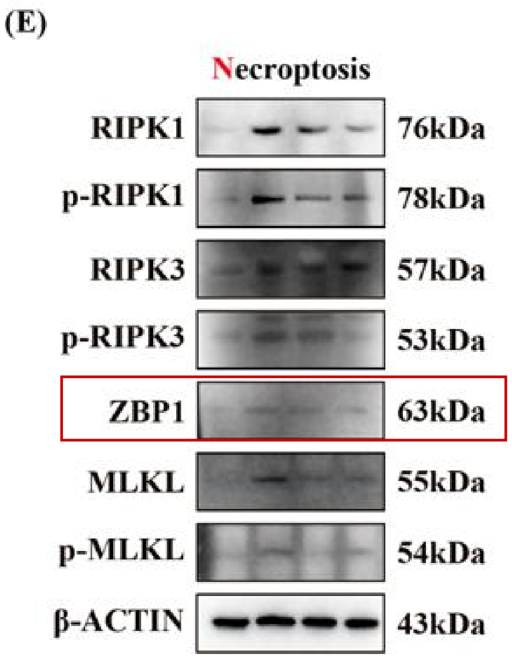

ZBP1

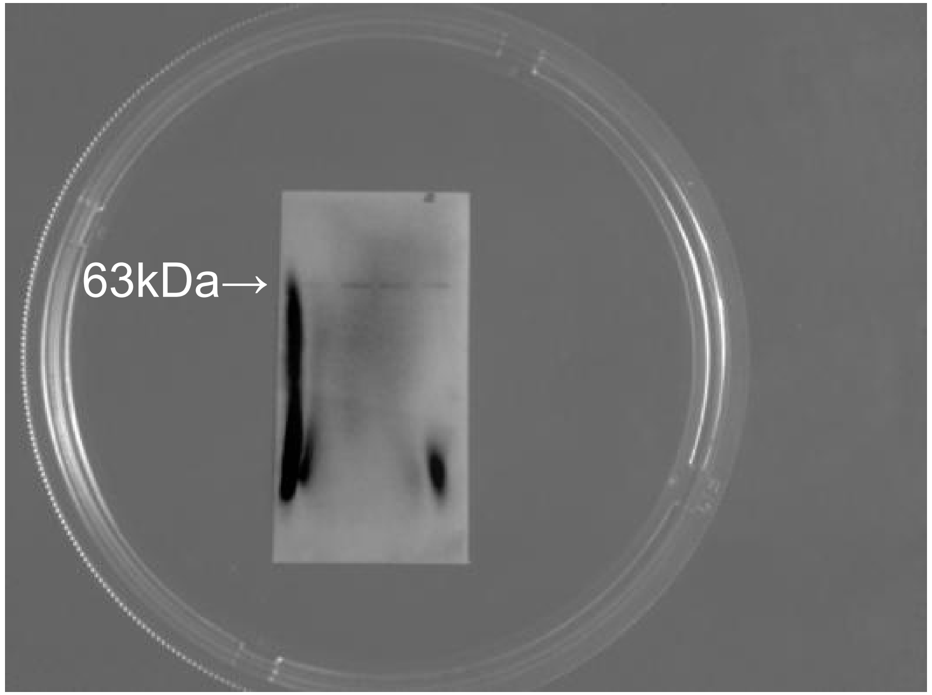

β-Actin

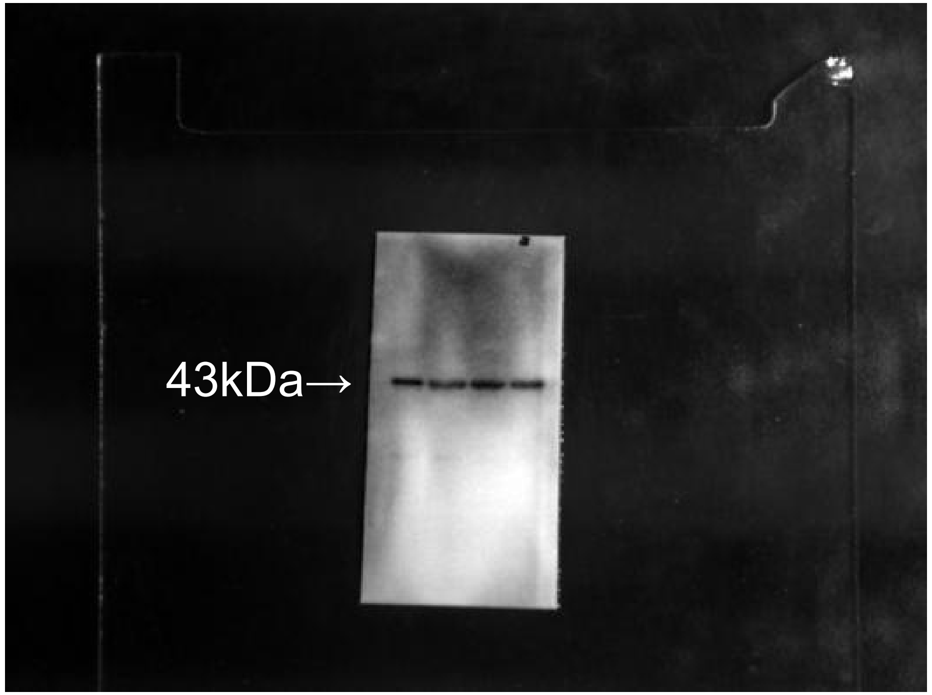

zbp1→（stripping buffer）→actin

Figure9E Necroptosis

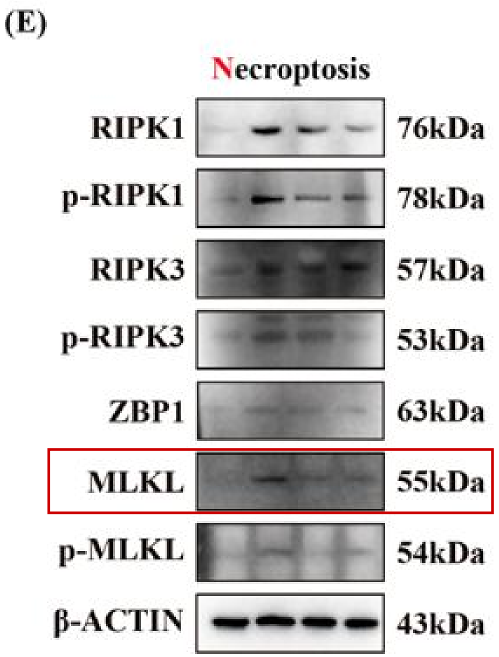

MLKL

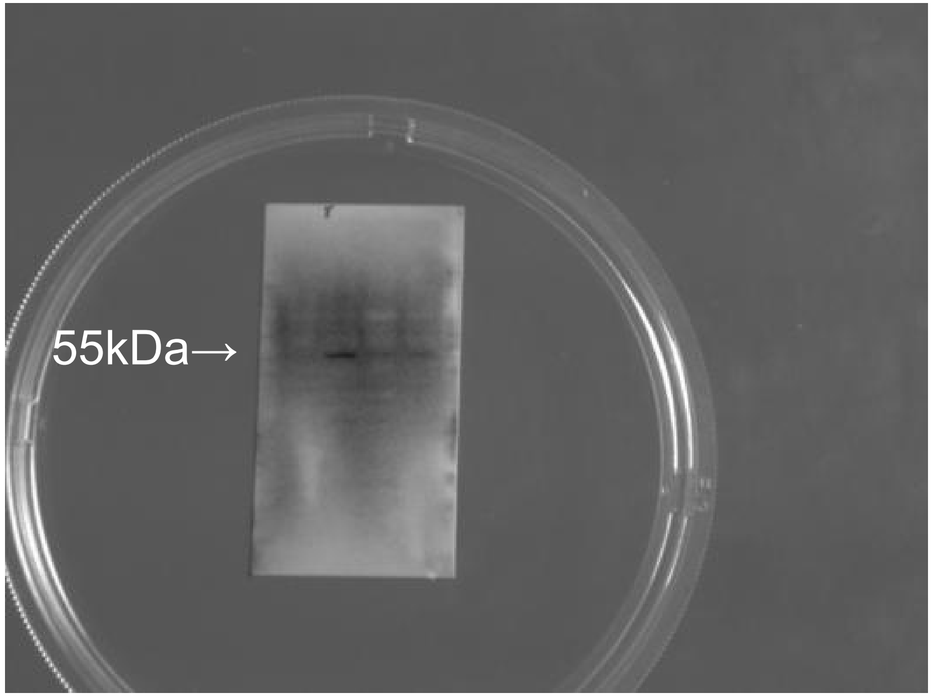

β-Actin

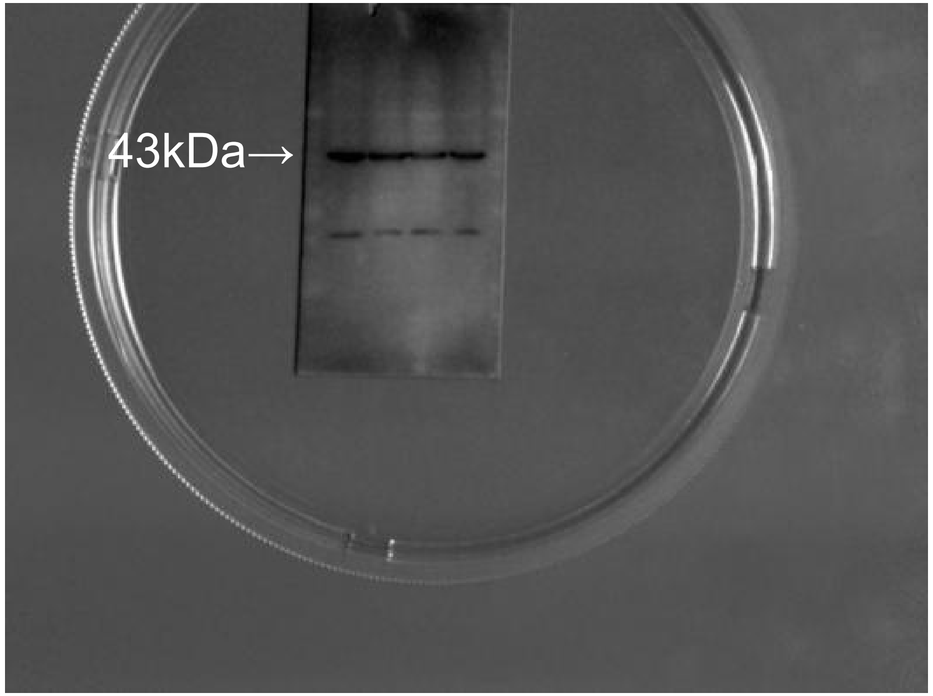

mlkl→（stripping buffer）→actin

Figure9E Necroptosis

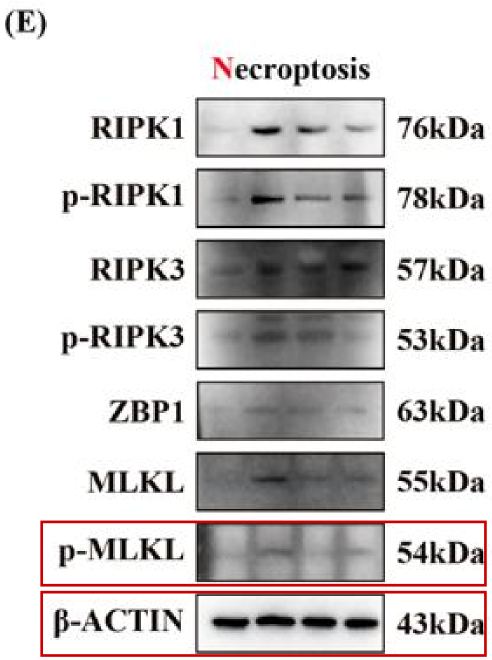

p-MLKL

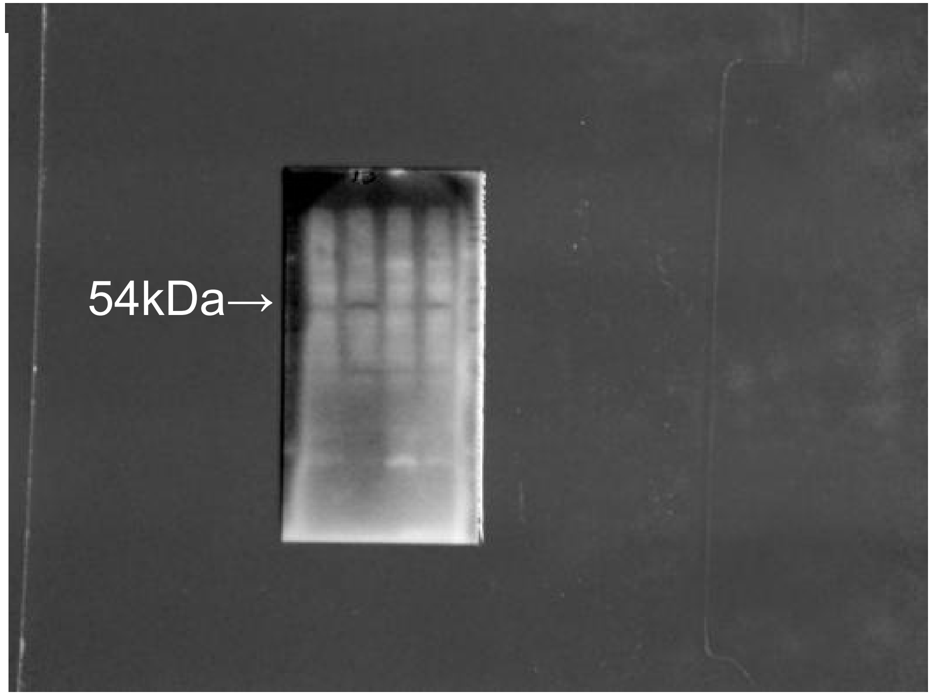

β-Actin

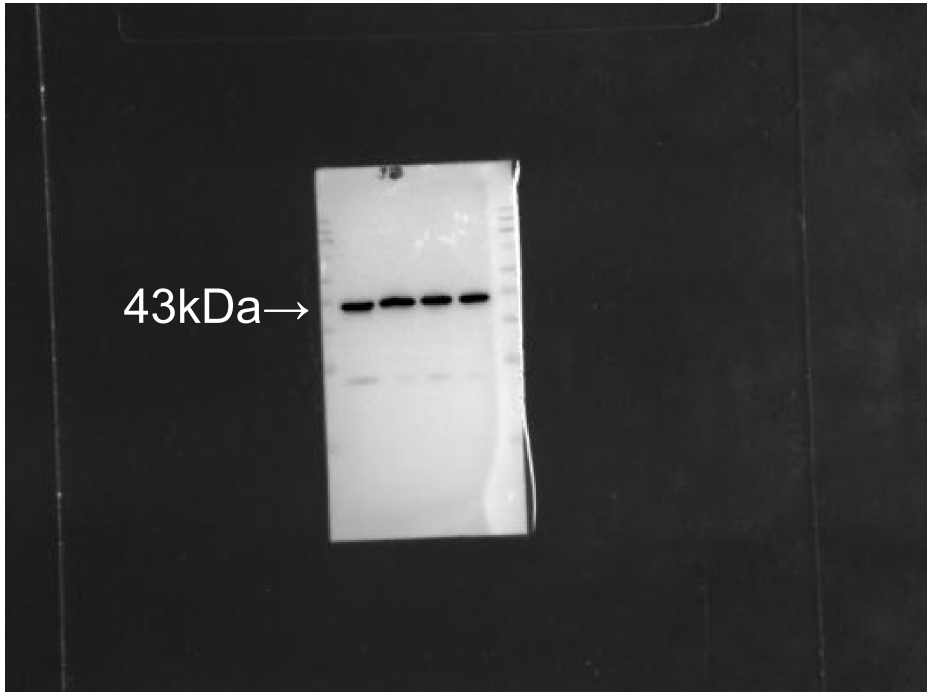

p-mlkl→（stripping buffer）→actin
