# Supplementary material for: Nobiletin ameliorates diabetic nephropathy by modulating TP53-associated PANoptosis: an experimental study
Source: Front Nutr. 2026 Sep 4;13:1816623. doi: 10.3389/fnut.2026.1816623 (PMC13585718; doi:10.3389/fnut.2026.1816623)
Supplement: Supplementary file 1 [file Data_Sheet_1.zip › Data Sheet 1/Supplementary File S1.PDF]

华北理工大学 NORTH CHINA UNIVERSITY OF SCIENCE AND TECHNOLOGY

Laboratory Animal Ethical and Welfare Committee
North China University of Science and Technology

实验动物福利伦理审查同意书

Approval of Laboratory Animal Ethical and Welfare

编号 Approval No.	2024SY3097

本《动物实验方案》经过华北理工大学实验动物福利伦理委员会审核，符合动物保护、动物福利和伦理原则，符合国家实验动物福利伦理的相关规定。

The protocol of animal experiment listed below was reviewed and approved by the Laboratory Animal Ethical and Welfare Committee of North China University of Science and Technology.

课题名称 Protocol Title	川陈皮素通过调节代谢改善糖尿病肾病的机制研究<br>The mechanism of nobiletin improveing diabetic nephropathy by regulating metabolism				
课题负责人 Principle Investigator (PI)	李继安<br>Li Ji'an	职称/学位 Title/Degree	教授/博士<br>Professor/PhD	邮箱 Email	lnyy@vip.sina.com
动物实验负责人 Contact Person	刘一<br>Liu Yi	职称/学位 Title/Degree	博士研究生<br>Doctoral student	邮箱 Email	yiyiliu2021@163.com
院系(部门) Department	中医学院<br>College of Traditional Chinese Medicine		申请日期 Application date	2024.07.01	
动物种系 Species or Strains	db/db 小鼠、db/m 小鼠		动物数量 Animal Quantity	200、40	
计划执行时间 Period of Protocol	4个月	实验动物使用许可证号 Number of Animal use permit		SCXK(苏)2021-0013<br>SYXK(冀)2023-018	
审查意见 Results of inspection	□ 符合动物福利伦理要求，可以进行实验 Agree<br>□ 调整方案后，可以进行实验 Agree after modify				
兽医师 Chief Veterinary Officer	[illegible]		日期 Date	2024.7.5	

华北理工大学实验动物福利伦理委员会

Laboratory Animal Ethical and Welfare Committee of North China University of Science and Technology

主任委员（或授权人）：[illegible]

日期：2024.7.8

地址: 河北　唐山市　曹妃甸新城渤海大道 21 号　邮编：063210
Add: No.21, Bohai Avenue, Caofeidian new town, Tangshan , Hebei Province, Hebei Province, P. R. China. 050017
