# Supplementary material for: Nobiletin ameliorates diabetic nephropathy by modulating TP53-associated PANoptosis: an experimental study
Source: Front Nutr. 2026 Sep 4;13:1816623. doi: 10.3389/fnut.2026.1816623 (PMC13585718; doi:10.3389/fnut.2026.1816623)
Supplement: Supplementary file 1 [file Data_Sheet_1.zip › Data Sheet 1/Supplementary File S2.PDF]

乐美天医药/德思特生物

基于标准·高于标准

Certificate of Analysis

川陈皮素

Nobiletin

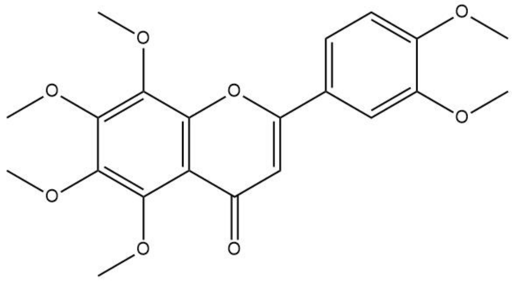

CAS Number:478-01-3

批号(Batch No.): DST230510-037

分子式(M. F.):$C_{21}H_{22}O_8$

分子量(M. W.):402.39

**检验项目（ Test Item）**	**检验指标（ Specifications）**	**检验结果（ Results）**
性状Appearance	White powder	Conforms
纯度Purity (HPLC)	≥98.0%	99.31%
核磁NMR	Comply with the structure	Conforms

检测方法（Test Method）	Column:Kromasil 100-5-C18 4.6*150mm,5μm; Column temperature: 35℃; Detection Mode: UV330nm; Flow Rate:1.0ml/min; Sample dissolution:Methanol; Mobile Phase:A-Methanol,B-0.1% Phosphoric acid in water;Isocratic elution: A,60%.
贮存条件(Storage)	Keep Out of light, dry and stored at -20℃.
提示(Hint)	If you encounter quality problems, please contact us within 15 days after receiving the products and thank you for your purchase.
备注(Note)	The product is only used for scientific research experiments and should not be used for human injection, food or other purposes. This solution must be freshly prepared before using to avoid degradation of the purity of the product and affect the experimental results.

QC: chaonan zhong

QA: Chong chen

成都德思特生物技术有限公司

SpecialSeal:

质检章

Tel：028-85003950 Fax：028-85589956

http://www.lemeitian.cn Add：四川成都温江区海科路西段128号

附件1：HPLC

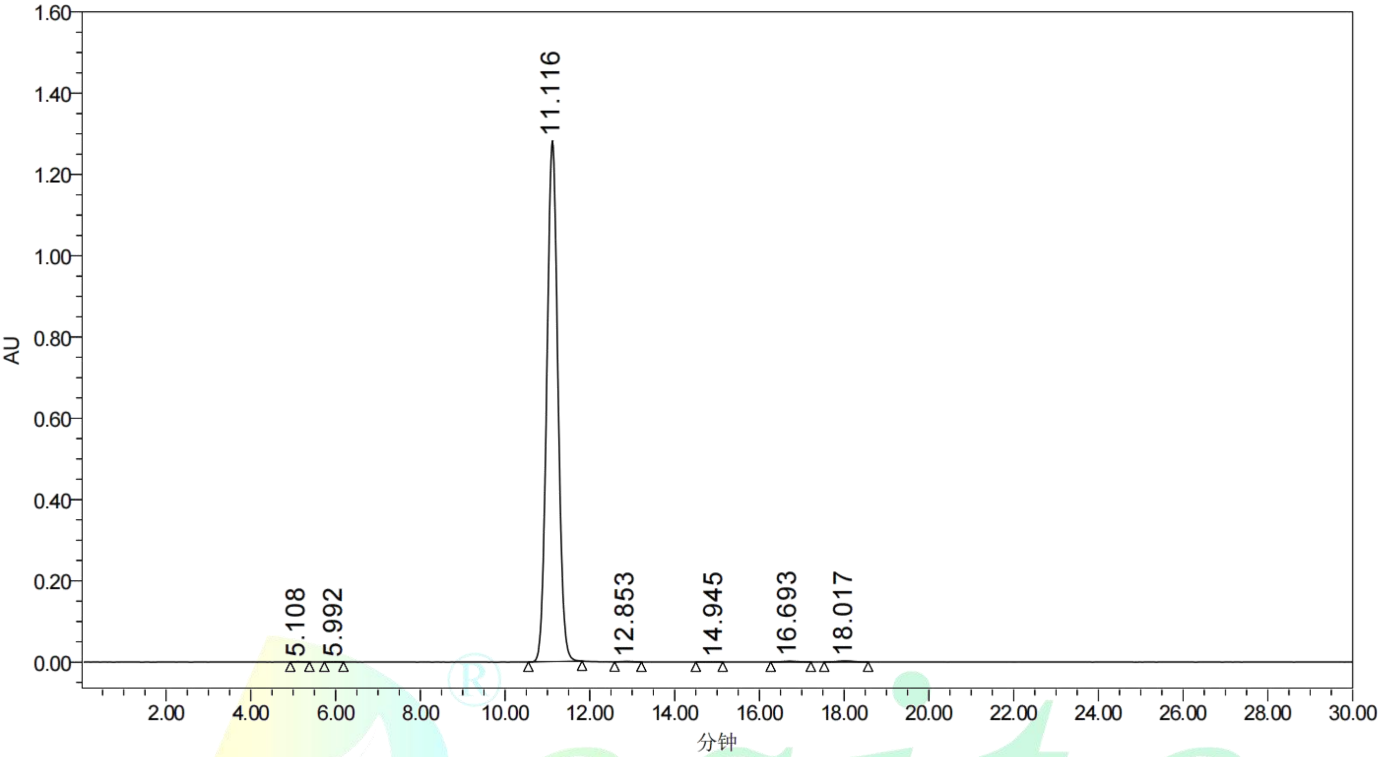

	RT	Area	% Area	Height
1	5.108	10683	0.05	1045
2	5.992	5942	0.03	510
3	11.116	23314203	99.31	1281249
4	12.853	23334	0.10	1263
5	14.945	6478	0.03	318
6	16.693	46426	0.20	1859
7	18.017	69757	0.30	2604

附件2a：^{1}H-NMR

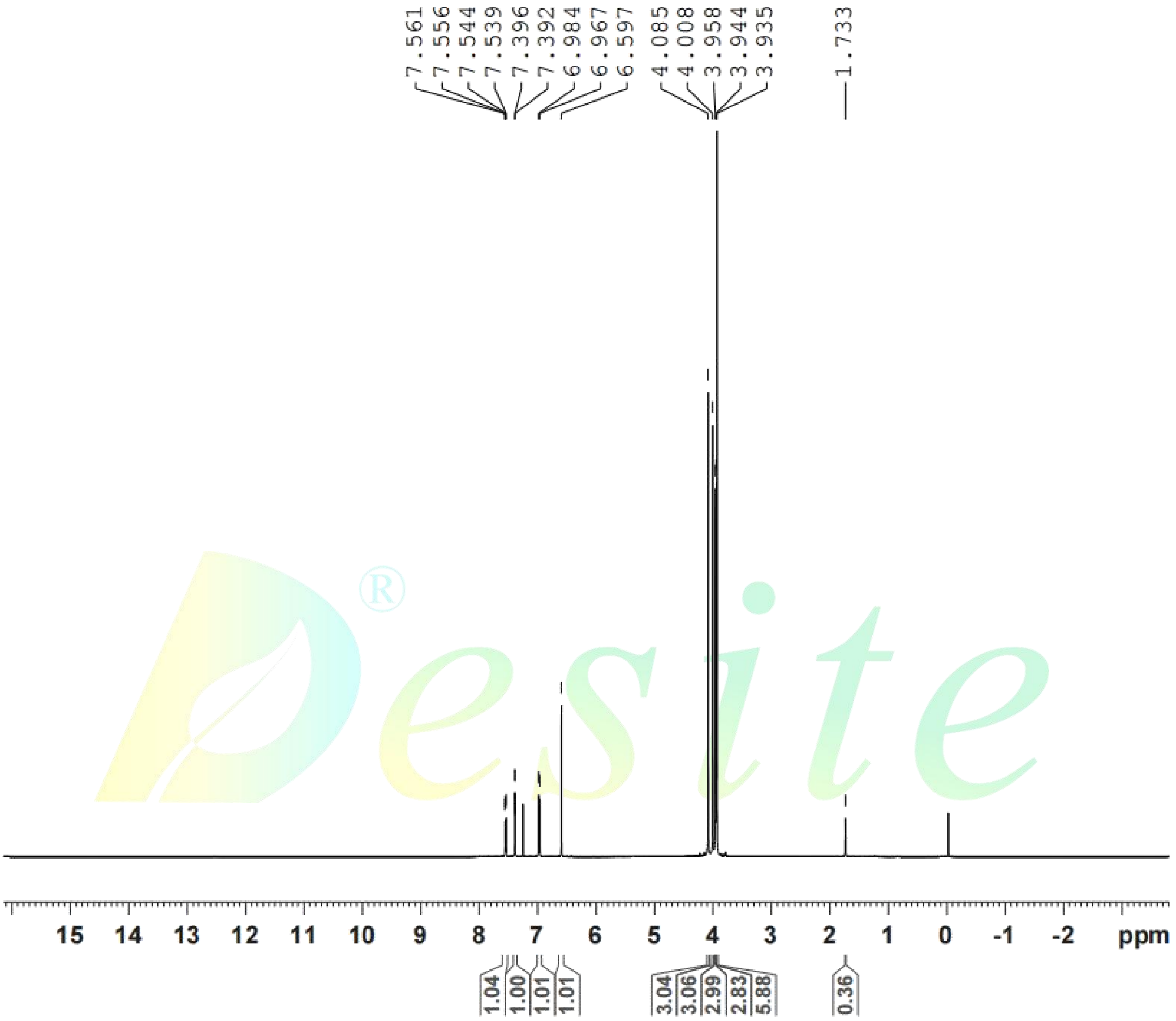
7.561
7.556
7.544
7.539
7.396
7.392
6.984
6.967
6.597
4.085
4.008
3.958
3.944
3.935
1.733
15 14 13 12 11 10 9 8 7 6 5 4 3 2 1 0 -1 -2 ppm
1.04
1.00
1.01
1.01
3.04
3.06
2.99
2.83
5.88
0.36

附件2b：^{13}C-NMR

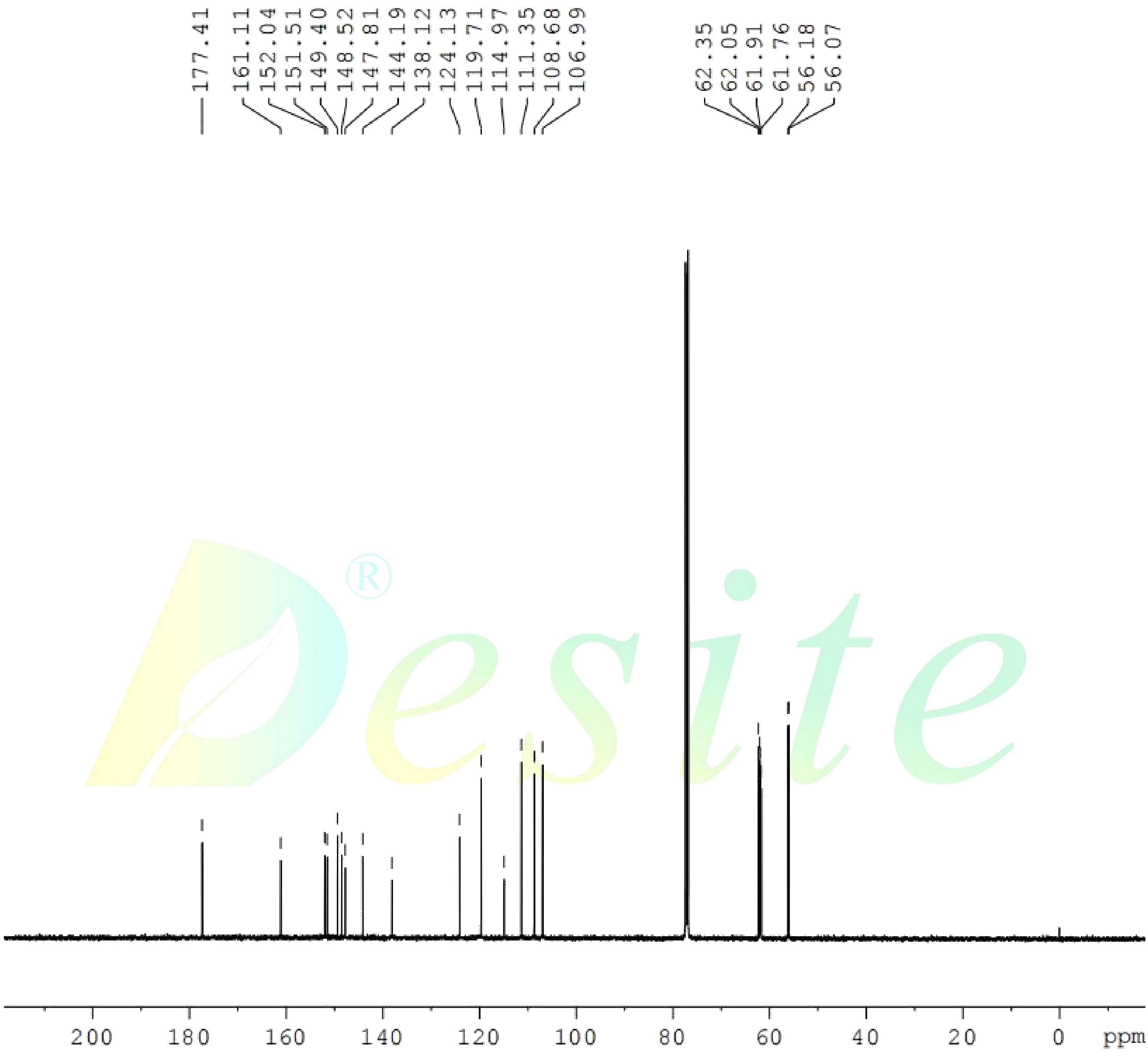

Tel：028-85003950 Fax：028-85589956
http://www.lemeitian.cn Add：四川成都温江区海科路西段128号
